# Efficacy and safety of different polymyxin-containing regimens for the treatment of pneumonia caused by multidrug-resistant gram-negative bacteria: a systematic review and network meta-analysis

**DOI:** 10.1186/s13054-024-05031-w

**Published:** 2024-07-14

**Authors:** Yi Zhou, Guizhong Wang, Ying Zhao, Weijia Chen, Xuyan Chen, Yuqi Qiu, Yuanyu Liu, Shuqi Wu, Jianbin Guan, Ping Chang, Yong Liu, Zhanguo Liu

**Affiliations:** 1 Department of Critical Care Medicine, Zhujiang Hospital, The Second School of Clinical Medicine, Southern Medical University, 253 Gongye Rd, Guangzhou, 510282 China; 2 Department of Intensive Care Unit, Shenzhen Hospital, Southern Medical University, Shenzhen, China

**Keywords:** Polymyxins, Pneumonia, Multidrug-resistant gram-negative bacteria, Network meta-analysis

## Abstract

**Background:**

The optimal administration of polymyxins for treating multidrug-resistant gram-negative bacterial (MDR-GNB) pneumonia remains unclear. This study aimed to systematically assess the efficacy and safety of three polymyxin-containing regimens by conducting a comprehensive network meta-analysis.

**Methods:**

We comprehensively searched nine databases. Overall mortality was the primary outcome, whereas the secondary outcomes encompassed microbial eradication rate, clinical success, acute kidney injury, and incidence of bronchospasm. Extracted study data were analyzed by pairwise and network meta-analyses. Version 2 of the Cochrane risk‐of‐bias tool and the Risk of Bias in Nonrandomized Studies of Interventions (ROBINS‐I) assessment tool were used to assess the risk of bias in randomized trials and cohort studies, respectively.

**Results:**

This study included 19 observational studies and 3 randomized controlled trials (RCTs), encompassing 3318 patients. Six studies with high risk of bias were excluded from the primary analysis. In the pairwise meta-analysis, compared to the intravenous (IV) polymyxin-containing regimen, the intravenous plus inhaled (IV + IH) polymyxin-containing regimen showed a significant decrease in overall mortality, while no statistically significant difference was found in the inhaled (IH) polymyxin-containing regimen. The network meta-analysis indicated that the IV + IH polymyxin-containing regimen had significantly lower overall mortality (OR 0.67; 95% confidence interval [CI] 0.50–0.88), higher clinical success rate (OR 1.90; 95% CI 1.20–3.00), better microbial eradication rate (OR 2.70; 95% CI 1.90–3.90) than the IV polymyxin-containing regimen, and significantly better microbial eradication rate when compared with the IH polymyxin-containing regimen (OR 2.30; 95% CI 1.30–4.20). Furthermore, compared with IV + IH and IV polymyxin-containing regimens, the IH polymyxin-containing regimen showed a significant reduction in acute kidney injury.

**Conclusions:**

Our study indicates that among the three administration regimens, the IV + IH polymyxin-containing regimen may be the most effective for treating MDR-GNB pneumonia, with a significantly lower overall mortality compared to the IV regimen and a considerably higher microbial eradication rate compared to the IH regimen. The IH regimen may be considered superior to the IV regimen due to its substantially lower incidence of acute kidney injury, even though the reduction in overall mortality was not significant.

**Supplementary Information:**

The online version contains supplementary material available at 10.1186/s13054-024-05031-w.

## Background

Multidrug-resistant gram-negative bacterial (MDR-GNB) pneumonia is highly prevalent in intensive care unit (ICU) [1, 2]. Epidemiological investigations have documented a substantial occurrence of MDR pneumonia within hospital environments, ranging from 15 to 24% [3, 4]. Moreover, approximately 2.3 million patients worldwide perish annually due to MDR pneumonia as revealed by a recent Global Burden of Disease study [5]. *Klebsiella pneumoniae* (*K*. *pneumoniae*), *Pseudomonas aeruginosa* (*P*. *aeruginosa*), and *Acinetobacter baumannii* (*A. baumannii*) have been reported to be the most prevalent pathogens causing MDR-GNB pneumonia [6, 7]. Polymyxins, which act as microbicides by cleaving the bacterial cell membrane [8], were previously discarded from clinical practice owing to severe side effects, including nephrotoxicity [9]. However, with the recent development of antibiotic resistance, polymyxins have regained attention as an effective drug against MDR-GNB [10]. Nonetheless, the efficacy of intravenous (IV) polymyxin-containing regimen in treating MDR-GNB pneumonia is restricted because of its limited penetration into the lung parenchyma [11, 12]. Consequently, to achieve improved therapeutic outcomes, inhaled (IH) polymyxin-containing regimen has been explored as an adjunct or alternative to IV polymyxin-containing regimen [13, 14].

The evidence supporting the use of IH polymyxin-containing regimen remains insufficient, and the relevant studies remain controversial [15–18]. The European Society of Clinical Microbiology and Infectious Diseases pointed out that IH polymyxin substitution administration without IV polymyxin has restricted systemic distribution, potentially allowing for elevated concentrations in lung tissue while minimizing systemic toxicity. It's crucial to conduct randomized clinical trials (RCTs) in the future to evaluate the effectiveness of the substitution administration strategy. [16, 19]. There has been no meta-analysis regarding IH polymyxin substitution administration. Therefore, this study aimed to update the evidence and conduct a systematic assessment of the efficacy and safety of three polymyxin-containing regimens (IV, IH, and IV + IH polymyxin-containing regimens) in treating MDR-GNB pneumonia using network meta-analysis methods.

## Methods

We performed a comprehensive systematic review along with a Bayesian network meta-analysis compliant with the Preferred Reporting Items for Systematic Reviews and Meta-analysis extension statement for network meta-analysis [20] (Additional file 1). The protocol is registered at the international prospective register of systematic reviews (PROSPERO registration CRD42023484669).

### Search strategy

We systematically searched nine electronic databases from their inception to November 15, 2023, which included four English databases (Web of Science, EMBASE, PubMed, and the Cochrane Library), two major clinical research registration websites (ClinicalTrials and World Health Organization International Clinical Trials Registry Platform), two preprint websites (medRxiv and Social Science Research Network), and one conference paper database (OCLC FirstSearch [Proceedings, PapersFirst]). The search was restricted exclusively to studies involving human subjects, with no language restrictions applied. To finish the search, the following keywords and medical subject heading phrases were combined: “inhalation”; “infusions, intravenous,” “administration, intravenous,” or “injections, intravenous”; “colistin,” “polymyxin B,” or “polymyxins”; and “pneumonia.” Furthermore, to guarantee a comprehensive identification of all qualified studies, reference lists of recent reviews and related primary studies were manually searched. The details of the search strategy are presented in Appendix 1, Additional file 2.

### Selection criteria

To decide whether the literature matched the eligibility criteria, four reviewers independently evaluated the titles, abstracts, and complete texts of the studies. Disagreements were resolved through discussions among reviewers. The screening of studies was completed according to the PICOS principle as follows:

1. Participant: adult patients with pneumonia due to MDR-GNB; Pathogens were considered MDR if they were non-susceptible to at least one agent in three or more antimicrobial categories to which the pathogen would typically be susceptible [21].
2. Intervention: polymyxins by IV injection, IH, or IH plus IV injection, combined with conventional drugs such as other antibiotics;
3. Comparator: any of the above three polymyxin administration routes;
4. Outcomes: at least one of the outcomes of interest was covered;
5. Study type: RCTs and cohort studies.

Studies meeting the following conditions were excluded: (1) case reports, reviews, meta-analyses, and letters; (2) animal experimental studies; (3) studies with incomplete original data; and (4) duplicate published studies.

Overall mortality was the primary outcome of interest. When multiple mortalities are simultaneously reported in a single article, the mortality for the longest follow-up period will be included. The following were the secondary outcomes:

1. Microbial eradication rate: no baseline pathogen growth was observed on the culture medium after administration [22].
2. Clinical success: clinical success includes clinical cure and clinical improvement, which specifically involve the resolution of clinical and biological signs of infection as defined in individual studies. [17, 23]. The specific definition of clinical success in each included study is provided in Appendix 9, Additional file 2.
3. Acute kidney injury: acute kidney injury is defined as an increase in serum creatinine (SCr) of ≥ 0.3 mg/dL (≥ 26.5 μmol/L) within 48 h; or an increase in serum creatinine to ≥ 1.5 times baseline, which is known or presumed to have occurred within the previous 7 days; or a urine volume of < 0.5 mL/kg/h for 6 h. [24, 25]. The specific definition in each included study is provided in Appendix 10, Additional file 2.
4. Incidence of bronchospasm: the proportion of patients with bronchospasm.

### Data extraction and risk of *bias* assessment

Two researchers independently extracted data from the included studies. All disagreements were resolved through discussion with the third researcher. The extracted data included study characteristics (title, author, year of publication, and study type), participant characteristics (gender, age, and sample size), details of treatment in the intervention and controls, and outcomes.

Evaluation of the quality of the included studies was conducted by four investigators, respectively. According to Version 2 of the Cochrane risk‐of‐bias tool for randomized trials, the overall risk of bias of the included RCTs was classified as low risk, some concerns, and high risk [26]. According to the Risk of Bias in Nonrandomized Studies of Interventions (ROBINS‐I) assessment tool [27], the overall risk of bias for the included cohort studies was classified as low, moderate, serious, and critical. The risk of bias assessment was completed strictly following the algorithm and guide proposed by the tools used. The cohort studies with a serious or critical risk of bias and RCTs with a high risk of bias were excluded, after which the outcomes of the remaining studies were incorporated into the primary data analysis.

### Data synthesis and analysis

Statistical analyses were performed using R version 4.3.1 and RStudio Desktop using the *meta, gemtc,* and *igraph* packages. For every outcome and pair of interventions, we computed the odds ratios (ORs) and matching 95% confidence intervals (CIs).

The primary analysis was based on trials after the exclusion of studies adjudicated as having a high risk of bias. Firstly, we conducted a conventional pairwise meta-analysis for all comparisons. To assess the existence of statistically significant heterogeneity in these studies, the χ^2^ test was employed, whereas to measure the degree of heterogeneity, we used the inconsistency index (I^2^). Study-level data were aggregated using the DerSimonian–Laird random effect model when I^2^ > 50% or the Mantel–Haenszel common effect model when I^2^ ≤ 50%. The Z test with 95% CIs was used to evaluate the significance of the pooled ORs. Potential publication bias was scrutinized using funnel plots, whereas the asymmetry in the funnel plots was assessed using Egger's test.

Furthermore, we included articles exhibiting a high risk of bias for sensitivity analysis and compared these results with those of the primary analysis. To investigate the impact of different polymyxins administration regimens on short-term overall mortality (mortality at longest follow-up within 30 days), we conducted sensitivity analyses that encompassed studies reporting pertinent outcomes. We used best- and worst-case analyses to evaluate the potential impact of missing outcome data [28]. The best-and-worst-case scenarios assumed that all patients who missed outcome assessments in the intervention group had a beneficial outcome, while those who missed outcome assessments in the control group had a detrimental outcome. Conversely, in the worst-best-case scenario, we assumed that all patients in the intervention group who were lost to outcome assessment had a negative outcome, while patients in the control group who were lost to outcome assessment had a positive outcome. We conducted subgroup analyses based on study type to assess heterogeneity, and further explored the influence of polymyxins type and nebulizer type on the analysis outcomes through subgroup analyses. Additionally, subgroup analyses were performed according to pathogen species to assess whether there were differences in the response to polymyxins among different pathogens. To ensure comparability of baseline disease severity, we performed a meta-analysis of baseline data on the APACHE II score. If the *P* value fell below 0.05, it was regarded as statistically significant.

Using the *igraph* and *gemtc* packages in R, we performed a network meta-analysis of three interventions. A multiple treatments comparison was performed through a Bayesian network framework with a Monte Carlo Markov Chain model, employing a consistency model. The optimal model was selected according to the deviance information criterion, which suggested a significant improvement in model fit, with a 2–3 point decrease in value [29]. For each set of chains, 100,000 updates were generated, and the first 100,000 iterations were eliminated as the burn-in phase. Model convergence was assessed using the Brooks–Gelman–Rubin diagnostic plot as well as the trace and density plots. The node splitting test method was used to analyze the consistency of direct and indirect comparisons. A *P* value of ≥ 0.05 suggests that the model’s consistency is satisfactory. We assessed the model’s overall heterogeneity using the “anohe” function for calculating the deviation of the size of the heterogeneity variance parameter I^2^. Moreover, in order to determine the relative rankings of different treatments according to the surface under the cumulative ranking curve (SUCRA), Bayesian network meta-analysis estimates were reported as rank probabilities [30], which range from 0% (indicating statistical certainty as the worst treatment) to 100% (indicating statistical certainty as the best treatment). Of note, SUCRA rankings are only relative values.

### Assessment of the quality of evidence

The quality of evidence was assessed using the Grading of Recommendations, Assessment, Development, and Evaluation (GRADE) method with the GRADEpro GDT online tool [31, 32]. During the rating procedure, the GRADE Handbook and guidelines were strictly followed [33, 34].

## Results

### Study selection and risk of *bias* assessment

**Fig. 1:**
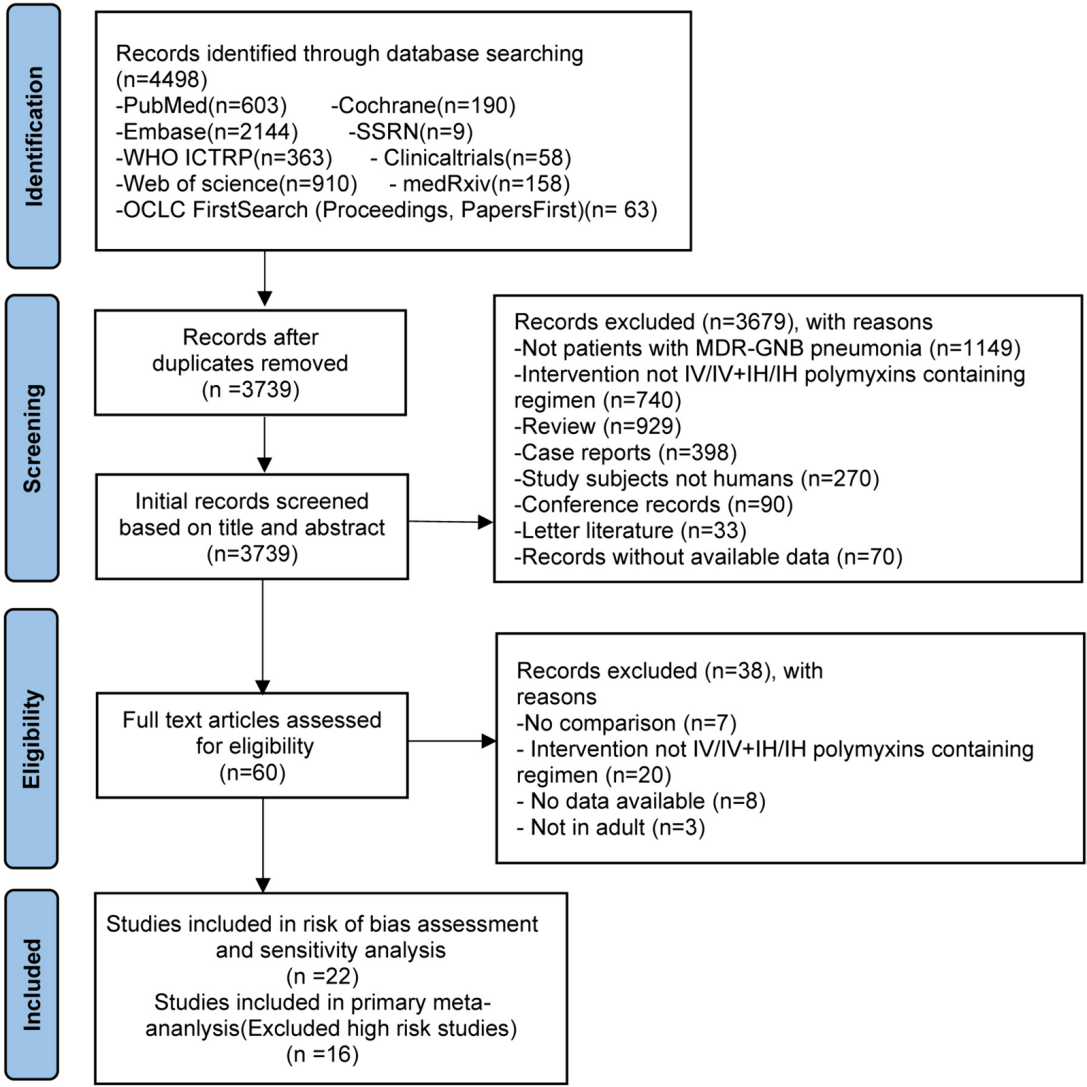
Study flow diagram

By searching electronic databases, preprint sites, and clinical registry sites, 4498 results were retrieved. Excluding duplicates, 3739 results remained. After reading the title, abstract, and full text of each article, 3717 that did not fit the inclusion criteria were eliminated, leaving 22 articles that were ultimately chosen (Fig. 1). Appendix 2, Additional file 2 displays the results of the risk of bias assessment for the included studies. The included 3 RCTs were at high, medium, and low risks of bias, respectively. One prospective cohort study was at medium risk of bias. Of the 18 retrospective cohort studies, 5 were at high risk of bias, 6 were at medium risk of bias, and 7 were at low risk of bias. The most common reason for the high risk of bias was baseline imbalance, while other causes included missing data and selection of the reported results. We excluded 6 high-risk studies and ultimately included 16 studies in the primary analysis [14, 17, 18, 22, 25, 35–45]. Eleven studies compared IV polymyxins with IV + IH polymyxins, three compared IV polymyxins with IH polymyxins, one contrasted IH polymyxins with IV + IH polymyxins, and one article comprehensively evaluated all three administration regimens.

### Study characteristics

Table 1 presents the characteristics of the studies included in the primary meta-analysis. These studies were from ten countries and regions, and the patients' mean age ranged from 48.19 to 73 years. The pathogens of the infected individuals were mainly *A. baumannii*, *P. aeruginosa*, and *K. pneumoniae*. Treatment duration ranged from 10 to 16.9 days, with one study having a treatment duration of > 14 days without an upper limit. Table 2 presents the baseline disease severity of the study patients, including the disease severity score (including SOFA score, APACHE II score, SAPS II score and CPIS) and the number and proportion of patients with sepsis or septic shock.

**Table 1:** Characteristics of studies included in the meta-analysis (excluding high-risk studies)

Author, year	Study type	Pathogen	Group	No.of patients	Age (mean ± SD years)	Sex male/female	Total daily dose	Concomitant antibiotics (IV)	Polymyxins treatment duration (day)	Type of polymyxins	Device for drug delivery	Follow up period (day)^b^	Follow up period (day)^c^	Country
Abdellatif, 2016 [17]	RCT	S. maltophilia, P. aeruginosa, Enterobacteria, A. baumannii	IH	73	50 ± 16	NA	(Loading dose: 9 MU) + 9 MU	Imipenem	14	CMS	Ultrasonic vibrating plate nebulizer	28	28	Tunisia
			IV	76	53 ± 17	NA	12 MU	Imipenem	14			28	28	
Ahn, 2020 [35]	Retrospective cohort study	A. baumannii, P. aeruginosa	IV	29	66.55 ± 14.48	19/10	NA	NA	14.8 ± 8.3	NA	NA	NA	NA	Korea
			IH	59	69.59 ± 15.98	35/24	NA	NA	15.8 ± 9.5			NA	NA	
Almangour, 2021 [22]	Retrospective cohort study	A. baumannii, P. aeruginosa, *K. pneumoniae*	IV	70	54 ± 18	49/21	7.5 ± 2.5 MIU	Carbapenem,Piperacillin/tazobactam,Tigecycline,Cephalosporin,Fluoroquinolone	IV: 11 ± 6	CMS	Jet nebulizers or vibrating mesh nebulizer	110 ± 105	NA	Saudi Arabia
			IV + IH	65	54 ± 20	44/21	IV: 8 ± 2.8 MIU IH: 6.5 ± 2.5 MIU	Carbapenem, Piperacillin/tazobactam, Tigecycline, Cephalosporin, Fluoroquinolone	IV: 11.5 ± 6			115 ± 98	NA	
Amin, 2013 [36]	Prospective cohort study	A. baumannii, P. aeruginosa, *K. pneumoniae*	IV	12	60.5 ± 4.5	7/5	3–8 MIU	NA	IV: 14.1 ± 9.4	CMS	Conventional nebulizers	NA	NA	Egypt
			IV + IH	28	55.6 ± 21.9	15/13	IV: 3–8 MIU IH: 4 MIU	NA	IV: 15.3 ± 8.7			NA	NA	
Bogović, 2014 [37]	Retrospective cohort study	P. aeruginosa, A. baumannii, *K. pneumoniae*	IV	23	72.5 ± 12.91	14/9	9 MIU	NA	16.9 ± 15.10	NA	Conventional nebulizers	33.8 ± 21.88	NA	Greece
			IV + IH	8	72.4 ± 11.87	5/3	IV: 9 MIU IH: 4 MIU	NA	10.3 ± 5.72			30.5 ± 11.56	NA	
Choe, 2019^a^ [38]	Retrospective cohort study	A. baumannii, P. aeruginosa, *K. pneumoniae*	Non- LD IV	70	68 (62–74)	46/24	2.9 (2.1–4.3) mg/kg	Carbapenem, Piperacillin/Tazobactam, Minocycline, Tigecycline, Vancomycin	IV: 14 (10–15)	CMS	Ultrasonic nebulizer or jet nebulizer	90	30	Korea
			LD IV	86	63 (54–75)	64/22	3.9 (2.9–5.0) mg/kg	Carbapenem, Piperacillin/Tazobactam, Minocycline, Tigecycline, Vancomycin	IV: 14 (9–15)			90	30	
			IV + IH	35	67 (54–76)	31/4	IV: 3.1 (2.2–4.1) mg/kg IH: 450 mg CBA	Carbapenem, Piperacillin/Tazobactam, Minocycline, Tigecycline, Vancomycin	IV: 14 (12–17)			90	30	
Hasan, 2021 [18]	RCT	*K. pneumoniae*	IV	57	63.9 ± 14.3	33/24	(loading dose: 20,000–25,000 IU/kg) + 20,000–25,000 IU/Kg	NA	IV: > 14	Polymyxin B	NA	30	30	Bengla
			IV + IH	64	64.1 ± 16.1	37/27	IV: (loading dose: 20,000–25,000 IU/kg) + 20,000–25,000 IU/Kg IH: 1 MU	NA	IV: > 14			30	30	
Kalin, 2012 [39]	Retrospective cohort study	A. baumannii	IV	16	48.19 ± 22.21	13/3	5 or 10 mg/kg	Glycopeptide, Aminoglycoside	14	CMS	NA	NA	NA	Japan
			IV + IH	29	51.10 ± 19.78	19/10	IV: 5 or 10 mg/kg IH: 150 mg	Glycopeptide, Aminoglycoside	14			NA	NA	
Kim, 2017 [40]	Retrospective cohort study	A. baumannii	IV	39	66 (52–77)	30/9	250 (175–300)mg CBA	Carbepenems, Tigecycline, Minocycline, Ampicillin/sulbactam	10 (7–22)	CMS	Conventional jet nebulizer	23 (19–40)	23 (19–40)	Korea
			IH	39	67 (58–74)	22/17	300 (300–300)mg CBA	Carbepenems, Tigecycline, Minocycline, Ampicillin/sulbactam	14 (9–24)			22 (15–34)	22 (15–34)	
Korbila, 2010 [14]	Retrospective cohort study	A. baumannii, P. aeruginosa, *K. pneumoniae*	IV	43	60.9 ± 15.7	31/12	(6.4 ± 2.3)MIU	Antipseudomonal penicillin, Aminoglycosides, Fluoroquinolones, Carbapenems, Rifampicin	13.7 ± 11.2	NA	Conventional nebulizer	NA	NA	Greece
			IV + IH	78	59.2 ± 19.2	61/17	IV: (7.0 ± 2.4)MIU IH: (2.1 ± 0.9)MIU	Antipseudomonal penicillin, Aminoglycosides, Fluoroquinolones, Carbapenems, Rifampicin	16.9 ± 9.8			NA	NA	
Zhou, 2021 [41]	Retrospective cohort study	A. baumannii, *K. pneumoniae*, P. aeruginosa, E. cloacae	IV	25	66.8 ± 13.6	20/5	(loading dose: 2.0 mg/kg) + 2.5 mg/kg	Tigecycline, minocycline, meropenem, Imipenem cilastatin, piperacillin tazobactam, ceftazidime, levofloxacin, teicoplanin	15.2 ± 5.4	Polymyxin B	Jet nebulizer	NA	NA	China
			IV + IH	20	59.2 ± 14.2	14/6	IV: (loading dose: 2.0 mg/kg) + 2.5 mg/kg IH: 50 mg	Tigecycline, meropenem, piperacillin tazobactam, ceftazidime, levofloxacin	IV: 15.2 ± 6.7 IH: 12.3 ± 5.3			NA	NA	
Lin, 2022 [42]	Retrospective cohort study	P. aeruginosa, A. baumannii, *E. coli*, *S. aureus*	IV	40	60.77 ± 3.36	32/8	IV: (loading dose: 2.0 mg/kg) + 2.5 mg/kg	Routine broadspectrum antibiotics	NA	Polymyxin B	NA	NA	NA	China
			IV + IH	44	60.36 ± 2.98	29/15	IV: (loading dose: 2.0 mg/kg) + 2.5 mg/kg IH: 50 mg/kg	Routine broadspectrum antibiotics	NA			NA	NA	
Liu, 2022 [43]	Retrospective cohort study	*E. coli*, *K. pneumoniae*, A. baumannii, P. aeruginosa	IV	88	64 ± 17	69/19	(loading dose: 2.0 (1.7, 2.1) mg/kg) + 2.0 (2.0, 3.0) mg/kg	NA	12 ± 9	Polymyxin B	Vibrating mesh nebulizer	33 (18–54)	28	China
			IV + IH	44	67 ± 17	34/10	IV: (loading dose: 2.0 (1.7, 2.1) mg/kg) + 2.0 (2.0, 2.5) mg/kg IH: 1.82 (1.04, 2.0) mg/kg	NA	10 ± 7			35 (23–55)	28	
Matijašević, 2018 [44]	Retrospective cohort study	A. baumannii	IV	42	NA	NA	NA	NA	NA	NA	NA	28	28	Serbia
			IV + IH	27	NA	NA	NA	NA	NA			28	28	
Wu, 2023 [25]	Retrospective cohort study	A. baumannii, P. aeruginosa, *K. pneumoniae*, Enterobacterales	IH	39	59.9 ± 18.0	23/16	100 mg polymyxin B or 60–120 mg CBA polymyxin E	Quinolones, β-lactamases, Carbapenem, Aminoglycosides, Vancomycin	NA	Polymyxin B, polymyxin E	Ultrasonic vibrating plate nebuliser	14 (7–26)	14 (7–26)	China
			IV + IH	39	62.5 ± 13.6	19/20	IV: 2.5–3.0 mg/kg polymyxin B or 300–360 mg CBA polymyxin E IH: 100 mg polymyxin B or 60–120 mg CBA polymyxin E	Quinolones, β-lactamases, Carbapenem, Aminoglycosides, Vancomycin	NA			11 (7–20)	11 (7–20)	
Shi, 2023 [45]	Retrospective cohort study	A. baumannii, P. aeruginosa, *K. pneumoniae*	IV	38	62 ± 17	30/8	(loading dose: 2.0–2.5 mg/kg) + 2.5–3 mg/kg	Beta-lactam, Quinolones, Tetracycline, Glycopeptides, Aminoglycosides, Linezolid, Antifungal drugs	12 (6–17)	Polymyxin B	Vibrating mesh nebulizer	90	28	China
			IH	38	73 ± 16	31/7	100 mg	Beta-lactam, Quinolones, Tetracycline, Glycopeptides, Aminoglycosides, Linezolid, Antifungal drugs	11 (8–17)			90	28	
			IV + IH	35	68 ± 17	27/8	IV: (loading dose: 2.0–2.5 mg/kg) + 2.5–3 mg/kg IH: 100 mg	Beta-lactam, Quinolones, Tetracycline, Glycopeptides, Aminoglycosides, Linezolid, Antifungal drugs	14 (9–24)			90	28	

RCT, randomized controlled trial; *A. baumannii*, *Acinetobacter baumannii*; *P. aeruginosa*, *Pseudomonas aeruginosa*; *S. maltophilia*, *Stenotrophomonas maltophilia*; *S. aureus*, *Staphylococcus aureus*; *E. cloacae, Enterobacter cloacae; E. coli, Escherichia coli; K. pneumoniae*, *Klebsiella pneumoniae*; SD, standard deviation; NA, not applicable; MU, million units; MIU, million international units; IU, international units; CBA, colistin base activity; CMS, colistimethate sodium; LD, loading dose

^a^When conducting the pairwise and network meta-analysis, the non-LD IV and LD IV groups were merged into IV group

^b^Follow up period for overall mortality outcome

^c^Follow up period for short-term overall mortality outcome

**Table 2:** Baseline disease severity of study patients

Author, year	Group	Disease severity	Patients with sepsis/septic shock n (%)
Abdellatif, 2016 [17]	IH	SOFA score: 7.03 ± 3.8	NA
	IV	SOFA score: 6.5 ± 4.1	NA
Ahn, 2020 [35]	IV	APACHE score: 9.17 ± 5.70	Sepsis: 13 (44.8)
	IH	APACHE score: 9.23 ± 7.88	Sepsis: 24 (40.7)
Almangour, 2021 [22]	IV	APACHE II score: 18 ± 6	Septic shock: 15 (21)
	IV + IH	APACHE II score: 17.5 ± 5	Septic shock: 15 (23)
Amin, 2013 [36]	IV	APACHE II score: 19.1 ± 7	NA
	IV + IH	APACHE II score: 18.1 ± 5	NA
Bogović, 2014 [37]	IV	SAPS II score: 49.0 ± 11.62	Sepsis: 11 (47.8) Septic shock: 12 (52.2)
	IV + IH	SAPS II score: 53.3 ± 13.93	Sepsis: 5 (62.5) Septic shock: 3 (37.5)
Choe, 2019^a^ [38]	Non-LD IV	SOFA score: 7 (5–10)	NA
	LD IV	SOFA score: 8 (4–11)	NA
	IV + IH	SOFA score: 8 (4–12)	NA
Hasan, 2021 [18]	IV	APACHE II score: 18.3 ± 5.5	NA
	IV + IH	APACHE II score: 18 ± 4.8	NA
Kalin, 2012 [39]	IV	APACHE II score (median): 22	Sepsis: 11 (69) Septic shock: 4 (25)
	IV + IH	APACHE II score (median): 22	Sepsis: 18 (62) Septic shock: 6 (21)
Kim, 2017 [40]	IV	APACHE II score: 20 (16–24) CPIS: 6 (5–7)	Septic shock: 58 (62)
	IH	APACHE II score: 21 (19–24) CPIS: 6 (5–7)	Septic shock: 57 (45)
Korbila, 2010 [14]	IV	APACHE II score: 19.2 ± 7	NA
	IV + IH	APACHE II score: 17.4 ± 6	NA
Zhou, 2021 [41]	IV	APACHE II score: 15.0 (14.0, 17.5)	NA
	IV + IH	APACHE II score: 15.0 (8.2, 21.8)	NA
Lin, 2022 [42]	IV	APACHE II score: 14.98 ± 2.44	NA
	IV + IH	APACHE II score: 14.36 ± 2.28	NA
Liu, 2022 [43]	IV	APACHE II score: 20 ± 5 SOFA score: 8 ± 4	Sepsis or septic shock: 50(56.8)
	IV + IH	APACHE II score: 18 ± 7 SOFA score: 9 ± 4	Sepsis or septic shock: 24(54.5)
Matijašević, 2018 [44]	IV	NA	NA
	IV + IH	NA	NA
Wu, 2023 [25]	IH	SOFA score: 6.5 (3–9) CPIS: 7 (6–7.25)	NA
	IV + IH	SOFA score: 7 (4–10) CPIS: 7 (6–8)	NA
Shi, 2023 [45]	IV	APACHE II score: 20 ± 6 SOFA score: 8 (5–11) CPIS: 7 ± 2	Septic shock: 23(60.5)
	IH	APACHE II score: 21 ± 4 SOFA score: 7 (5–10) CPIS: 6 ± 2	Septic shock: 26(68.4)
	IV + IH	APACHE II score: 21 ± 6 SOFA score: 6 (5–8) CPIS: 7 ± 2	Septic shock: 29(82.9)

IV + IH, intravenous plus inhaled polymyxins; IV, intravenous polymyxins; IH, inhaled polymyxins; NA, not applicable; SOFA, Sequential Organ Failure Assessment; APACHE, Acute Physiology and Chronic Health Evaluation; SAPS, Simplified Acute Physiology Score; CPIS, Clinical Pulmonary Infection Score; LD, loading dose

^a^When conducting the pairwise and network meta-analysis, the non-LD IV and LD IV groups were merged into the IV group

**Fig. 2:**
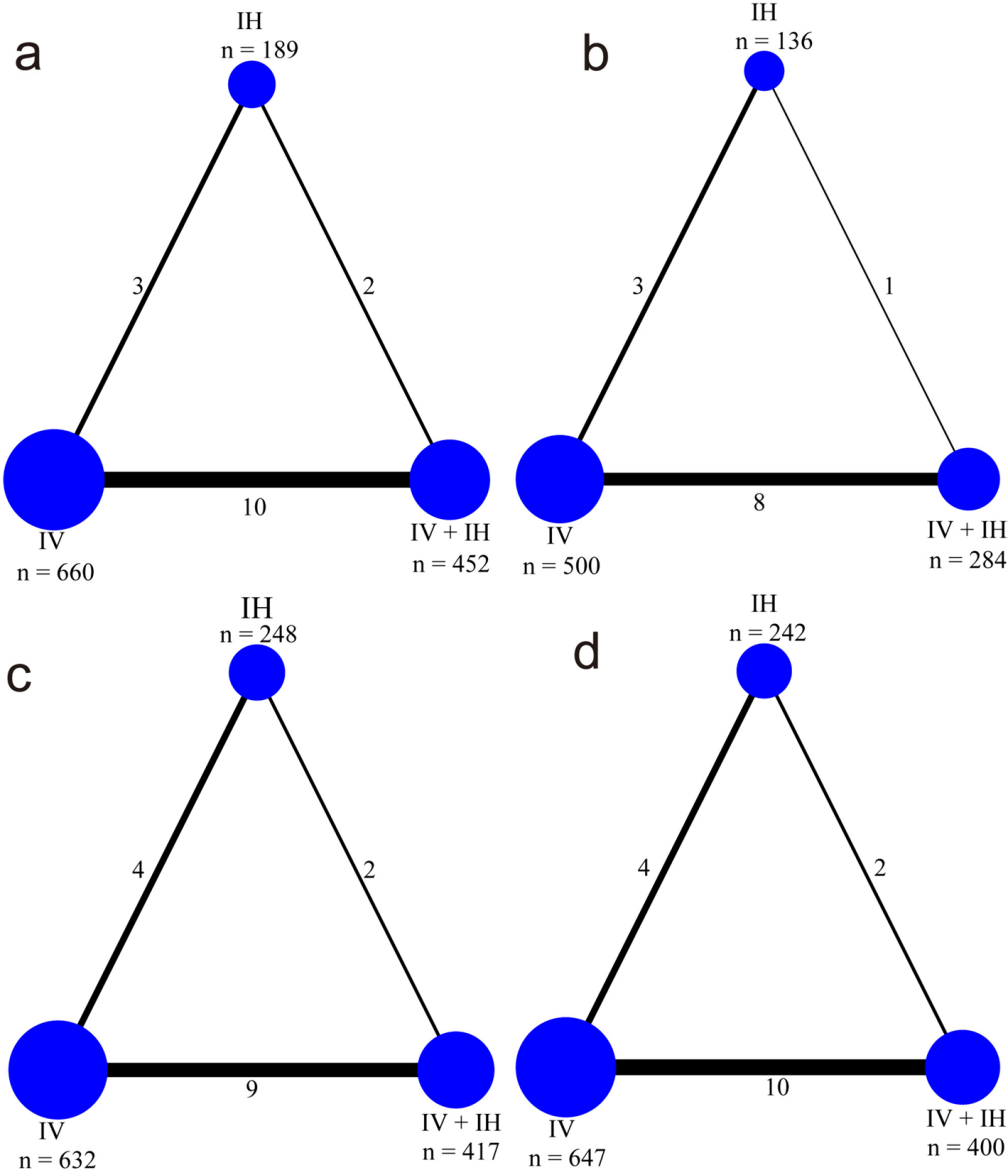
Network graphs of all available comparisons between the eligible interventions. The size of the nodes corresponds to the number of patients administered that intervention. The number on the edges shows the number of trials for each comparison. **a** Overall mortality, **b** microbial eradication rate, **c** clinical success, and **d** acute kidney injury. IV + IH, intravenous plus inhaled polymyxins; IV, intravenous polymyxins; IH, inhaled polymyxins

Of the analyzed studies, 13, 10, 13, 14, and 4 reported overall mortality, microbial eradication rate, clinical success, acute kidney injury, and incidence of bronchospasm, respectively. In the network meta-analysis, three different administration routes were evaluated. Figure 2 illustrates network plots depicting direct comparisons for each outcome, presenting the pairwise comparisons among IV, IV + IH, and IH polymyxin-containing regimens. IV polymyxin-containing regimen also functioned as a bridge node for constructing a closed loop network, which allowed indirect comparisons in the network. Therefore, it was chosen as a usual comparator in the network meta-analysis.

### Results of pairwise *meta*-analysis

**Fig. 3:**
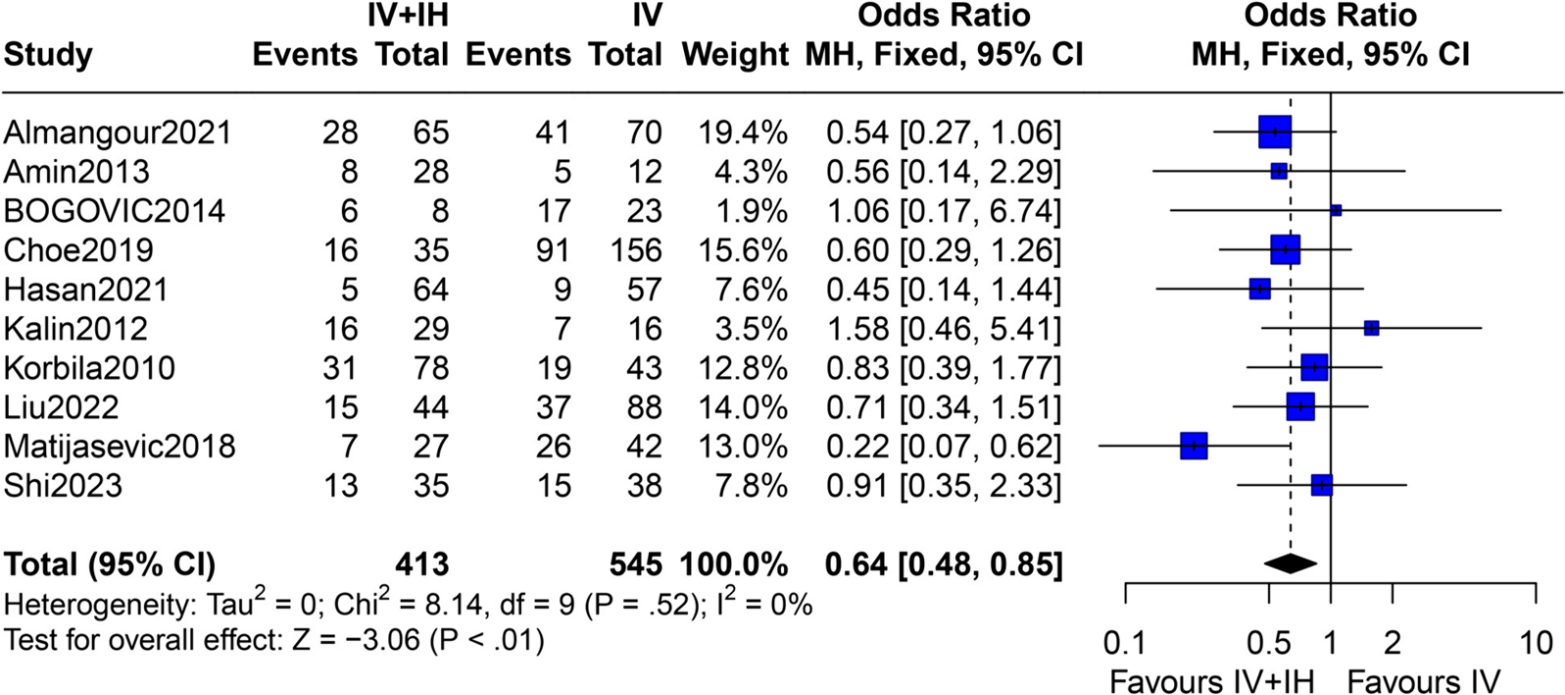
Overall mortality (IV + IH vs. IV excluded high-risk studies). IV + IH, intravenous plus inhaled polymyxins; IV, intravenous polymyxins; CI, confidence interval

**Fig. 4:**
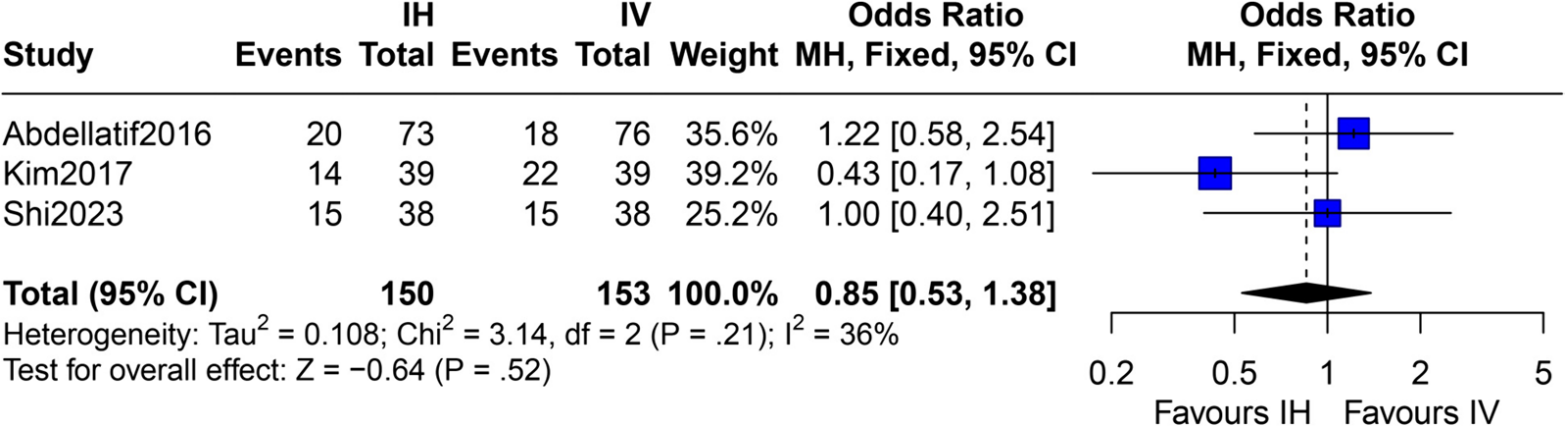
Overall mortality (IH vs. IV excluded high-risk studies). IH, inhaled polymyxins; IV, intravenous polymyxins; CI, confidence interval

In the pairwise comparison, the IV + IH polymyxin-containing regimen was linked to significantly lower overall mortality (total patients: 958, OR = 0.64; 95% CI 0.48–0.85; *p* < 0.01), better clinical success rate (total patients: 866, OR = 1.99; 95% CI 1.46–2.71; *p* < 0.01), and better microbial eradication rate (total patients: 716, OR = 2.75; 95% CI 1.94–3.90; *p* < 0.01) in patients with GNB pneumonia than the IV polymyxin-containing regimen (Fig. 3 and Supplementary Figs. 1–2, Appendix 3, Additional file 2). Compared with the IV polymyxin-containing regimen, the IH polymyxin-containing regimen was associated with a substantially lower acute kidney injury (OR = 0.25; 95% CI 0.16–0.40; *p* < 0.01), whereas the IV + IH polymyxin-containing regimen had no significant difference (OR = 0.93; 95% CI 0.65–1.33; *p* = 0.67) (Supplementary Figs. 3, 6, Appendix 3, Additional file 2). Moreover, no significant differences were found in the overall mortality, clinical success, and microbial eradication rate between the IH and IV polymyxin-containing regimens (Fig. 4 and Supplementary Figs. 4–5, Appendix 3, Additional file 2). To explore the risk of bronchospasm with IH administration, we combined the IH and IV + IH polymyxin-containing regimens for a pairwise meta-analysis with the IV polymyxins-containing regimen. Furthermore, the incidence of bronchospasm was significantly higher in the IH and IV + IH polymyxin-containing regimens (OR = 9.91; 95% CI 2.14–45.93; *p* < 0.01) (Supplementary Figs. 7, Appendix 3, Additional file 2). No significant heterogeneity was noted across the studies. Meta-analysis of baseline data on APACHE II score indicated that baseline disease severity was comparable between groups (Supplementary Figs. 8–9, Appendix 3, Additional file 2). The summary findings are displayed in Table 3 and Appendix 3, Additional file 2.

**Table 3:** Primary pairwise meta-analysis (excluding high-risk studies)

Outcome	Comparison	Pairwise OR		Number of events	Number of patients	Number of studies	Heterogeneity test	
		95% CI	*p* value				I^2^ (%)	*p* value
Overall mortality	IH versus IV	0.85 (0.53, 1.38)	0.52	104	303	3	36	0.21
	IV + IH versus IV	0.64 (0.48, 0.85)	< 0.01	412	958	10	0	0.52
Clinical success	IH versus IV	0.99 (0.65–1.49)	0.95	236	391	4	10	0.34
	IV + IH versus IV	1.99 (1.46, 2.71)	< 0.01	505	866	9	49	0.05
Acute kidney injury	IH versus IV	0.25 (0.16, 0.40)	< 0.01	128	378	4	0	0.81
	IV + IH versus IV	0.93 (0.65, 1.33)	0.67	218	864	10	0	0.44
Microbial eradication rate	IH versus IV	1.09 (0.64, 1.86)	0.74	126	242	3	0	0.44
	IV + IH versus IV	2.75 (1.94, 3.90)	< 0.01	360	716	8	0	0.59
Incidence of bronchospasm	IH/IV + IH versus IV	9.91 (2.14–45.93)	< 0.01	11	433	4	0	0.95

IV + IH, intravenous plus inhaled polymyxins; IV, intravenous polymyxins; OR, odds ratio; IH, inhaled polymyxins; CI, confidence interval

### Sensitivity analysis and subgroup analysis

When incorporating studies with a high risk of bias for sensitivity analysis, the results were consistent with those of the primary pairwise meta-analysis (Supplementary Figs. 10–17, Appendix 4, Additional file 2). The results of the sensitivity analysis of short-term overall mortality were also consistent with the results of the primary meta-analysis (Supplementary Figs. 18–19 Appendix 4, Additional file 2). Regarding microbial eradication rates, we conducted a best- and worst-case analysis, which was also consistent with the results of the primary analysis, suggesting that the loss of access population did not significantly affect the analysis results (Supplementary Figs. 20–21, Appendix 4, Additional file 2). Given the substantial missing data observed in Table 1 of Matijašević et al.'s study [44], a sensitivity analysis was conducted with its exclusion. The result was consistent with that of the primary meta-analysis (Supplementary Figs. 22, Appendix 4, Additional file 2).

Most of the subgroup analyses by study type, polymyxins type, pathogen species, and nebulizer type showed results consistent with that of the primary meta-analysis (Supplementary Figs. 24–28, 31–36, 42, Appendix 4, Additional file 2). Special cases were as follows. First, compared with the IV polymyxin-containing regimen, IV + IH regimen reduced overall mortality in the RCT subgroup (one study), the polymyxin B subgroup (three studies), the *K. pneumoniae* subgroup (one study) and the *A. baumannii* subgroup (two studies). However, the results were not statistically different, as shown in Supplementary Figs. 23, 30, 38, Appendix 4, Additional file 2. Second, compared with IV polymyxin-containing regimen, IH and IV + IH regimens increased the incidence of bronchospasm in the RCT subgroup (one study) and the colistimethate sodium subgroup (one study). However, the results were not statistically different (Supplementary Figs. 29, 37, Appendix 4, Additional file 2). Third, compared with IV polymyxin-containing regimen, IV + IH regimen did not significantly improve the clinical success and microbial eradication rate in the *A. baumannii* subgroup that included only one study by Kalin et al. (Supplementary Figs. 39–40, Appendix 4, Additional file 2) [39]. Fourth, compared with the IV polymyxin-containing regimen, the IV + IH regimen showed a significant reduction in overall mortality when the data from all studies were aggregated. However, this reduction was not statistically significant within each subgroup categorized by nebulizer type (Supplementary Figs. 41, Appendix 4, Additional file 2). Details of subgroup and sensitivity analysis results are shown in Table 4 and Appendix 4, Additional file 2.

**Table 4:** Results of sensitivity and subgroup analyses

Outcome	Comparison		Pairwise OR		Number of events	Number of patients	Number of studies	Heterogeneity test	
			95% CI	*p* value				I^2^ (%)	*p* value
*Sensitivity analysis (all studies that included high-risk studies)*									
Overall mortality	IH versus IV		0.75 (0.52,1.08)	0.12	206	681	7	47	0.08
	IV + IH versus IV		0.63 (0.48,0.82)	< 0.01	481	1122	13	4	0.4
Clinical success	IH versus IV		1.28 (0.74,2.21)	0.37	429	643	7	52	0.05
	IV + IH versus IV		1.94 (1.47,2.56)	< 0.01	587	1030	12	36	0.1
Acute kidney injury	IH versus IV		0.24 (0.17,0.35)	< 0.01	250	729	7	17	0.3
	IV + IH versus IV		0.89 (0.63,1.24)	0.48	240	1028	12	0	0.58
Microbial eradication rate	IH versus IV		1.30 (0.86,1.97)	0.21	289	481	6	36	0.18
	IV + IH versus IV		2.57 (1.90,3.46)	< 0.01	484	942	12	0	0.59
Incidence of bronchospasm	IH/IV + IH versus IV		9.91 (2.14,45.93)	< 0.01	11	433	4	0	0.95
*Sensitivity analysis (best and worst case analysis)*									
Microbial eradication rate	Best case		3.55 (2.53,4.98)	< 0.01	376	773	8	47	0.07
	Worst case		2.05 (1.07,3.90)	0.03	401	773	8	70	< 0.01
*Sensitivity analysis (short-term overall mortality)*									
Short-term overall mortality	IH versus IV		0.85 (0.52,1.38)	0.52	98	303	3	36	0.21
	IV + IH versus IV		0.41 (0.26,0.63)	< 0.01	178	586	5	0	0.58
*Sensitivity analysis (excluded high-risk studies and Matijašević et al.'s study)*									
Overall mortality	IV + IH versus IV		0.70 (0.52,0.95)	0.02	379	889	9	0	0.88
*Subgroup analysis (study type)*									
Overall mortality	IH versus IV	RCT	1.22 (0.58,2.54)	0.6	38	149	1	NA	NA
		Cohort study	0.65 (0.35,1.24)	0.19	66	154	2	38	0.2
	IV + IH versus IV	RCT	0.45 (0.14,1.44)	0.18	14	121	1	NA	NA
		Cohort study	0.65 (0.48,0.88)	< 0.01	398	837	9	0	0.46
Clinical success	IH versus IV	RCT	0.78 (0.39,1.57)	0.49	104	149	1	NA	NA
		Cohort study	1.12 (0.67,1.87)	0.67	132	242	3	26	0.26
	IV + IH versus IV	Cohort study	1.99 (1.46,2.71)	< 0.01	505	866	9	49	0.05
Acute kidney injury	IH versus IV	RCT	0.33 (0.16,0.71)	< 0.01	43	149	1	NA	NA
		Cohort study	0.21 (0.12,0.38)	< 0.01	85	229	3	0	0.96
	IV + IH versus IV	RCT	0.89 (0.17,4.57)	0.88	6	121	1	NA	NA
		Cohort study	0.93 (0.64,1.34)	0.69	212	743	9	10	0.35
Microbial eradication rate	IH versus IV	Cohort study	1.09 (0.64,1.86)	0.74	126	242	3	0	0.44
	IV + IH versus IV	RCT	5.02 (1.71,14.69)	< 0.01	99	121	1	NA	NA
Incidence of bronchospasm	IH/IV + IH versus IV	RCT	8.55 (0.45,162.44)	0.15	4	121	1	NA	NA
		Cohort study	10.58 (1.78,62.83)	< 0.01	7	312	3	0	0.83
*Subgroup analysis (different polymyxins)*									
Overall mortality	IH versus IV	Colistimethate sodium	0.81 (0.46,1.42)	0.45	74	227	2	67	0.08
		Polymyxin B	1.00 (0.40,2.51)	1.00	30	76	1	NA	NA
	IV + IH versus IV	Colistimethate sodium	0.65 (0.42,1.00)	0.05	212	411	4	0	0.49
		Not applicable	0.56 (0.32,0.98)	0.04	106	221	3	57	0.1
		Polymyxin B	0.70 (0.41,1.18)	0.18	94	326	3	0	0.66
Clinical success	IH versus IV	Colistimethate sodium	1.13 (0.65,1.95)	0.67	147	227	2	65	0.09
		Not applicable	0.84 (0.34,2.06)	0.70	49	88	1	NA	NA
		Polymyxin B	0.81 (0.33,1.99)	0.65	40	76	1	NA	NA
	IV + IH versus IV	Colistimethate sodium	1.65 (1.06,2.57)	0.03	192	411	4	70	0.02
		Not applicable	3.03 (1.47,6.24)	< 0.01	162	205	2	0	0.43
		Polymyxin B	2.07 (1.20,2.71)	< 0.01	151	250	3	42	0.18
Microbial eradication rate	IV + IH versus IV	Colistimethate sodium	2.65 (1.56,4.48)	< 0.01	141	316	3	0	0.61
		Not applicable	10.00 (1.52,65.68)	0.02	8	29	1	NA	NA
		Polymyxin B	2.61 (1.60,4.24)	< 0.01	211	371	4	0	0.59
Acute kidney injury	IH versus IV	Colistimethate sodium	0.29 (0.16,0.71)	< 0.01	69	227	2	0	0.57
		Not applicable	0.19 (0.07,0.50)	< 0.01	32	88	1	NA	NA
		Polymyxin B	0.21 (0.07,0.62)	< 0.01	27	63	1	NA	NA
	IV + IH versus IV	Colistimethate sodium	1.12 (0.68,1.84)	0.64	130	318	3	64	0.06
		Not applicable	0.87 (0.40,1.89)	0.72	40	184	3	0	0.88
		Polymyxin B	0.66 (0.32,1.34)	0.25	48	362	4	0	0.61
Incidence of bronchospasm	IH/IV + IH versus IV	Colistimethate sodium	5.35 (0.25,113.35)	0.28	2	149	1	NA	NA
		Not applicable	9.40 (0.35,256.00)	0.18	1	31	1	NA	NA
		Polymyxin B	12.78 (1.58,103.18)	0.02	8	253	2	0	0.69
*Subgroup analysis (pathogen species)*									
Overall mortality	IV + IH versus IV	baumannii + P. aeruginosa + Enterobacteriaceae	0.68 (0.49,0.95)	0.02	342	723	7	0	0.96
		*K. pneumoniae*	0.45 (0.14,1.44)	0.18	14	121	1	NA	NA
		A. baumannii	0.50 (0.24,1.07)	0.08	56	114	2	83	0.02
Clinical success	IV + IH versus IV	A. baumannii + P. aeruginosa + Enterobacteriaceae	2.12 (1.53,2.94)	< 0.01	421	737	7	14	0.32
		A. baumannii	0.27 (0.06,1.15)	0.08	10	45	1	NA	NA
		A. baumannii + P. aeruginosa + Escherichia coli + *S. aureus*	5.25 (1.04,26.43)	0.04	74	84	1	NA	NA
Microbial eradication rate	IV + IH versus IV	A. baumannii + P. aeruginosa + Enterobacteriaceae	2.65 (1.80,3.91)	< 0.01	228	550	6	0	0.63
		*K. pneumoniae*	5.02 (1.71,14.69)	< 0.01	99	121	1	NA	NA
		A.baumannii	1.43 (0.37,5.55)	0.61	33	45	1	NA	NA

IV + IH, intravenous plus inhaled polymyxins; IV, intravenous polymyxins; IH, inhaled polymyxins; CI, confidence interval; OR, odds ratio; RCT, randomized controlled trial; NA, not available. A. baumannii, Acinetobacter baumannii; P. aeruginosa, Pseudomonas aeruginosa; *S. aureus*, *Staphylococcus aureus*; *K. pneumoniae*, *Klebsiella pneumoniae*

### Bayesian network *meta*-analysis

#### Primary outcome

**Fig. 5:**
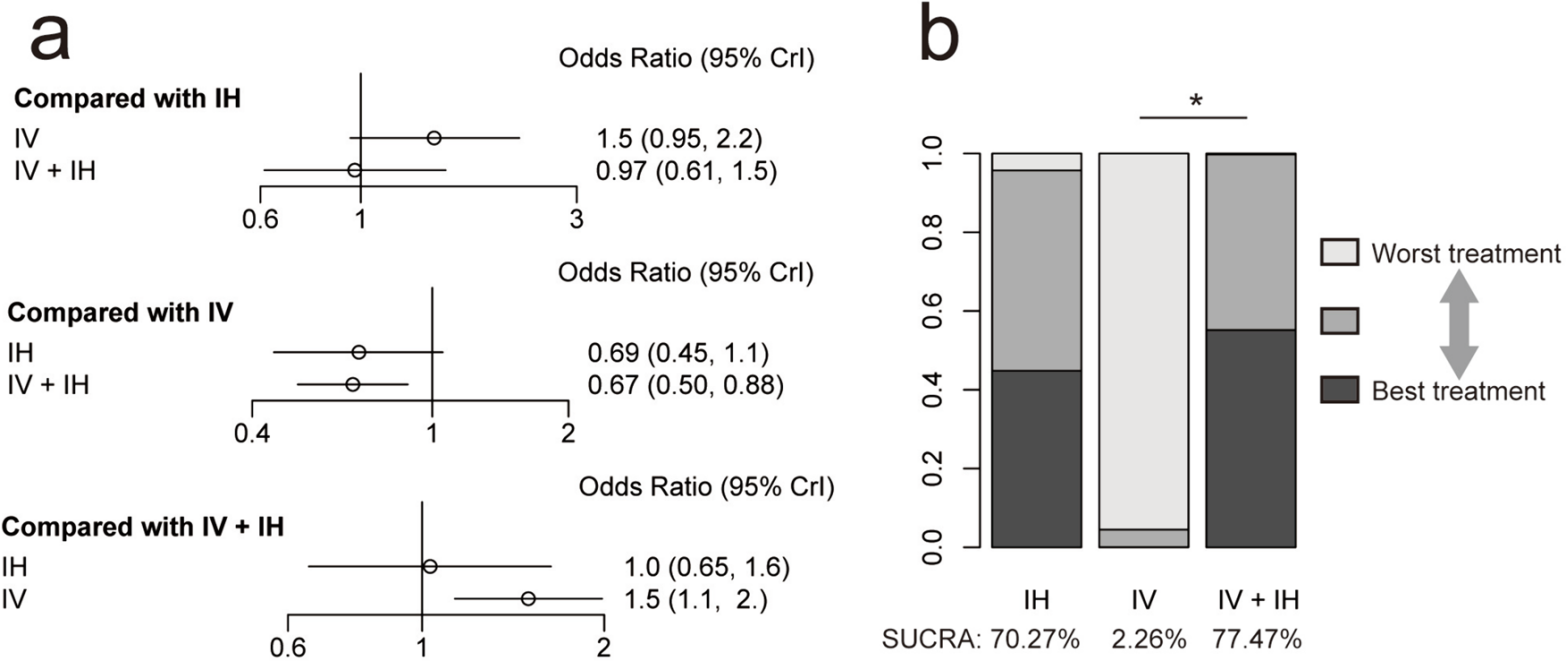
**a** Network estimates for overall mortality among polymyxin-containing regimens. **b** Rank probabilities for overall mortality among polymyxin-containing regimens. IV + IH, intravenous plus inhaled polymyxins; IV, intravenous polymyxins; IH, inhaled polymyxins. *P < 0.05

In individual comparisons for overall mortality, the network meta-analysis revealed that the IV + IH polymyxin-containing regimen significantly reduced the overall mortality compared with the IV polymyxin-containing regimen (OR 0.67; 95% CI 0.50–0.88). However, there were no significant differences between the comparisons of IV and IH polymyxin-containing regimens as well as between IH and IV + IH polymyxin-containing regimens. Ranking the administration routes on the basis of their SUCRA values indicated that the IV + IH polymyxin-containing regimen was the most effective in reducing overall mortality (SUCRA, 77.47%), followed by the IH (SUCRA, 70.27%) and IV (SUCRA, 2.26%) polymyxin-containing regimens (Fig. 5).

#### Secondary outcomes

**Fig. 6:**
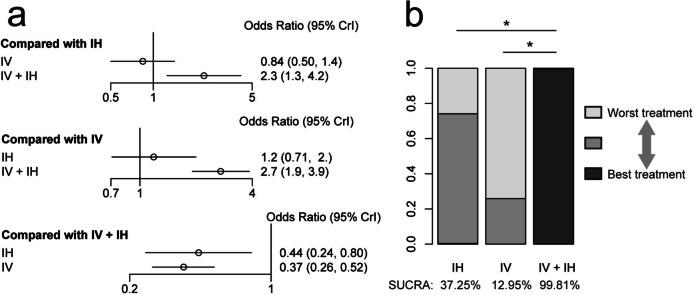
**a** Network estimates for the microbial eradication rate among polymyxin-containing regimens. **b** Rank probabilities for the microbial eradication rate among polymyxin-containing regimens. IV + IH, intravenous plus inhaled polymyxins; IV, intravenous polymyxins; IH, inhaled polymyxins. *P < 0.05

Regarding microbial eradication rates, the IV + IH polymyxin-containing regimen exhibited a higher microbial eradication rate than the IV and IH polymyxin-containing regimens (OR 2.70; 95% CI 1.90–3.90; OR 2.30; 95% CI 1.30–4.20). There were no significant differences between the IV and IH polymyxin-containing regimens. Additionally, the ranking analysis indicated that the IV + IH polymyxin-containing regimen demonstrated the highest microbial eradication rate (SUCRA, 99.81%), followed by the IH (SUCRA, 37.25%) and IV (SUCRA, 12.95%) polymyxin-containing regimens (Fig. 6).

**Fig. 7:**
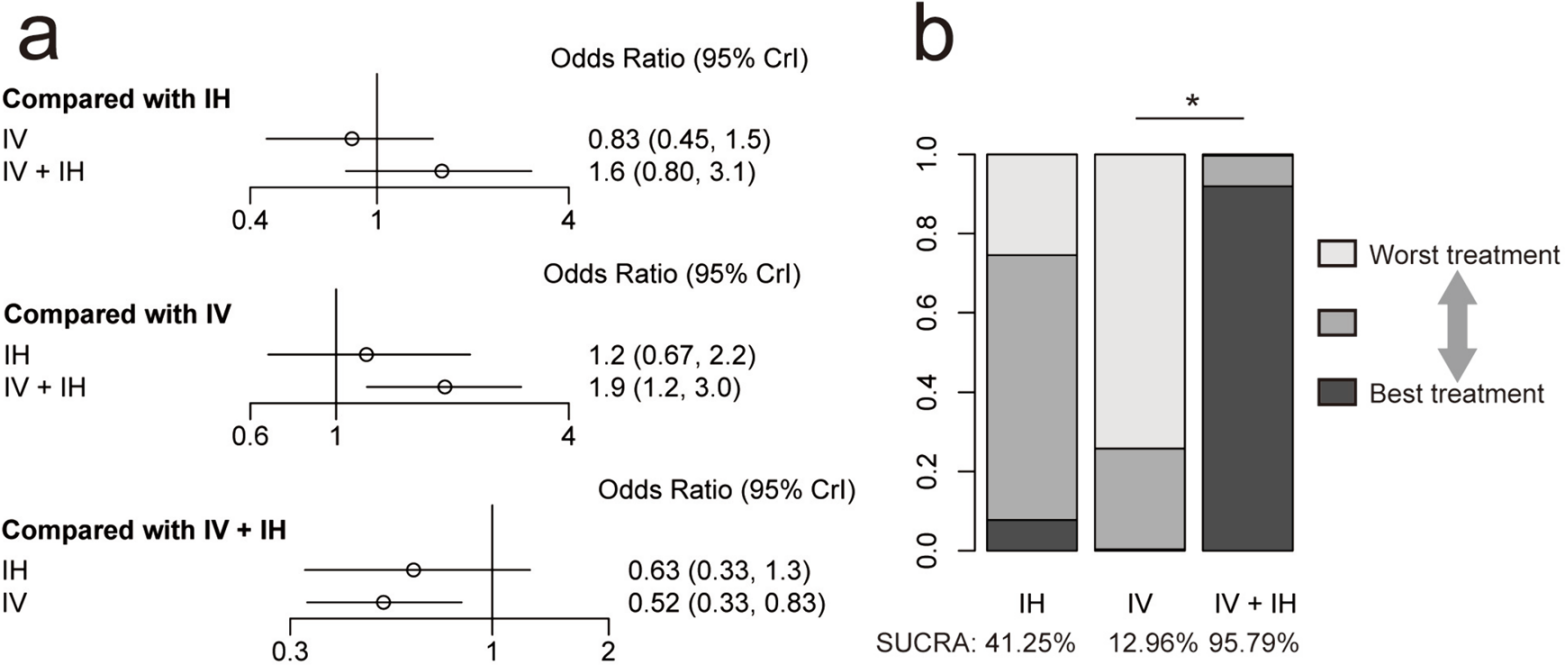
**a** Network estimates for the clinical success among polymyxin-containing regimens. **b** Rank probabilities for the clinical success among polymyxin-containing regimens. IV + IH, intravenous plus inhaled polymyxins; IV, intravenous polymyxins; IH, inhaled polymyxins. *P < 0.05

Regarding clinical success, the results indicated that the IV + IH polymyxin-containing regimen was significantly more effective than the IV polymyxin-containing regimen. The other two comparisons did not show significant differences. The ranking of SUCRA values from the highest to the lowest was IV + IH (SUCRA, 95.79%), IH (SUCRA, 41.25%), and IV (SUCRA, 12.96%) polymyxin-containing regimens (Fig. 7).

**Fig. 8:**
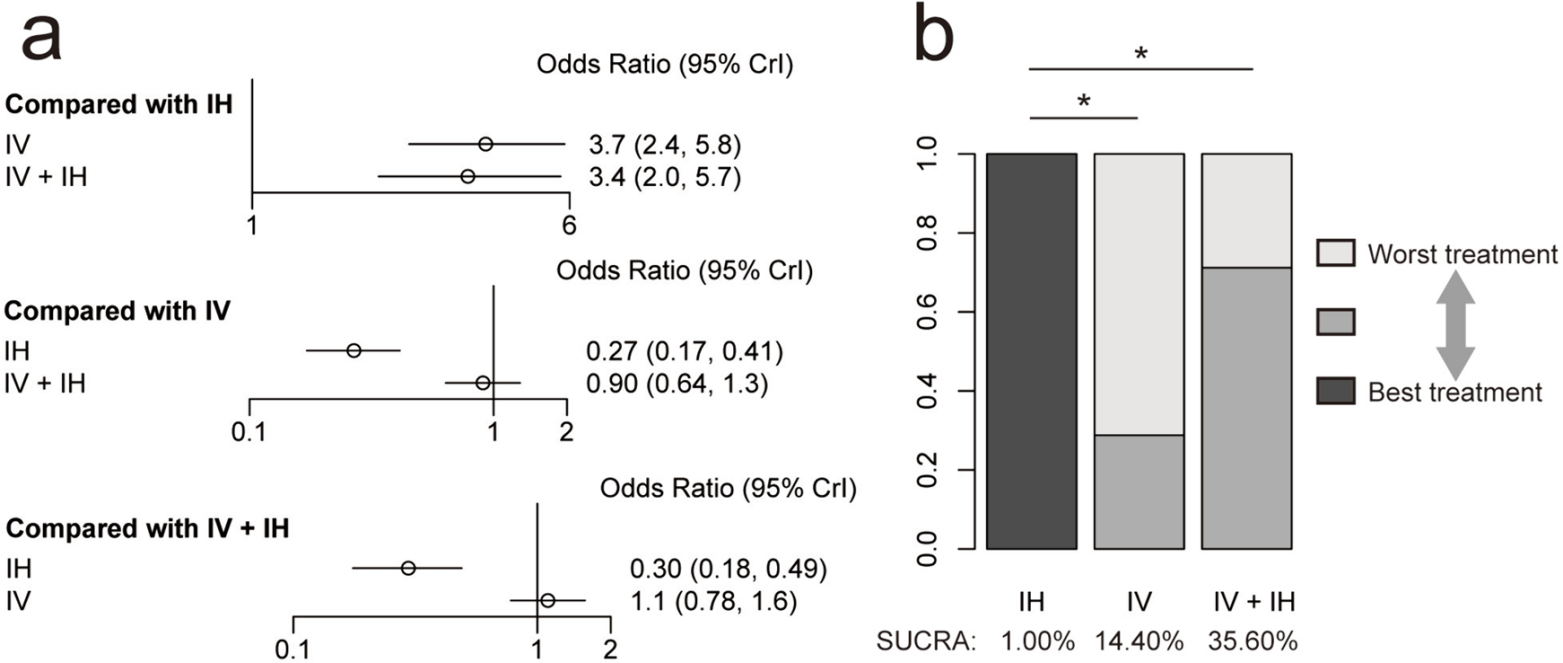
**a** Network estimates for the acute kidney injury among polymyxin-containing regimens. **b** Rank probabilities for the acute kidney injury among polymyxin-containing regimens. IV + IH, intravenous plus inhaled polymyxins; IV, intravenous polymyxins; IH, inhaled polymyxins. *P < 0.05

Regarding acute kidney injury, the findings suggested that the IH polymyxin-containing regimen significantly decreased the incidence of acute kidney injury compared with the other two administration routes. No significant differences were observed between the comparisons of IV and IV + IH polymyxin-containing regimens. Moreover, the ranking analysis reflected that the IH polymyxin-containing regimen achieved the highest ranking (SUCRA, 100.00%), followed by the IV + IH (SUCRA, 35.60%) and IV (SUCRA, 14.40%) polymyxin-containing regimens (Fig. 8).

Considering that only 4 studies reported the bronchospasm incidence and none of the IV polymyxin-containing regimen groups developed bronchospasm, no further network meta-analysis was conducted for bronchospasm incidence.

**Fig. 9:**
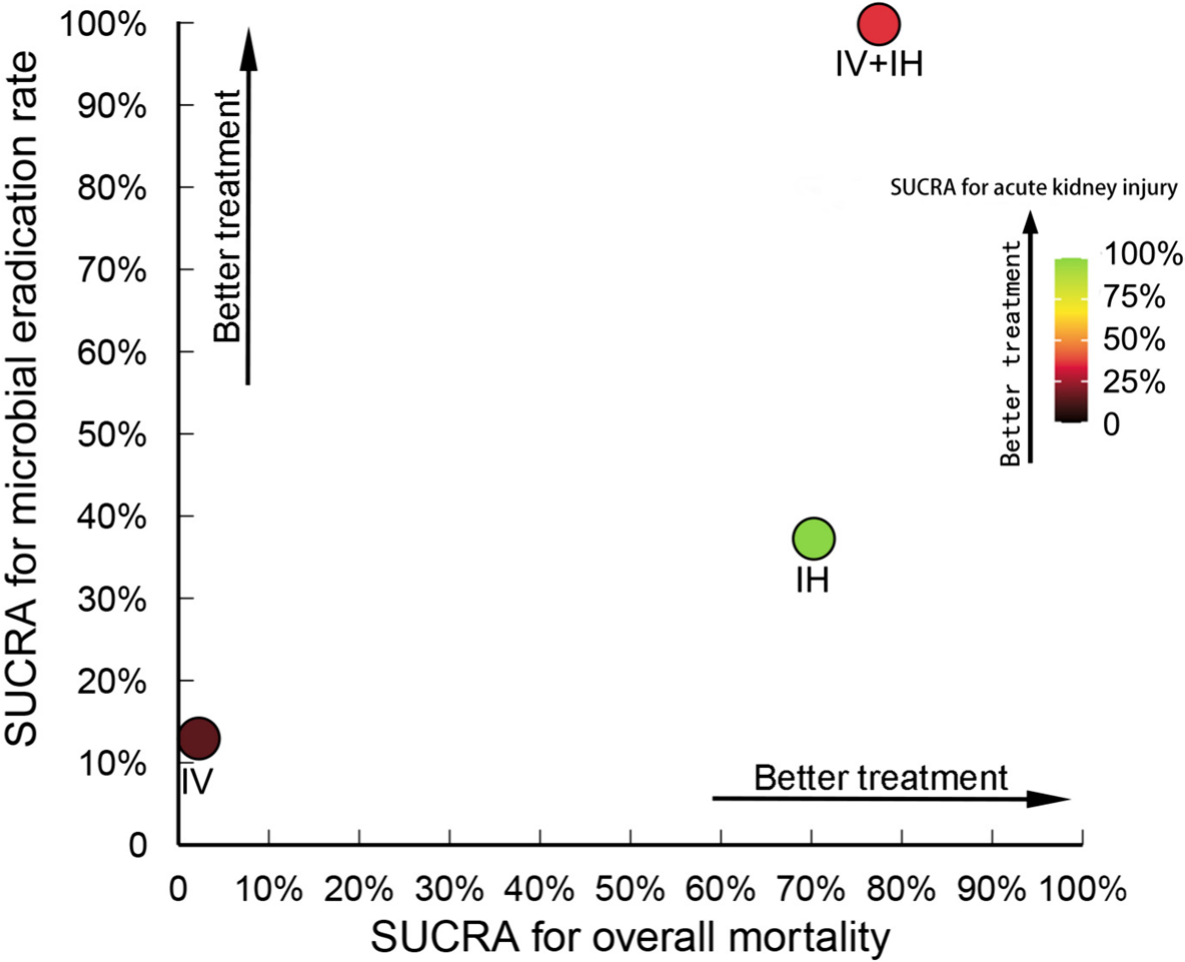
Clustered ranking plot based on the SUCRA. The plot displays the SUCRA values of the three polymyxin-containing regimens, including three outcomes: overall mortality, microbial eradication rate, and acute kidney injury. IV + IH, intravenous plus inhaled polymyxins; IV, intravenous polymyxins; IH, inhaled polymyxins

Figure 9 illustrates the clustered SUCRA ranking plot, showing three dimensions: overall mortality on the x-axis, microbial eradication rate on the y-axis, and acute kidney injury represented by bubble color. Among the three treatments, the IV + IH polymyxin-containing regimen occupies the farthest-right upper position, indicating its association with the highest microbial eradication rate and the lowest overall mortality. The IV + IH and IV polymyxin-containing regimens are shown as red and brown bubbles in the plot, indicating relatively higher acute kidney injury rates. Conversely, the IH polymyxin-containing regimen appears as a green bubble, signifying that the incidence of acute kidney injury of the IH polymyxin-containing regimen is the lowest among the three treatments. The assessment of convergence is presented in Appendix 5, Additional file 2. The evaluation of heterogeneity, consistency, and model fit is presented in Appendix 6, Additional file 2. Additionally, the findings of the publication bias analysis are presented in Appendix 7, Additional file 2.

### Credibility of evidence

We independently analyzed the evidence from RCTs and observational studies using the GRADE framework. Regarding overall mortality and clinical success, the credibility of the evidence for all three pairs of comparisons from observational studies or RCTs was low or very low. Regarding microbial eradication rate and acute kidney injury, we observed moderate-quality evidence for the microbial eradication rate for IV + IH versus IV polymyxin-containing regimens from the RCTs and high-quality evidence for the acute kidney injury for IV versus IH polymyxin-containing regimens from the RCTs; however, they were all only from a small sample in the comparisons. Therefore, further research is required to evaluate the evidence of RCTs pertaining to them. Moreover, the certainty of evidence of the acute kidney injury for IV versus IH polymyxin-containing regimens from observational studies was moderate owing to the large effect and lack of other downgrading evidence. Further, other comparisons in microbial eradication rate and acute kidney injury had very low certainty of evidence (Appendix 8, Additional file 2).

## Discussion

This study systematically analyzed three different administration regimens of polymyxins. The current findings suggest that among the three regimens, the IV + IH polymyxin-containing regimen is the most effective treatment for MDR-GNB pneumonia in terms of overall mortality, microbial eradication rate, and clinical success. Compared with the IV polymyxin-containing regimen, the IH polymyxin-containing regimen showed no significant differences in overall mortality, microbial eradication rate, and clinical success. Regarding safety, network meta-analysis and ranking analysis demonstrated that the IH polymyxin-containing regimen significantly reduced acute kidney injury when compared to the IV + IH and IV polymyxin-containing regimens. Regarding the incidence of bronchospasm, pairwise meta-analysis results suggested that the IH and IV + IH polymyxin-containing regimens can increase the incidence of bronchospasm. The results of all sensitivity analyses and most subgroup analyses were consistent with those of the main analysis. The inconsistent results without statistical differences of a few subgroup analyses were primarily attributed to the inadequate statistical power stemming from a limited number of included studies and a small sample size.

Currently, the common clinical approach for GNB pneumonia involves IV polymyxins combined with IV administration of other antibiotics, including tigecycline, carbapenems, and aminoglycosides [22, 46]. However, due to the rising antibiotic resistance, MDR-GNB has become a major pathogen in pneumonia, challenging conventional treatment strategies [47]. Consequently, IH polymyxins have recently gained increasing attention, which include adjuvant therapy (IV + IH polymyxin-containing regimen) and alternative therapy (IH polymyxin-containing regimen without IV polymyxin) [25, 43–45]. IH polymyxins offer higher concentrations at the lung epithelial surface while reducing systemic toxicity. Furthermore, the study has shown that compared to intravenous antibiotic administration, inhaled antibiotics can reduce the emergence of bacterial resistance [48]. Therefore, IH polymyxins is promising in the treatment of pulmonary infections caused by MDR-GNB. The European Society of Clinical Microbiology and Infectious Diseases suggested that more studies on IH polymyxins as an alternative therapy are urgently needed in the future [16]. To date, there has been no meta-analysis focusing on IH polymyxins as an alternative therapy (without IV polymyxin). It remains unclear whether adjunctive IV + IH polymyxins, alternative IH polymyxins, or conventional IV polymyxins provide the best efficacy. This study thoroughly assessed the efficacy and safety of these three administration regimens, providing additional evidence-based medical guidance for the use of polymyxins in MDR-GNB pneumonia.

Our network meta-analysis found that compared to IV polymyxins, IH polymyxins reduced mortality among pneumonia patients with MDR-GNB infections, although this reduction did not reach statistical significance. Importantly, IH polymyxins demonstrated the lowest incidence of acute kidney injury among the three administration methods, with statistically significant differences observed, which may be related to the lower systemic concentration of polymyxins. Existing research has suggested that the nephrotoxicity of polymyxin is related to the accumulation of high drug concentrations during renal tubular reabsorption [49]. Since its local application in the lung, IH polymyxins can prevent a large amount of drugs from entering the blood circulation. Therefore, our findings suggest that for patients with MDR-GNB pneumonia, compared with the IV polymyxin-containing regimen, using the IH regimen may be better. The reason for this is that although there is no significantly lower mortality, the IH regimen with a significantly lower incidence of acute kidney injury may be a better choice. Although few studies have documented mild-to-moderate bronchospasm in patients receiving IH polymyxin therapy, bronchospasm incidence in the IH and IV + IH polymyxin-containing regimen groups was 11 of 189 participants in the included studies [17, 18, 37, 43]. This side effect may be caused by chemical irritation of the airways and histamine release [50]. However, it can be alleviated by bronchodilators [17]. Furthermore, the optimal dose of IH polymyxin-containing regimen and the appropriate device for inhalation delivery remain to be further explored.

Regarding the comparison between IV and IV + IH polymyxin-containing regimens, previous meta-analyses have reported inconsistent results about overall mortality and clinical success. Two previous meta-analyses have indicated that the IV + IH polymyxin-containing regimen resulted in lower overall mortality and higher clinical success rate than the IV polymyxin-containing regimen [51, 52]. However, another two meta-analyses reported no significant differences in overall mortality and clinical success [53, 54]. Regarding microbial eradication rate and acute kidney injury, previous meta-analyses have shown that the IV + IH polymyxin-containing regimen exhibited a higher microbial eradication rate than the IV polymyxin-containing regimen, whereas no significant difference was found in the acute kidney injury between the two regimens [51–54]. The above published meta-analyses have some limitations. The study of Liu et al. and Valachis et al. was published in 2015 and needs to be updated by incorporating the latest researches [52, 54]. For Lu et al.’s study, only three databases were retrieved, and the included studies were all observational studies [51]. In the meta-analysis of Zhang et al., only 7 studies were controlled with intravenous polymyxins and the rest were treated with other antibiotics (determined by doctors). The inconsistent control groups may make it difficult to accurately compare the efficacy of IV polymyxins and IV + IH polymyxins [53]. Besides, two newly published studies with a sample size of 132 and 111 patients have not been included in previous analyses [43, 45]. Therefore, it is necessary to incorporate the new studies and conduct an updated meta-analysis using higher-quality methods to compare the efficacy and safety of the IH + IV regimen and the IV regimen. Therefore, we conducted a more comprehensive literature search, including nine databases. Considering the possibility of bias in low-quality studies that could not be accurately assessed, we excluded studies with a high risk of bias from the main meta-analysis. Three prospective studies (2 RCTs and 1 prospective cohort study) were included in the meta-analysis. It is worth noting that for most of the outcomes, the heterogeneity of our pairwise meta-analysis results was low, as shown in Table 3. Both the pairwise and network meta-analyses found that the IV + IH polymyxin-containing regimen is more beneficial in reducing overall mortality, improving clinical efficacy, and increasing the microbial eradication rate. This may be related to the drug concentration at the infection site. The pulmonary concentration of IV polymyxins is limited by the polycationic/hydrophilic structure of polymyxins, thereby hindering their penetration into the lung parenchyma [12]. The IV + IH polymyxin-containing regimen can simultaneously increase the drug concentration in the bloodstream and alveoli, thereby achieving a better therapeutic effect.

As the first network meta-analysis that comprehensively compared the efficacy and safety of three polymyxin-containing regimens, this study provided rigorous evidence-based insights into the clinical application of IH polymyxins as an alternative therapy (without IV polymyxins). Furthermore, by incorporating newly published literature and employing more stringent methodologies, our study addresses the inconsistent conclusions of previous meta-analyses concerning the IH + IV regimen versus the IV regimen. However, this study had some limitations. First, although we searched nine databases and conducted a rigorous literature screening process, only three RCTs were included, and most of the studies included in the analysis were retrospective cohort studies. Second, considering that patients with MDR-GNB pneumonia are often infected with complex pathogens, they frequently require treatment with a combination of multiple antibiotics. The specific antibiotic combination regimen for each individual was not provided in the original study and was not available to us. Therefore, this study could only compare IH, IV, and IV + IH polymyxin-containing regimens. Third, due to limitations in the number of studies and sample sizes, we are currently unable to precisely determine which nebulizer is most effective for aerosolizing polymyxins to achieve optimal treatment outcomes.

## Conclusion

Our study indicates that among the three administration regimens, the IV + IH polymyxin-containing regimen may be the most effective for treating MDR-GNB pneumonia, with a significantly lower overall mortality compared to the IV regimen and a considerably higher microbial eradication rate compared to the IH regimen. The IH regimen may be considered superior to the IV regimen due to its substantially lower incidence of acute kidney injury, even though the reduction in overall mortality was not significant. In the future, higher-quality non-inferiority trials are needed to compare the efficacy of IH and IV polymyxin-containing regimens.

## Supplementary Information

Additional file 1.

Additional file 2.

## Data Availability

All data and materials related to our study are available by contacting the corresponding author.

## Abbreviations

CI: Confidence interval
GRADE: Grading of recommendations assessment, development, and evaluation
ICU: Intensive care unit
IH: Inhaled
IV: Intravenous
I^2^: Inconsistency index
MDR-GNB: Multidrug-resistant gram-negative bacterial
OR: Odds ratio
RCTs: Randomized controlled trials
SUCRA: Surface under the cumulative ranking curve

## References

[1] Quartin AA, Scerpella EG, Puttagunta S, Kett DH. A comparison of microbiology and demographics among patients with healthcare-associated, hospital-acquired, and ventilator-associated pneumonia: a retrospective analysis of 1184 patients from a large, international study. BMC Infect Dis. 2013;13:561. 10.1186/1471-2334-13-561. 24279701 10.1186/1471-2334-13-561 PMC4222644

[2] Golia S, Sangeetha KT, Vasudha CL. Microbial profile of early and late onset ventilator associated pneumonia in the intensive care unit of a tertiary care hospital in Bangalore, India. J Clin Diagn Res. 2013;7(11):2462–6. 10.7860/jcdr/2013/6344.3580. 24392373 10.7860/JCDR/2013/6344.3580 PMC3879896

[3] Torres A, Aznar R, Gatell JM, Jiménez P, González J, Ferrer A, et al. Incidence, risk, and prognosis factors of nosocomial pneumonia in mechanically ventilated patients. Am Rev Respir Dis. 1990;142(3):523–8. 10.1164/ajrccm/142.3.523. 2202245 10.1164/ajrccm/142.3.523

[4] Khan HA, Baig FK, Mehboob R. Nosocomial infections: epidemiology, prevention, control and surveillance. Asian Pac J Trop Biomed. 2017;7(5):478–82. 10.1016/j.apjtb.2017.01.019.

[5] Troeger C, Blacker B, Khalil IA, Rao PC, Cao J, Zimsen SR, Albertson SB, Deshpande A, Farag T, Abebe Z, Adetifa IM. Estimates of the global, regional, and national morbidity, mortality, and aetiologies of lower respiratory infections in 195 countries, 1990–2016: a systematic analysis for the Global Burden of Disease Study 2016. Lancet Infect Dis. 2018;18(11):1191–210. 10.1016/s1473-3099(18)30310-4. 30243584 10.1016/S1473-3099(18)30310-4 PMC6202443

[6] Ben Lakhal H, M’Rad A, Naas T, Brahmi N. Antimicrobial susceptibility among pathogens isolated in early- versus late-onset ventilator-associated pneumonia. Infect Dis Rep. 2021;13(2):401–10. 10.3390/idr13020038. 33925385 10.3390/idr13020038 PMC8167786

[7] Chang Y, Jeon K, Lee SM, Cho YJ, Kim YS, Chong YP, et al. The distribution of multidrug-resistant microorganisms and treatment status of hospital-acquired pneumonia/ventilator-associated pneumonia in adult intensive care units: a prospective cohort observational study. J Korean Med Sci. 2021;36(41):e251. 10.3346/jkms.2021.36.e251. 34697926 10.3346/jkms.2021.36.e251 PMC8546312

[8] El-Sayed Ahmed MAE, Zhong LL, Shen C, Yang Y, Doi Y, Tian GB. Colistin and its role in the era of antibiotic resistance: an extended review (2000–2019). Emerg Microbes Infect. 2020;9(1):868–85. 10.1080/22221751.2020.1754133. 32284036 10.1080/22221751.2020.1754133 PMC7241451

[9] Li J, Nation RL, Turnidge JD, Milne RW, Coulthard K, Rayner CR, et al. Colistin: the re-emerging antibiotic for multidrug-resistant Gram-negative bacterial infections. Lancet Infect Dis. 2006;6(9):589–601. 10.1016/s1473-3099(06)70580-1. 16931410 10.1016/S1473-3099(06)70580-1

[10] Dhariwal AK, Tullu MS. Colistin: re-emergence of the “forgotten” antimicrobial agent. J Postgrad Med. 2013;59(3):208–15. 10.4103/0022-3859.118040. 24029199 10.4103/0022-3859.118040

[11] Wagenlehner F, Lucenteforte E, Pea F, Soriano A, Tavoschi L, Steele VR, et al. Systematic review on estimated rates of nephrotoxicity and neurotoxicity in patients treated with polymyxins. Clin Microbiol Infect. 2021. 10.1016/j.cmi.2020.12.009. 33359542 10.1016/j.cmi.2020.12.009

[12] Imberti R, Cusato M, Villani P, Carnevale L, Iotti GA, Langer M, et al. Steady-state pharmacokinetics and BAL concentration of colistin in critically ill patients after IV colistin methanesulfonate administration. Chest. 2010;138(6):1333–9. 10.1378/chest.10-0463. 20558557 10.1378/chest.10-0463

[13] Tumbarello M, De Pascale G, Trecarichi EM, De Martino S, Bello G, Maviglia R, et al. Effect of aerosolized colistin as adjunctive treatment on the outcomes of microbiologically documented ventilator-associated pneumonia caused by colistin-only susceptible gram-negative bacteria. Chest. 2013;144(6):1768–75. 10.1378/chest.13-1018. 23989805 10.1378/chest.13-1018

[14] Korbila IP, Michalopoulos A, Rafailidis PI, Nikita D, Samonis G, Falagas ME. Inhaled colistin as adjunctive therapy to intravenous colistin for the treatment of microbiologically documented ventilator-associated pneumonia: a comparative cohort study. Clin Microbiol Infect. 2010;16(8):1230–6. 10.1111/j.1469-0691.2009.03040.x. 19732088 10.1111/j.1469-0691.2009.03040.x

[15] Tsuji BT, Pogue JM, Zavascki AP, Paul M, Daikos GL, Forrest A, et al. International consensus guidelines for the optimal use of the polymyxins: endorsed by the American College of Clinical Pharmacy (ACCP), European Society of Clinical Microbiology and Infectious Diseases (ESCMID), Infectious Diseases Society of America (IDSA), International Society for Anti-infective Pharmacology (ISAP), Society of Critical Care Medicine (SCCM), and Society of Infectious Diseases Pharmacists (SIDP). Pharmacotherapy. 2019;39(1):10–39. 10.1002/phar.2209. 30710469 10.1002/phar.2209 PMC7437259

[16] Rello J, Solé-Lleonart C, Rouby JJ, Chastre J, Blot S, Poulakou G, et al. Use of nebulized antimicrobials for the treatment of respiratory infections in invasively mechanically ventilated adults: a position paper from the European Society of Clinical Microbiology and Infectious Diseases. Clin Microbiol Infect. 2017;23(9):629–39. 10.1016/j.cmi.2017.04.011. 28412382 10.1016/j.cmi.2017.04.011

[17] Abdellatif S, Trifi A, Daly F, Mahjoub K, Nasri R, Ben LS. Efficacy and toxicity of aerosolised colistin in ventilator-associated pneumonia: a prospective, randomised trial. Ann Intensive Care. 2016;6(1):26. 10.1186/s13613-016-0127-7. 27033711 10.1186/s13613-016-0127-7 PMC4816935

[18] Hasan MJ, Rabbani R, Anam AM, Santini A, Huq SMR. The SUSCEPTIBILITY of MDR-K. pneumoniae to polymyxin B plus its nebulised form versus polymyxin B alone in critically ill south Asian patients. J Crit Care Med (Targu Mures). 2021;7(1):28–36. 10.2478/jccm-2020-0044. 34722901 10.2478/jccm-2020-0044 PMC8519379

[19] Rouby JJ, Sole-Lleonart C, Rello J. Ventilator-associated pneumonia caused by multidrug-resistant Gram-negative bacteria: understanding nebulization of aminoglycosides and colistin. Intensive Care Med. 2020;46(4):766–70. 10.1007/s00134-019-05890-w. 31915838 10.1007/s00134-019-05890-w PMC7223812

[20] Hutton B, Salanti G, Caldwell DM, Chaimani A, Schmid CH, Cameron C, et al. The PRISMA extension statement for reporting of systematic reviews incorporating network meta-analyses of health care interventions: checklist and explanations. Ann Intern Med. 2015;162(11):777–84. 10.7326/m14-2385. 26030634 10.7326/M14-2385

[21] Magiorakos AP, Srinivasan A, Carey RB, Carmeli Y, Falagas ME, Giske CG, et al. Multidrug-resistant, extensively drug-resistant and pandrug-resistant bacteria: an international expert proposal for interim standard definitions for acquired resistance. Clin Microbiol Infect. 2012;18(3):268–81. 10.1111/j.1469-0691.2011.03570.x. 21793988 10.1111/j.1469-0691.2011.03570.x

[22] Almangour TA, Alruwaili A, Almutairi R, Alrasheed A, Alhifany AA, Eljaaly K, et al. Aerosolized plus intravenous colistin vs intravenous colistin alone for the treatment of nosocomial pneumonia due to multidrug-resistant Gram-negative bacteria: a retrospective cohort study. Int J Infect Dis. 2021;108:406–12. 10.1016/j.ijid.2021.06.007. 34111542 10.1016/j.ijid.2021.06.007

[23] Zheng JY, Huang SS, Huang SH, Ye JJ. Colistin for pneumonia involving multidrug-resistant Acinetobacter calcoaceticus-Acinetobacter baumannii complex. J Microbiol Immunol Infect. 2020;53(6):854–65. 10.1016/j.jmii.2019.08.007. 31607573 10.1016/j.jmii.2019.08.007

[24] Kellum JA, Lameire N. Diagnosis, evaluation, and management of acute kidney injury: a KDIGO summary (part 1). Crit Care. 2013;17(1):204. 10.1186/cc11454. 23394211 10.1186/cc11454 PMC4057151

[25] Wu Z, Zhang S, Cao Y, Wang Q, Sun K, Zheng X. Comparison of the clinical efficacy and toxicity of nebulized polymyxin monotherapy and combined intravenous and nebulized polymyxin for the treatment of ventilator-associated pneumonia caused by carbapenem-resistant gram-negative bacteria: a retrospective cohort study. Front Pharmacol. 2023;14:1209063. 10.3389/fphar.2023.1209063. 37663252 10.3389/fphar.2023.1209063 PMC10470629

[26] Higgins JP, Altman DG, Gøtzsche PC, Jüni P, Moher D, Oxman AD, et al. The Cochrane collaboration’s tool for assessing risk of bias in randomised trials. BMJ. 2011;343:d5928. 10.1136/bmj.d5928. 22008217 10.1136/bmj.d5928 PMC3196245

[27] Sterne JA, Hernán MA, Reeves BC, Savović J, Berkman ND, Viswanathan M, et al. ROBINS-I: a tool for assessing risk of bias in non-randomised studies of interventions. BMJ. 2016;355:i4919. 10.1136/bmj.i4919. 27733354 10.1136/bmj.i4919 PMC5062054

[28] Jakobsen JC, Wetterslev J, Winkel P, Lange T, Gluud C. Thresholds for statistical and clinical significance in systematic reviews with meta-analytic methods. BMC Med Res Methodol. 2014;14:120. 10.1186/1471-2288-14-120. 25416419 10.1186/1471-2288-14-120 PMC4251848

[29] Spiegelhalter D, Best N, Carlin B, Van Der Linde A. Bayesian measures of model complexity and fit. J R Stat Soc Ser B (Stat Methodol). 2002;64(4):583–639. 10.1111/1467-9868.00353.

[30] Salanti G, Ades AE, Ioannidis JP. Graphical methods and numerical summaries for presenting results from multiple-treatment meta-analysis: an overview and tutorial. J Clin Epidemiol. 2011;64(2):163–71. 10.1016/j.jclinepi.2010.03.016. 20688472 10.1016/j.jclinepi.2010.03.016

[31] Guyatt G, Oxman AD, Akl EA, Kunz R, Vist G, Brozek J, et al. GRADE guidelines: 1. Introduction-GRADE evidence profiles and summary of findings tables. J Clin Epidemiol. 2011;64(4):383–94. 10.1016/j.jclinepi.2010.04.026. 21195583 10.1016/j.jclinepi.2010.04.026

[32] GRADEpro GDT. https://gdt.gradepro.org/app/. Accessed 29 Dec 2023.

[33] Guyatt GH, Oxman AD, Kunz R, Brozek J, Alonso-Coello P, Rind D, et al. GRADE guidelines 6. Rating the quality of evidence–imprecision. J Clin Epidemiol. 2011;64(12):1283–93. 10.1016/j.jclinepi.2011.01.012. 21839614 10.1016/j.jclinepi.2011.01.012

[34] Schünemann H, Brożek J, Guyatt G, Oxman A. GRADE handbook. In: GRADE handbook. The Cochrane collaboration. https://gdt.gradepro.org/app/handbook/handbook.html. Accessed 23 Dec 2023.

[35] Ahn SH, Lee SJ, Ahn H-L, Hwangbo SY. Comparative evaluation of intravenous vs. nebulized colistin treatment of pneumonia due to multidrug-resistant Acinetobacter baumannii & Pseudomonas aeruginosa. J Kor Soc Health-Syst Pharm. 2020;37(1):11–9. 10.32429/jkshp.2020.37.1.001.

[36] Amin M, Rashad A, Fouad A, Abdel AA. Re-emerging of colistin for treatment of nosocomial pneumonia due to gram negative multi-drug resistant pathogens in critically ill patients. Egypt J Chest Dis Tuberc. 2013;62(3):447–51. 10.1016/j.ejcdt.2013.05.012.

[37] Bogović TZ, Budimir A, Bošnjak Z, Hrabač P, Baronica R, Tomašević B, et al. Inhalation plus intravenous colistin versus intravenous colistin alone for treatment of ventilator associated pneumonia. Signa Vitae. 2014;9(SUPPL. 1):29–33.

[38] Choe J, Sohn YM, Jeong SH, Park HJ, Na SJ, Huh K, et al. Inhalation with intravenous loading dose of colistin in critically ill patients with pneumonia caused by carbapenem-resistant gram-negative bacteria. Ther Adv Respir Dis. 2019;13:1753466619885529. 10.1177/1753466619885529. 31680646 10.1177/1753466619885529 PMC6852352

[39] Kalin G, Alp E, Coskun R, Demiraslan H, Gundogan K, Doganay M. Use of high-dose IV and aerosolized colistin for the treatment of multidrug-resistant Acinetobacter baumannii ventilator-associated pneumonia: do we really need this treatment? J Infect Chemother. 2012;18(6):872–7. 10.1007/s10156-012-0430-7. 22644081 10.1007/s10156-012-0430-7

[40] Kim YK, Lee JH, Lee HK, Chung BC, Yu SJ, Lee HY, et al. Efficacy of nebulized colistin-based therapy without concurrent intravenous colistin for ventilator-associated pneumonia caused by carbapenem-resistant Acinetobacter baumannii. J Thorac Dis. 2017;9(3):555–67. 10.21037/jtd.2017.02.61. 28449463 10.21037/jtd.2017.02.61 PMC5394082

[41] Zhou L, Li C, Weng Q, Wu J, Luo H, Xue Z, et al. Clinical study on intravenous combined with aerosol inhalation of polymyxin B for the treatment of pneumonia caused by multidrug-resistant Gram-negative bacteria. Chin Crit Care Med. 2021;33(4):416–20. 10.3760/cma.j.cn121430-20201215-00753. 10.3760/cma.j.cn121430-20201215-00753 34053483

[42] Lin H, Liu X, Sun P. Effects of aerosol inhalation combined with intravenous drip of polymyxin B on bacterial clearance, symptoms improvement, and serum infection indexes in patients with pneumonia induced by multidrug-resistant gram-negative bacteria. Emerg Med Int. 2022;2022:5244538. 10.1155/2022/5244538. 36072613 10.1155/2022/5244538 PMC9441374

[43] Liu J, Shao M, Xu Q, Liu F, Pan X, Wu J, et al. Low-dose intravenous plus inhaled versus intravenous polymyxin B for the treatment of extensive drug-resistant Gram-negative ventilator-associated pneumonia in the critical illnesses: a multi-center matched case-control study. Ann Intensive Care. 2022;12(1):72. 10.1186/s13613-022-01033-5. 35934730 10.1186/s13613-022-01033-5 PMC9357592

[44] Matijašević J, Gavrilović S, Andrijević I, Andrijević A, Milić S, Vukoja M. Inhalatory and intravenous colistin in treating ventilator-associated pneumonia due to Acinetobacter species: should we combine them? Vojnosanit Pregl. 2018;77(8):832–8. 10.2298/vsp180910161m.

[45] Shi R, Fu Y, Gan Y, Wu D, Zhou S, Huang M. Use of polymyxin B with different administration methods in the critically ill patients with ventilation associated pneumonia: a single-center experience. Front Pharmacol. 2023;14:1222044. 10.3389/fphar.2023.1222044. 37719858 10.3389/fphar.2023.1222044 PMC10502420

[46] Kalil AC, Metersky ML, Klompas M, Muscedere J, Sweeney DA, Palmer LB, et al. Management of adults with hospital-acquired and ventilator-associated pneumonia: 2016 clinical practice guidelines by the infectious diseases Society of America and the American Thoracic Society. Clin Infect Dis. 2016;63(5):e61–111. 10.1093/cid/ciw353. 27418577 10.1093/cid/ciw353 PMC4981759

[47] Sader HS, Farrell DJ, Flamm RK, Jones RN. Antimicrobial susceptibility of Gram-negative organisms isolated from patients hospitalised with pneumonia in US and European hospitals: results from the SENTRY Antimicrobial Surveillance Program, 2009–2012. Int J Antimicrob Agents. 2014;43(4):328–34. 10.1016/j.ijantimicag.2014.01.007. 24630306 10.1016/j.ijantimicag.2014.01.007

[48] Lu Q, Yang J, Liu Z, Gutierrez C, Aymard G, Rouby JJ. Nebulized ceftazidime and amikacin in ventilator-associated pneumonia caused by Pseudomonas aeruginosa. Am J Respir Crit Care Med. 2011;184(1):106–15. 10.1164/rccm.201011-1894OC. 21474643 10.1164/rccm.201011-1894OC

[49] Tran TB, Velkov T, Nation RL, Forrest A, Tsuji BT, Bergen PJ, et al. Pharmacokinetics/pharmacodynamics of colistin and polymyxin B: are we there yet? Int J Antimicrob Agents. 2016;48(6):592–7. 10.1016/j.ijantimicag.2016.09.010. 27793510 10.1016/j.ijantimicag.2016.09.010 PMC5154767

[50] Alothman GA, Ho B, Alsaadi MM, Ho SL, O’Drowsky L, Louca E, et al. Bronchial constriction and inhaled colistin in cystic fibrosis. Chest. 2005;127(2):522–9. 10.1378/chest.127.2.522. 15705991 10.1378/chest.127.2.522

[51] Lu D, Mao W. Efficacy and safety of intravenous combined with aerosolised polymyxin versus intravenous polymyxin alone in the treatment of multidrug-resistant gram-negative bacterial pneumonia: a systematic review and meta-analysis. Heliyon. 2023;9(5):e15774. 10.1016/j.heliyon.2023.e15774. 37159708 10.1016/j.heliyon.2023.e15774 PMC10163663

[52] Liu D, Zhang J, Liu HX, Zhu YG, Qu JM. Intravenous combined with aerosolised polymyxin versus intravenous polymyxin alone in the treatment of pneumonia caused by multidrug-resistant pathogens: a systematic review and meta-analysis. Int J Antimicrob Agents. 2015;46(6):603–9. 10.1016/j.ijantimicag.2015.09.011. 26607337 10.1016/j.ijantimicag.2015.09.011

[53] Zhang X, Cui X, Jiang M, Huang S, Yang M. Nebulized colistin as the adjunctive treatment for ventilator-associated pneumonia: a systematic review and meta-analysis. J Crit Care. 2023;77:154315. 10.1016/j.jcrc.2023.154315. 37120926 10.1016/j.jcrc.2023.154315

[54] Valachis A, Samonis G, Kofteridis DP. The role of aerosolized colistin in the treatment of ventilator-associated pneumonia: a systematic review and metaanalysis. Crit Care Med. 2015;43(3):527–33. 10.1097/ccm.0000000000000771. 25493971 10.1097/CCM.0000000000000771

