# Supplementary material for: Efficacy and safety of different polymyxin-containing regimens for the treatment of pneumonia caused by multidrug-resistant gram-negative bacteria: a systematic review and network meta-analysis
Source: Crit Care. 2024 Jul 14;28:239. doi: 10.1186/s13054-024-05031-w (PMC11247855; doi:10.1186/s13054-024-05031-w)
Supplement: Supplementary file 2 — Additional file 2. [file 13054_2024_5031_MOESM2_ESM.docx]

**Additional File 2**

[Appendix 1: The details of the search strategy 2](#_Toc13941)

[Appendix 2: Risk of bias assessment 7](#_Toc21179)

[Appendix 3: Forest plots of pairwise meta-analysis 9](#_Toc6630)

[Appendix 4: Sensitivity analyses and subgroup analyses 12](#_Toc1309)

[Appendix 5: Convergence and density plot 28](#_Toc833)

[Appendix 6: Assessment of heterogeneity, consistency and model fit 30](#_Toc9054)

[Appendix 7: Publication bias analysis: Funnel plots and Egger' s test 32](#_Toc27942)

[Appendix 8: Certainty of evidence 39](#_Toc12822)

[Appendix 9: Definition of clinical success 43](#_Toc26011)

[Appendix 10: Definition of acute kidney injury 45](#_Toc18554)

**Appendix 1: The details of the search strategy**

| Search strategy | Result |
| --- | --- |
| PubMed |  |
| (("Infusions, Intravenous"[Mesh] OR "Administration, Intravenous"[Mesh] OR "Injections, Intravenous"[Mesh] OR (Infusions, Intravenous) OR (Administration, Intravenous) OR (Injections, Intravenous) OR Intravenous OR Parenteral OR I.V OR infusions OR infusion OR Injection OR Injections OR IV OR venous drip OR venous transfusion) OR ("Inhalation"[Mesh] OR Inhalation OR Aerosolized OR Nebulized OR inhaled OR Aerosolised OR Nebulised OR Inhaling OR (Inspiration, Respiratory) OR Respiratory Inspiration OR Spray OR Aerosol OR parenteral OR aerosolexposure OR aerosolinhalation OR aerosolen OR aerosolization OR aerosols OR dentalaerosol OR pressurizedaerosol OR nebulization OR atomization OR mist therapy OR ultrasonic atomization)) AND ("Colistin"[Mesh] OR "Polymyxin B"[Mesh] OR "Polymyxins"[Mesh] OR Colistin OR Polymyxin E OR Colimycin OR Colisticin OR Coly-Mycin OR Totazina OR colistimethate sodium OR Colistin Sulfate OR CMS OR colistin methanesulfonate OR (Sulfate, Colistin) OR Polymyxin B OR Polymyxin OR Polymyxins OR Aerosporin OR belcomycin OR colicort OR colimycine OR colistine OR colomycin OR coly mycin OR colymicin OR colymycin OR multimycin OR polimixin OR polimyxin OR polymixin OR polymycin OR polymyxine OR bacillosporin OR poly-rx) AND ("Pneumonia"[Mesh] OR Pneumonia OR Pneumonias OR Lobar Pneumonia OR Lobar Pneumonias OR (Pneumonias, Lobar) OR (Pneumonia, Lobar) OR Experimental Lung Inflammation OR Experimental Lung Inflammations OR (Inflammation, Experimental Lung) OR (Lung Inflammation, Experimental) OR (Lung Inflammations, Experimental) OR Pneumonitis OR Pneumonitides OR Pulmonary Inflammation OR (Inflammation, Pulmonary) OR (Inflammations, Pulmonary) OR Pulmonary Inflammations OR Lung Inflammation OR (Inflammation, Lung) OR (Inflammations, Lung) OR Lung Inflammations OR pneumonic OR inflammatory lung disease OR lobitis OR nonspecific inflammatory lung disease OR peripneumonia OR pleuropneumonia OR pleuropneumonitis OR pulmonal inflammation OR pulmonic inflammation)  The search was conducted from the establishment of the library to November 15, 2023, and 603 results were retrieved. | 603 |
| The Cochrane Library | |
| #1 MeSH descriptor: [Pneumonia] explode all trees | 10637 |
| #2 MeSH descriptor: [Colistin] explode all trees | 216 |
| #3 MeSH descriptor: [Polymyxin B] explode all trees | 201 |
| #4 MeSH descriptor: [Polymyxins] explode all trees | 476 |
| #5 MeSH descriptor: [Inhalation] explode all trees | 783 |
| #6 MeSH descriptor: [Infusions, Intravenous] explode all trees | 11183 |
| #7 MeSH descriptor: [Injections, Intravenous] explode all trees | 8062 |
| #8 MeSH descriptor: [Administration, Intravenous] explode all trees | 20413 |
| #9 Infusions, Intravenous OR Administration, Intravenous OR Injections, Intravenous OR Intravenous OR Parenteral OR infusions OR infusion OR Injection OR Injections OR venous drip OR venous transfusion OR Inhalation OR Aerosolized OR Nebulized OR inhaled OR Aerosolised OR Nebulised OR Inhaling OR Inspiration, Respiratory OR Respiratory Inspiration OR Spray OR Aerosol OR parenteral OR aerosolexposure OR aerosolinhalation OR aerosolen OR aerosolization OR aerosols OR dentalaerosol OR pressurizedaerosol OR nebulization OR atomization OR mist therapy OR ultrasonic atomization OR IV OR I.V | 334351 |
| #10 Pneumonia OR Pneumonias OR Lobar Pneumonia OR Lobar Pneumonias OR Pneumonias, Lobar OR Pneumonia, Lobar OR Experimental Lung Inflammation OR Experimental Lung Inflammations OR Inflammation, Experimental Lung OR Lung Inflammation, Experimental OR Lung Inflammations, Experimental OR Pneumonitis OR Pneumonitides OR Pulmonary Inflammation OR Inflammation, Pulmonary OR Inflammations, Pulmonary OR Pulmonary Inflammations OR Lung Inflammation OR Inflammation, Lung OR Inflammations, Lung OR Lung Inflammations OR pneumonic OR inflammatory lung disease OR lobitis OR nonspecific inflammatory lung disease OR peripneumonia OR pleuropneumonia OR pleuropneumonitis OR pulmonal inflammation OR pulmonic inflammation | 30109 |
| #11 Colistin OR Polymyxin E OR Colimycin OR Colisticin OR Coly-Mycin OR Totazina OR colistimethate sodium OR Colistin Sulfate OR colistin methanesulfonate OR Sulfate, Colistin OR Polymyxin B OR Polymyxin OR Polymyxins OR Aerosporin OR belcomycin OR colicort OR colimycine OR colistine OR colomycin OR coly mycin OR colymicin OR colymycin OR multimycin OR polimixin OR polimyxin OR polymixin OR polymycin OR polymyxine OR bacillosporin OR poly-rx OR CMS | 2973 |
| #12 #1 OR #10 | 34303 |
| #13 #2 OR #3 OR #4 OR #11 | 2973 |
| #14 #5 OR #6 OR #7 OR #8 OR #9 | 334351 |
| #15 #12 AND #13 AND #14  The search was conducted from the establishment of the library to November 15, 2023, and 190 results were retrieved. | 190 |
| Embase | |
| #1 'intravenous drug administration'/exp OR 'injection'/exp OR 'intravenous drug administration' OR 'injection' OR 'infusions, intravenous' OR 'administration, intravenous' OR 'injections, intravenous' OR 'intravenous' OR 'parenteral' OR 'i.v' OR 'infusions' OR 'infusion' OR 'injections' OR 'iv' OR 'venous drip' OR 'venous transfusion' | 3,137,641 |
| #2 'inhalation'/exp OR 'aerosol'/exp OR 'nebulization'/exp OR 'parenteral drug administration'/exp OR 'inhalation' OR 'aerosol' OR 'nebulization' OR 'parenteral drug administration' OR 'aerosolized' OR 'nebulized' OR 'inhaled' OR 'aerosolised' OR 'nebulised' OR 'inhaling' OR 'inspiration, respiratory' OR 'respiratory inspiration' OR 'spray' OR 'parenteral' OR 'aerosolexposure' OR 'aerosolinhalation' OR 'aerosolen' OR 'aerosolization' OR 'aerosols' OR 'dentalaerosol' OR 'pressurizedaerosol' OR 'atomization' OR 'mist therapy' OR 'ultrasonic atomization' | 1,220,053 |
| #3 'colistin'/exp OR 'polymyxin'/exp OR 'polymyxin b'/exp OR 'colistin' OR 'polymyxin' OR 'polymyxin b' OR 'polymyxin e' OR 'colimycin' OR 'colisticin' OR 'coly-mycin' OR 'totazina' OR 'colistimethate sodium' OR 'colistin sulfate' OR 'cms' OR 'colistin methanesulfonate' OR 'sulfate, colistin' OR 'polymyxins' OR 'aerosporin' OR 'belcomycin' OR 'colicort' OR 'colimycine' OR 'colistine' OR 'colomycin' OR 'coly mycin' OR 'colymicin' OR 'colymycin' OR 'multimycin' OR 'polimixin' OR 'polimyxin' OR 'polymixin' OR 'polymycin' OR 'polymyxine' OR 'bacillosporin' OR 'poly-rx' | 97,718 |
| #4 'pneumonia'/exp OR 'pneumonia' OR 'pneumonias' OR 'lobar pneumonia' OR 'lobar pneumonias' OR 'pneumonias, lobar' OR 'pneumonia, lobar' OR 'experimental lung inflammation' OR 'experimental lung inflammations' OR 'inflammation, experimental lung' OR 'lung inflammation, experimental' OR 'lung inflammations, experimental' OR 'pneumonitis' OR 'pneumonitides' OR 'pulmonary inflammation' OR 'inflammation, pulmonary' OR 'inflammations, pulmonary' OR 'pulmonary inflammations' OR 'lung inflammation' OR 'inflammation, lung' OR 'inflammations, lung' OR 'lung inflammations' OR 'pneumonic' OR 'inflammatory lung disease' OR 'lobitis' OR 'nonspecific inflammatory lung disease' OR 'peripneumonia' OR 'pleuropneumonia' OR 'pleuropneumonitis' OR 'pulmonal inflammation' OR 'pulmonic inflammation' | 485,537 |
| #5 #1 OR #2 | 3,668,471 |
| #6 #3 AND #4 AND #5  The search was conducted from the establishment of the library to November 15, 2023, and 2144 results were retrieved. | 2144 |
| Web of science | |
| ((TS=(Infusions, Intravenous) OR TS=(Administration, Intravenous) OR TS=(Injections, Intravenous) OR TS=(Intravenous) OR TS=(Parenteral) OR TS=(I.V) OR TS=(infusions) OR TS=(infusion) OR TS=(Injection) OR TS=(Injections) OR TS=(IV) OR TS=(venous drip) OR TS=(venous transfusion)) OR (TS=(Inhalation) OR TS=(Aerosolized) OR TS=(Nebulized) OR TS=(inhaled) OR TS=(Aerosolised) OR TS=(Nebulised) OR TS=(Inhaling) OR TS=(Inspiration, Respiratory) OR TS=(Respiratory Inspiration) OR TS=(Spray) OR TS=(Aerosol) OR TS=(parenteral) OR TS=(aerosolexposure) OR TS=(aerosolinhalation) OR TS=(aerosolen) OR TS=(aerosolization) OR TS=(aerosols) OR TS=(dentalaerosol) OR TS=(pressurizedaerosol) OR TS=(nebulization) OR TS=(atomization) OR TS=(mist therapy) OR TS=(ultrasonic atomization))) AND (TS=(Colistin) OR TS=(Polymyxin E) OR TS=(Colimycin) OR TS=(Colisticin) OR TS=(Coly-Mycin) OR TS=(Totazina) OR TS=(colistimethate sodium) OR TS=(Colistin Sulfate) OR TS=(CMS) OR TS=(colistin methanesulfonate) OR TS=(Sulfate, Colistin) OR TS=(Polymyxin B) OR TS=(Polymyxin) OR TS=(Polymyxins) OR TS=(Aerosporin) OR TS=(belcomycin) OR TS=(colicort) OR TS=(colimycine) OR TS=(colistine) OR TS=(colomycin) OR TS=(coly mycin) OR TS=(colymicin) OR TS=(colymycin) OR TS=(multimycin) OR TS=(polimixin) OR TS=(polimyxin) OR TS=(polymixin) OR TS=(polymycin) OR TS=(polymyxine) OR TS=(bacillosporin) OR TS=(poly-rx)) AND (TS=(Pneumonia) OR TS=(Pneumonias) OR TS=(Lobar Pneumonia) OR TS=(Lobar Pneumonias) OR TS=(Pneumonias, Lobar) OR TS=(Pneumonia, Lobar) OR TS=(Experimental Lung Inflammation) OR TS=(Experimental Lung Inflammations) OR TS=(Inflammation, Experimental Lung) OR TS=(Lung Inflammation, Experimental) OR TS=(Lung Inflammations, Experimental) OR TS=(Pneumonitis) OR TS=(Pneumonitides) OR TS=(Pulmonary Inflammation) OR TS=(Inflammation, Pulmonary) OR TS=(Inflammations, Pulmonary) OR TS=(Pulmonary Inflammations) OR TS=(Lung Inflammation) OR TS=(Inflammation, Lung) OR TS=(Inflammations, Lung) OR TS=(Lung Inflammations) OR TS=(pneumonic) OR TS=(inflammatory lung disease) OR TS=(lobitis) OR TS=(nonspecific inflammatory lung disease) OR TS=(peripneumonia) OR TS=(pleuropneumonia) OR TS=(pleuropneumonitis) OR TS=(pulmonal inflammation) OR TS=(pulmonic inflammation))  The search was conducted from the establishment of the library to November 15, 2023, and 910 results were retrieved. | 910 |
| Clinicaltrials | |
| Condition or disease：Lobar Pneumonia OR Lobar Pneumonias OR Pneumonia OR Experimental Lung Inflammation OR Pneumonitis OR Pneumonitides OR Pulmonary Inflammation OR Pulmonary Inflammations OR Lung Inflammations OR Lung Inflammation OR pleuropneumonia  Other terms：Colistin OR Polymyxin E OR Colimycin OR Colisticin OR Coly-Mycin OR Totazina OR colistimethate sodium OR Colistin Sulfate OR CMS OR colistin methanesulfonate OR Sulfate, Colistin OR Polymyxin B OR Polymyxin OR Polymyxins OR Aerosporin OR belcomycin OR colicort OR colimycine OR colistine OR colomycin OR coly mycin OR colymicin OR colymycin OR multimycin OR polimixin OR polimyxin OR polymixin OR polymycin OR polymyxine OR bacillosporin OR poly-rx  The search was conducted from the establishment of the library to November 15, 2023, and 58 results were retrieved. | 58 |
| World Health Organization International Clinical Trials Registry Platform | |
| (Pneumonia OR Pneumonias OR Lobar Pneumonia OR Lobar Pneumonias OR Pneumonias, Lobar OR Pneumonia, Lobar OR Experimental Lung Inflammation OR Experimental Lung Inflammations OR Inflammation, Experimental Lung OR Lung Inflammation, Experimental OR Lung Inflammations, Experimental OR Pneumonitis OR Pneumonitides OR Pulmonary Inflammation OR Inflammation, Pulmonary OR Inflammations, Pulmonary OR Pulmonary Inflammations OR Lung Inflammation OR Inflammation, Lung OR Inflammations, Lung OR Lung Inflammations OR pneumonic OR inflammatory lung disease OR lobitis OR nonspecific inflammatory lung disease OR peripneumonia OR pleuropneumonia OR pleuropneumonitis OR pulmonal inflammation OR pulmonic inflammation)[in the Condition] AND (Colistin OR Polymyxin E OR Colimycin OR Colisticin OR Coly-Mycin OR Totazina OR colistimethate sodium OR Colistin Sulfate OR colistin methanesulfonate OR Sulfate, Colistin OR Polymyxin B OR Polymyxin OR Polymyxins OR Aerosporin OR belcomycin OR colicort OR colimycine OR colistine OR colomycin OR coly mycin OR colymicin OR colymycin OR multimycin OR polimixin OR polimyxin OR polymixin OR polymycin OR polymyxine OR bacillosporin OR poly-rx OR CMS)[in the Intervention]  The search was conducted from the establishment of the library to November 15, 2023, and 363 results were retrieved. | 363 |
| medRxiv | |
| Pneumonia AND Polymyxin | 52 |
| Colistin AND Pneumonia | 106 |
| The search was conducted from the establishment of the library to November 15, 2023, and 158 results were retrieved. |  |
| Social Science Research Network | |
| Pneumonia Polymyxin | 4 |
| Colistin Pneumonia | 2 |
| CMS Pneumonia | 3 |
| The search was conducted from the establishment of the library to November 15, 2023, and 9 results were retrieved. |  |
| OCLC FirstSearch (Proceedings, PapersFirst) | |
| ((kw: injection) or (kw: Intravenous) or (kw: Parenteral) or (kw: I.V) or (kw: infusions) or (kw: infusion) or (kw: Injections) or (kw: IV) or (kw: venous w drip) or (kw: venous w transfusion) or (kw: Inhalation) or (kw: aerosol) or (kw: nebulization) or (kw: Aerosolized) or (kw: Nebulized) or (kw: inhaled) or (kw: Aerosolised) or (kw: Nebulised) or (kw: Inhaling) or (kw: Inspiration, w Respiratory) or (kw: Respiratory w Inspiration) or (kw: Spray) or (kw: parenteral) or (kw: aerosolexposure) or (kw: aerosolinhalation) or (kw: aerosolen) or (kw: aerosolization) or (kw: aerosols) or (kw: dentalaerosol) or (kw: pressurizedaerosol) or (kw: atomization) or (kw: mist w therapy) or (kw: ultrasonic w atomization)) and ((kw: Pneumonia) or (kw: Pneumonias) or (kw: Inflammation, w Experimental w Lung) or (kw: Pneumonitis) or (kw: Pneumonitides) or (kw: Pulmonary w Inflammation) or (kw: Inflammation, w Pulmonary) or (kw: Inflammations, w Pulmonary) or (kw: Pulmonary w Inflammations) or (kw: Lung w Inflammation) or (kw: Inflammation, w Lung) or (kw: Inflammations, w Lung) or (kw: Lung w Inflammations) or (kw: pneumonic) or (kw: inflammatory w lung w disease) or (kw: lobitis) or (kw: peripneumonia) or (kw: pleuropneumonia) or (kw: pleuropneumonitis) or (kw: pulmonal w inflammation) or (kw: pulmonic w inflammation)) and ((kw: Colistin) or (kw: Colimycin) or (kw: Colisticin) or (kw: Coly-Mycin) or (kw: Totazina) or (kw: colistimethate w sodium) or (kw: Colistin w Sulfate) or (kw: CMS) or (kw: colistin w methanesulfonate) or (kw: Sulfate, w Colistin) or (kw: Polymyxin) or (kw: Polymyxins) or (kw: Aerosporin) or (kw: belcomycin) or (kw: colicort) or (kw: colimycine) or (kw: colistine) or (kw: colomycin) or (kw: coly w mycin) or (kw: colymicin) or (kw: colymycin) or (kw: multimycin) or (kw: polimixin) or (kw: polimyxin) or (kw: polymixin) or (kw: polymycin) or (kw: polymyxine) or (kw: bacillosporin) or (kw: poly-rx))  The search was conducted from the establishment of the library to November 15, 2023, and 2 articles in PapersFirst and 61 conference literatures in Proceedings were retrieved. | 63 |

# Appendix 2: Risk of bias assessment

**Supplementary Table 1** Risk of bias assessment for a. RCTs (RoB-2 tool) and b. cohort studies (ROBINS-I tool*) studies.

a.

| Study | Randomization process | Deviations from the intended interventions | Missing outcome data | Measurement of outcome | Selection of the reported result | RoB-2 overall score |
| --- | --- | --- | --- | --- | --- | --- |
| Abdellatif,2016 | Low | Low | Low | Low | Low | Low |
| Hasan,2021 | Some concerns | Low | Low | Low | Some concerns | Some concerns |
| Moghaddam,2019 | Low | Some concerns | Low | Low | High risk | High risk |

b.

| Study | Domain 1: Confounding factors | Domain 2: Selection of participants | Domain 3: Intervention classification | Domain 4: Deviation from intervention | Domain 5: Missing data | Domain 6: Measurement of outcome | Domain 7: Selection of reported result | ROBINS-I overall score |
| --- | --- | --- | --- | --- | --- | --- | --- | --- |
| Ahn,2020 | Low | Low | Low | Low | Low | Low | Low | Low |
| Almangour,2021 | Low | Low | Low | Low | Moderate | Low | Low | Moderate |
| Amin,2013 | Moderate | Low | Low | Low | Low | Low | Low | Moderate |
| BOGOVIĆ,2014 | Low | Low | Low | Low | Low | Low | Low | Low |
| Cho,2016 | Serious | Serious | Low | Moderate | Low | Low | Low | Serious |
| Choe,2019 | Moderate | Low | Low | Moderate | Moderate | Low | Low | Moderate |
| Doshi,2013 | Low | Low | Low | Low | Serious | Low | Low | Serious |
| Jang,2017 | Serious | Low | Low | Low | Low | Low | Moderate | Serious |
| Kalin,2012 | Low | Low | Low | Low | Low | Low | Low | Low |
| Kim,2017 | Low | Low | Low | Low | Low | Low | Low | Low |
| Korbila,2010 | Low | Low | Low | Moderate | Low | Low | Low | Moderate |
| Zhou,2021 | Low | Low | Low | Low | Low | Low | Low | Low |
| Lin,2022 | Low | Low | Low | Low | Low | Low | Low | Low |
| Liu,2022 | Low | Low | Low | Moderate | Low | Low | Low | Moderate |
| Matijašević,2018 | Moderate | Low | Moderate | Low | Moderate | Low | Low | Moderate |
| Naesens,2011 | Serious | Low | Moderate | Moderate | Low | Low | Low | Serious |
| Zheng,2019 | Serious | Low | Moderate | Low | Serious | Low | Low | Serious |
| Wu,2023 | Low | Low | Low | Low | Low | Low | Low | Low |
| Shi,2023 | Moderate | Low | Low | Low | Low | Low | Low | Moderate |

RoB-2: risk of bias-2 tool, ROBINS-I: Risk of Bias In Non-randomized Studies of Interventions tool

*ROBINS-I is a tool which assesses the risk of bias in non-randomized studies of interventions by looking into pre-intervention, intervention and post-intervention domains. Studies with low risk of bias (judged to be at low risk of bias for all domains.) are comparable to well-performed randomized trials. Studies with moderate risk of bias (judged to be at low or moderate risk of bias for all domains) provides sound evidence for a nonrandomized study but cannot be considered comparable to a well-performed randomized trial. Studies with serious risk of bias (judged to be at serious risk of bias in at least one domain, but not at critical risk of bias in any domain) have some important problems in the design. Studies with critical risk of bias (judged to be at critical risk of bias in at least one domain) are too problematic to provide any useful evidence and should not be included in any synthesis.

**Appendix 3: Forest plots of pairwise meta-analysis**


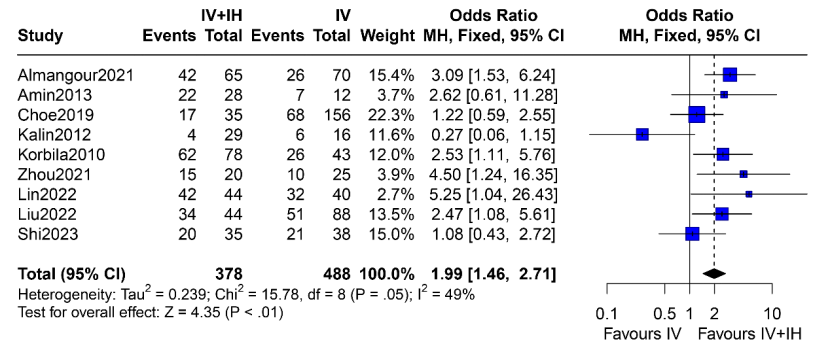


**Supplementary Fig. 1** clinical success (IV + IH vs. IV excluded high-risk studies)

IV + IH: intravenous plus inhaled polymyxins; IV: intravenous polymyxins; CI: confidence interval


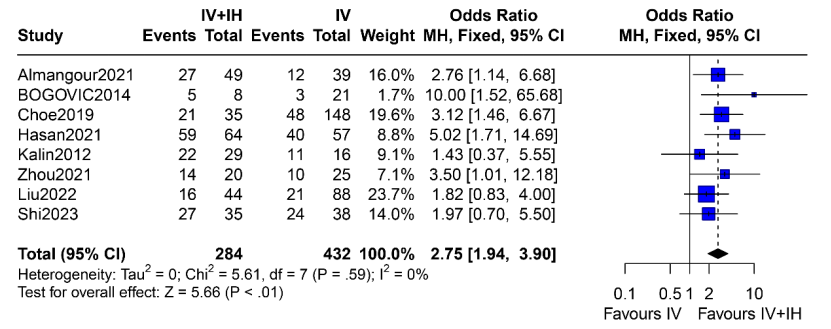


**Supplementary Fig. 2** microbial eradication rate (IV + IH vs. IV excluded high-risk studies)

IV + IH: intravenous plus inhaled polymyxins; IV: intravenous polymyxins; CI: confidence interval


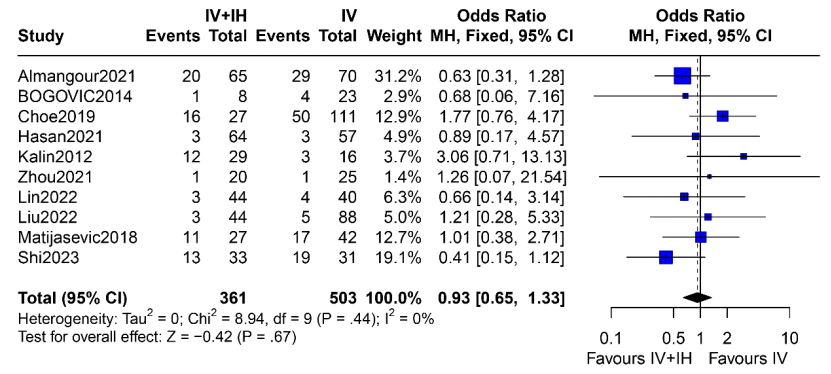


**Supplementary Fig. 3** acute kidney injury (IV + IH vs. IV excluded high-risk studies)

IV + IH: intravenous plus inhaled polymyxins; IV: intravenous polymyxins; CI: confidence interval


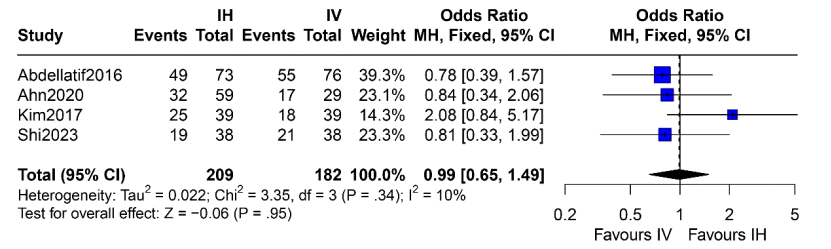


**Supplementary Fig. 4** clinical success (IH vs. IV excluded high-risk studies)

IH: inhaled polymyxins; IV: intravenous polymyxins; CI: confidence interval


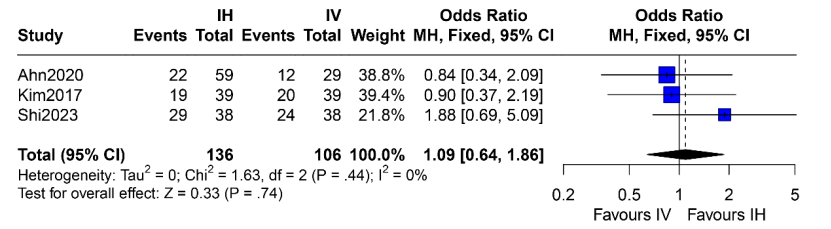


**Supplementary Fig. 5** microbial eradication rate (IH vs. IV excluded high-risk studies)

IH: inhaled polymyxins; IV: intravenous polymyxins; CI: confidence interval


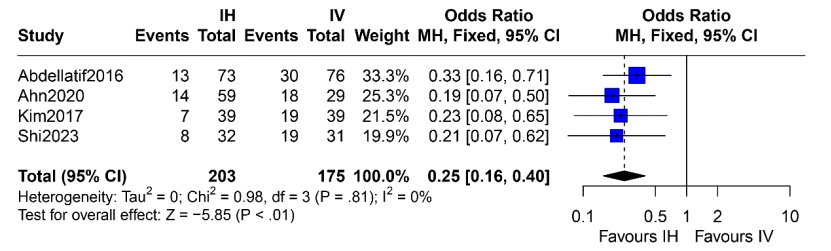


**Supplementary Fig. 6** acute kidney injury (IH vs. IV excluded high-risk studies)

IH: inhaled polymyxins; IV: intravenous polymyxins; CI: confidence interval


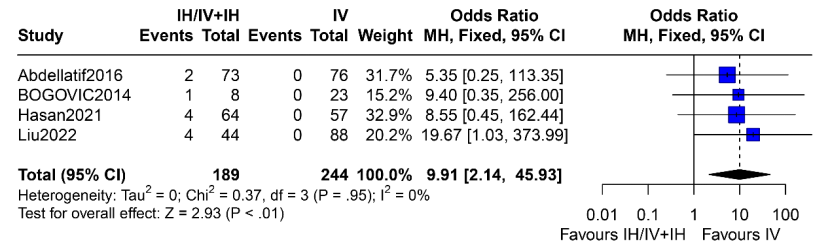


**Supplementary Fig. 7** incidence of bronchospasm (IH and IV + IH vs. IV excluded high-risk studies)

IH: inhaled polymyxins; IV + IH: intravenous plus inhaled polymyxins; IV: intravenous polymyxins; CI: confidence interval


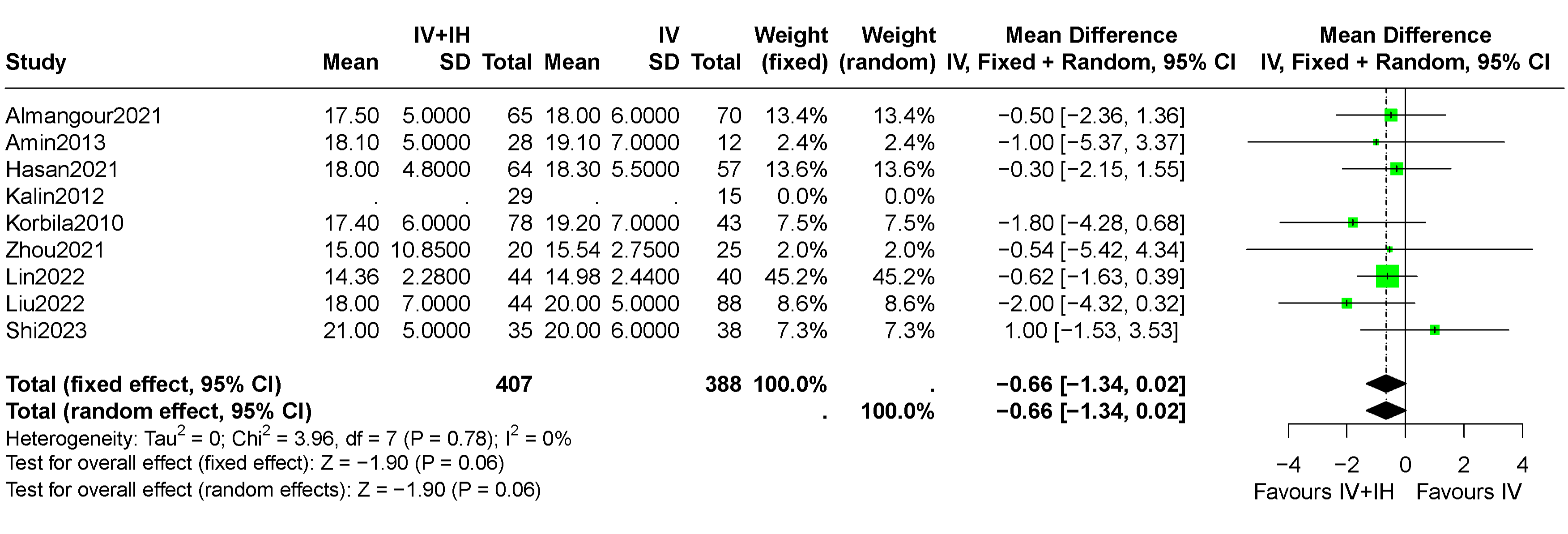


**Supplementary Fig. 8** baseline APACHE II score (IV + IH vs. IV excluded high-risk studies)

IV + IH: intravenous plus inhaled polymyxins; IV: intravenous polymyxins; CI: confidence interval


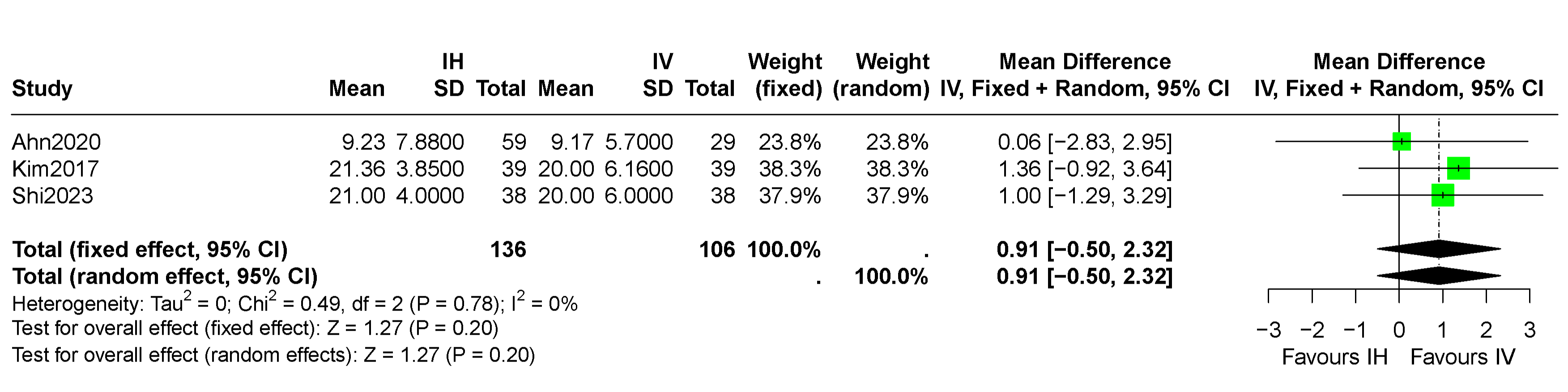


**Supplementary Fig. 9** baseline APACHE II score (IH vs. IV excluded high-risk studies)

IH: inhaled polymyxins; IV: intravenous polymyxins; CI: confidence interval

**Appendix 4: Sensitivity analyses and subgroup analyses**

Studies rated as having a high risk of bias in most terms of the Cochrane Bias Risk Tool were included in the sensitivity analysis. Regardless of whether these studies [1-6] were included, the meta-analysis results showed that the overall mortality of intravenous plus inhaled (IV + IH) polymyxin-containing regimen was significantly lower than intravenous (IV) polymyxin-containing regimen, and the clinical success and microbial eradication rate of IV + IH polymyxin-containing regimen were significantly higher than those of IV polymyxin-containing regimen, but there were no significant differences in acute kidney injury. In the comparison of inhaled (IH) and IV polymyxin-containing regimens, the rate of acute kidney injury was significantly lower in IH polymyxin-containing regimen, but the other three outcomes were not significantly different. Sensitivity analysis results are shown in Supplementary Figs. 10–17.


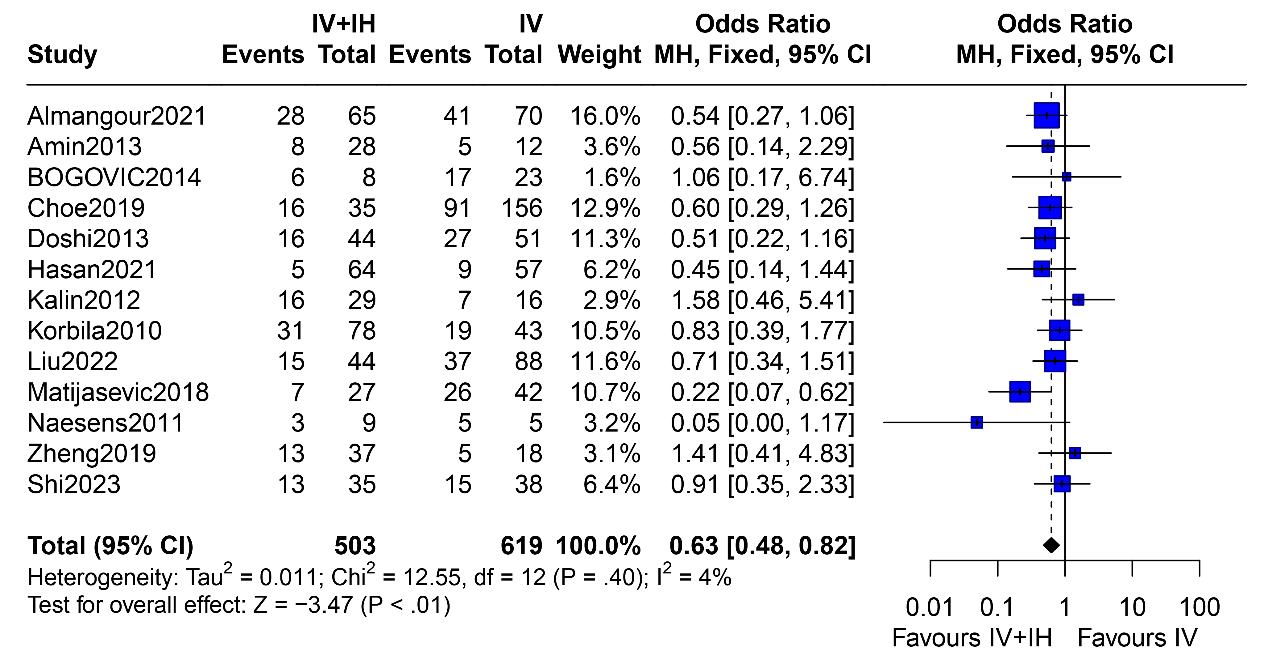


**Supplementary Fig. 10** overall mortality (IV + IH vs. IV included high-risk studies)

IV + IH: intravenous plus inhaled polymyxins; IV: intravenous polymyxins; CI: confidence interval


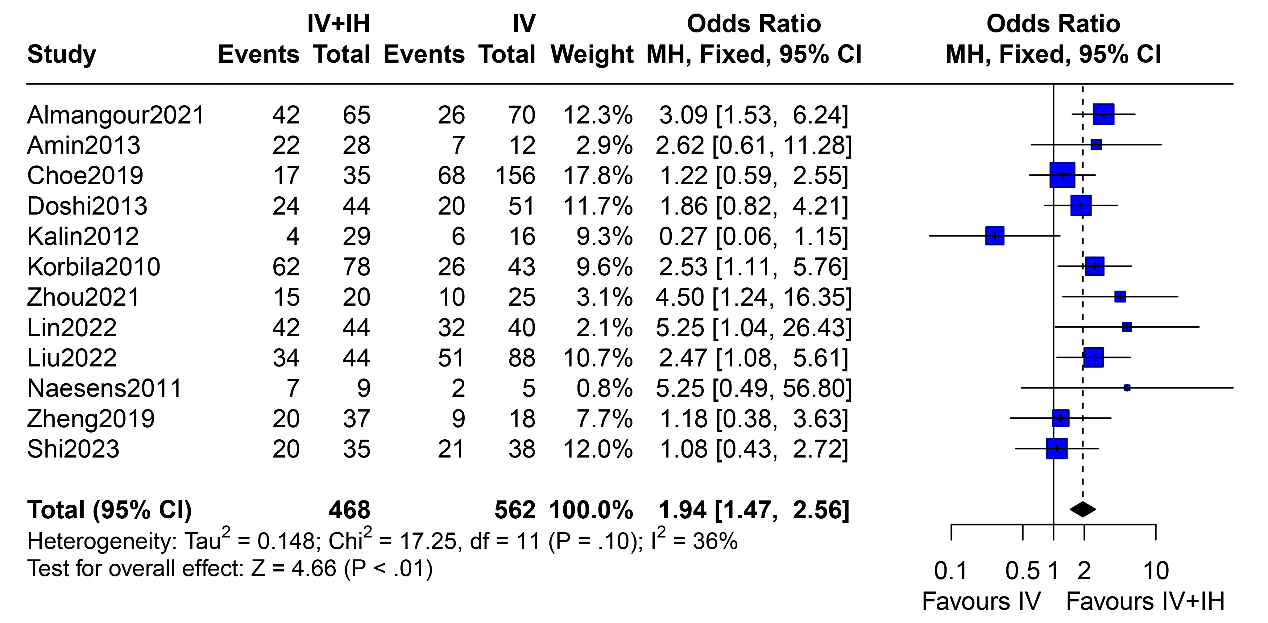


**Supplementary Fig. 11** clinical success (IV + IH vs. IV included high-risk studies)

IV + IH: intravenous plus inhaled polymyxins; IV: intravenous polymyxins; CI: confidence interval


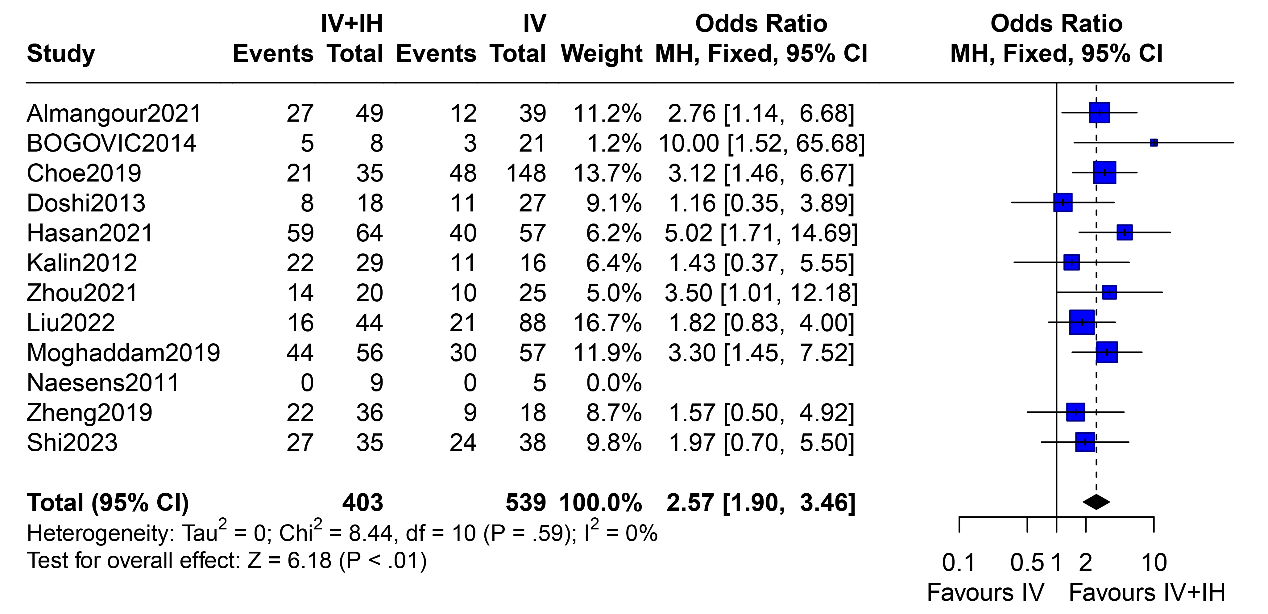


**Supplementary Fig. 12** microbial eradication rate (IV + IH vs. IV included high-risk studies)

IV + IH: intravenous plus inhaled polymyxins; IV: intravenous polymyxins; CI: confidence interval


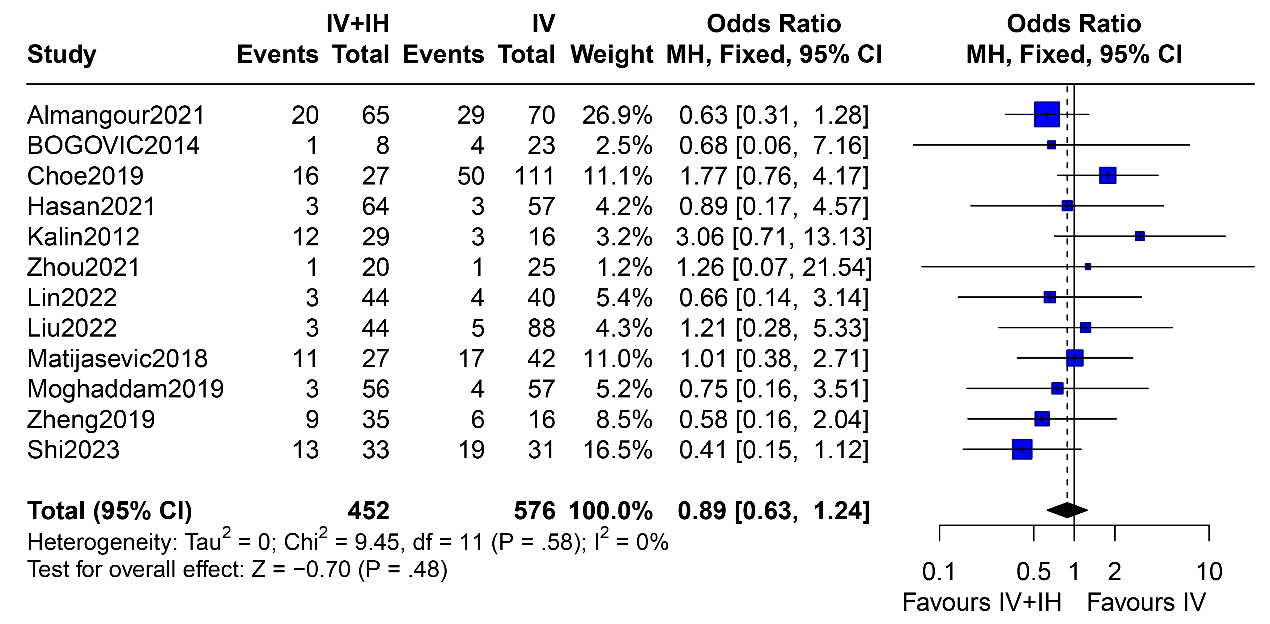


**Supplementary Fig. 13** acute kidney injury (IV + IH vs. IV included high-risk studies)

IV + IH: intravenous plus inhaled polymyxins; IV: intravenous polymyxins; CI: confidence interval


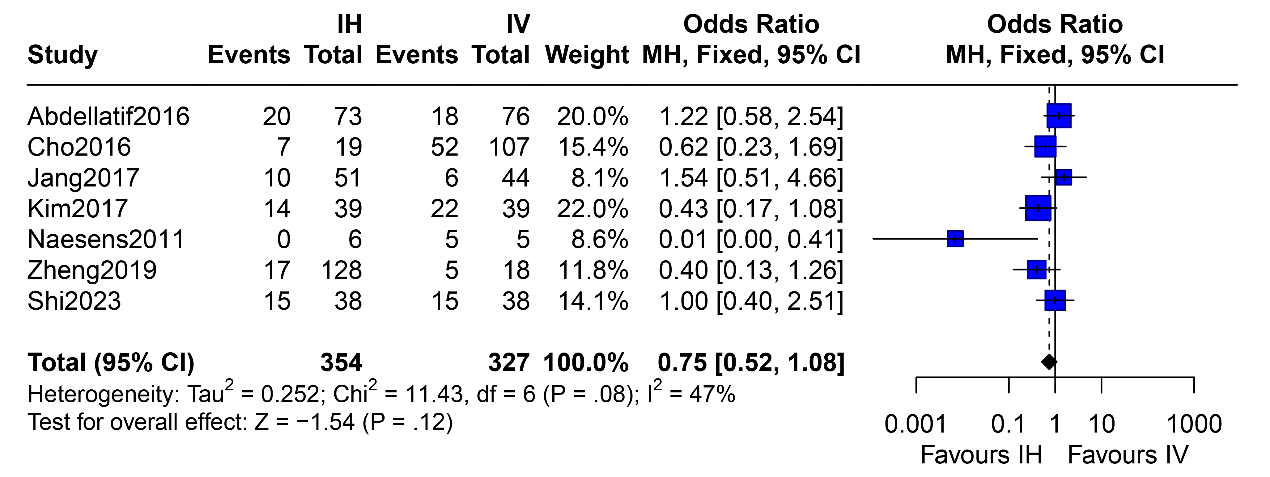


**Supplementary Fig. 14** overall mortality (IH vs. IV included high-risk studies)

IH: inhaled polymyxins; IV: intravenous polymyxins; CI: confidence interval


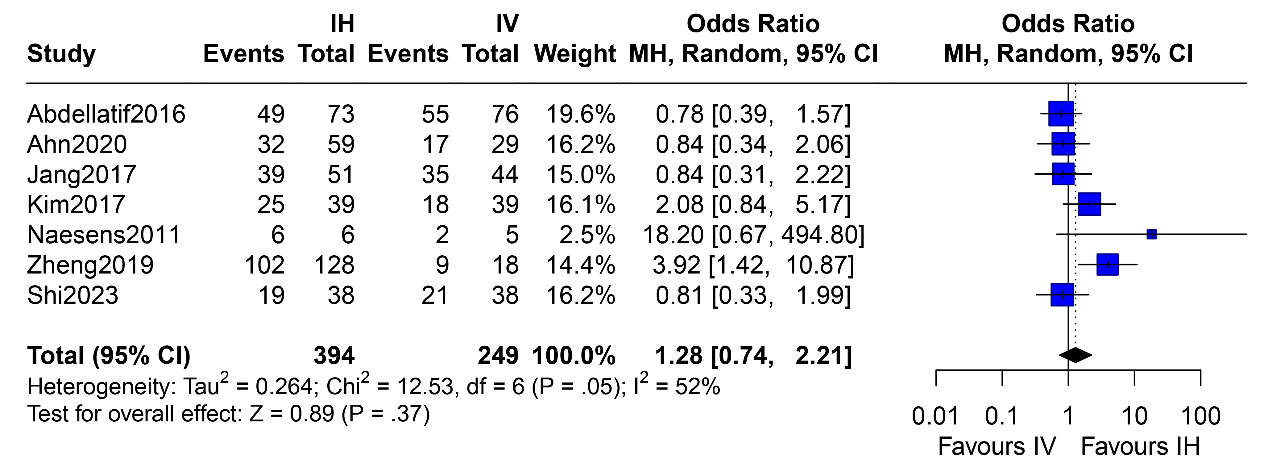


**Supplementary Fig. 15** clinical success (IH vs. IV included high-risk studies)

IH: inhaled polymyxins; IV: intravenous polymyxins; CI: confidence interval


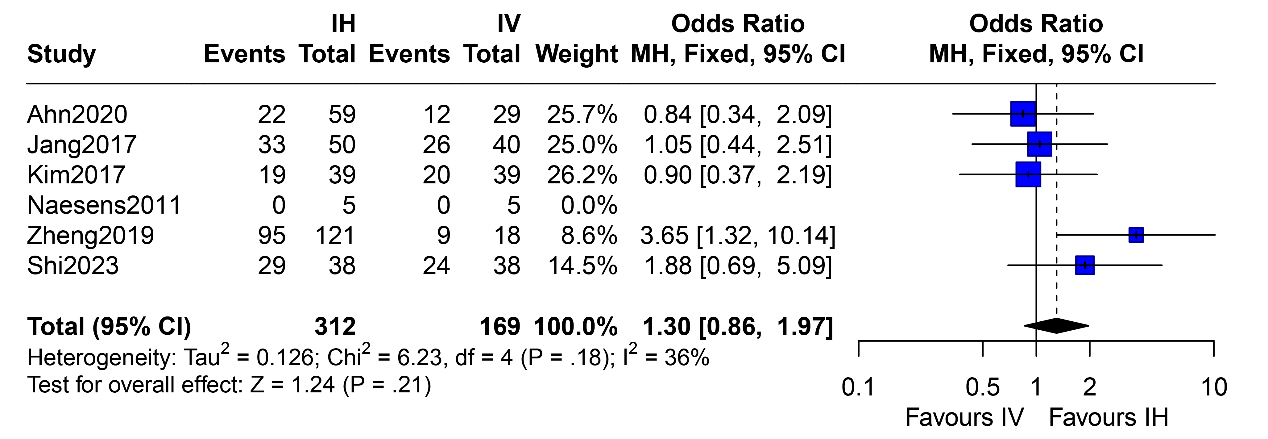


**Supplementary Fig. 16** microbial eradication rate (IH vs. IV included high-risk studies)

IH: inhaled polymyxins; IV: intravenous polymyxins; CI: confidence interval


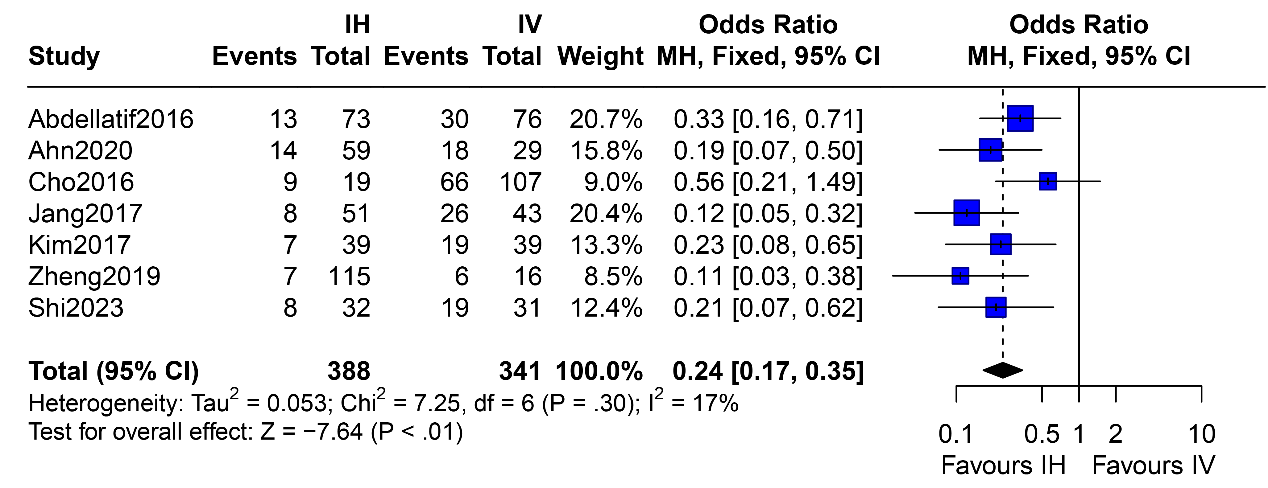


**Supplementary Fig. 17** acute kidney injury (IH vs. IV included high-risk studies)

IH: inhaled polymyxins; IV: intravenous polymyxins; CI: confidence interval

To investigate the impact of different polymyxins administration regimens on short-term overall mortality (mortality at maximum follow-up of 30 days), we conducted sensitivity analyses that encompassed studies reporting pertinent outcomes. Results are shown in Supplementary Figs. 18–19.


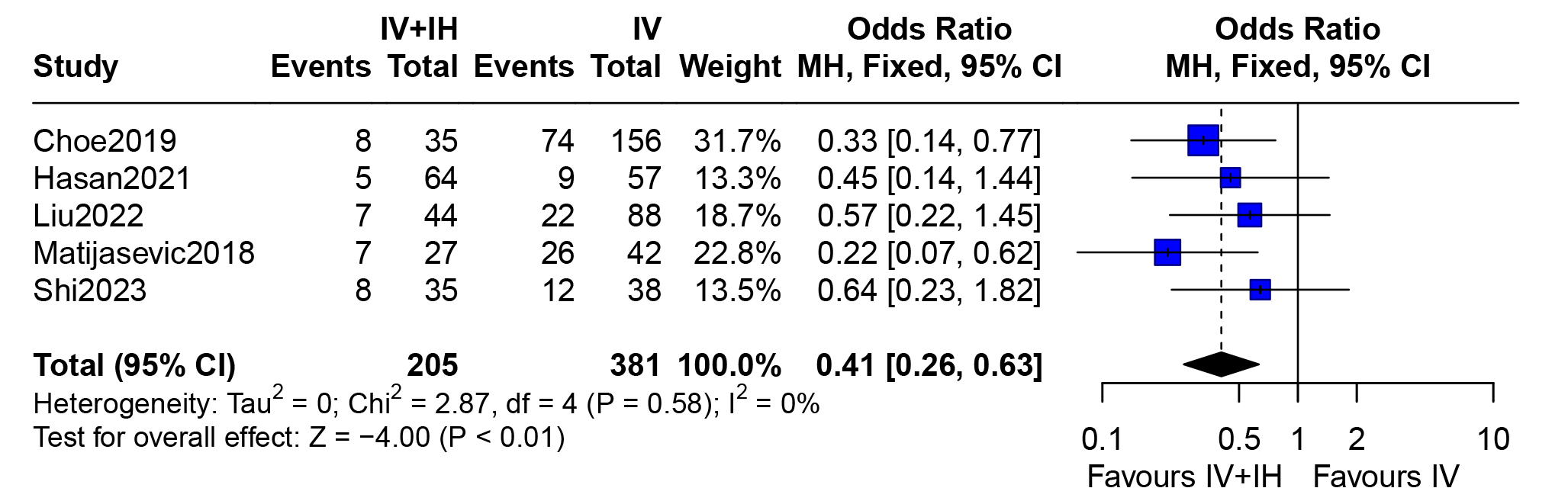


**Supplementary Fig. 18** short-term overall mortality (IV + IH vs. IV excluded high-risk studies)

IV + IH: intravenous plus inhaled polymyxins; IV: intravenous polymyxins; CI: confidence interval


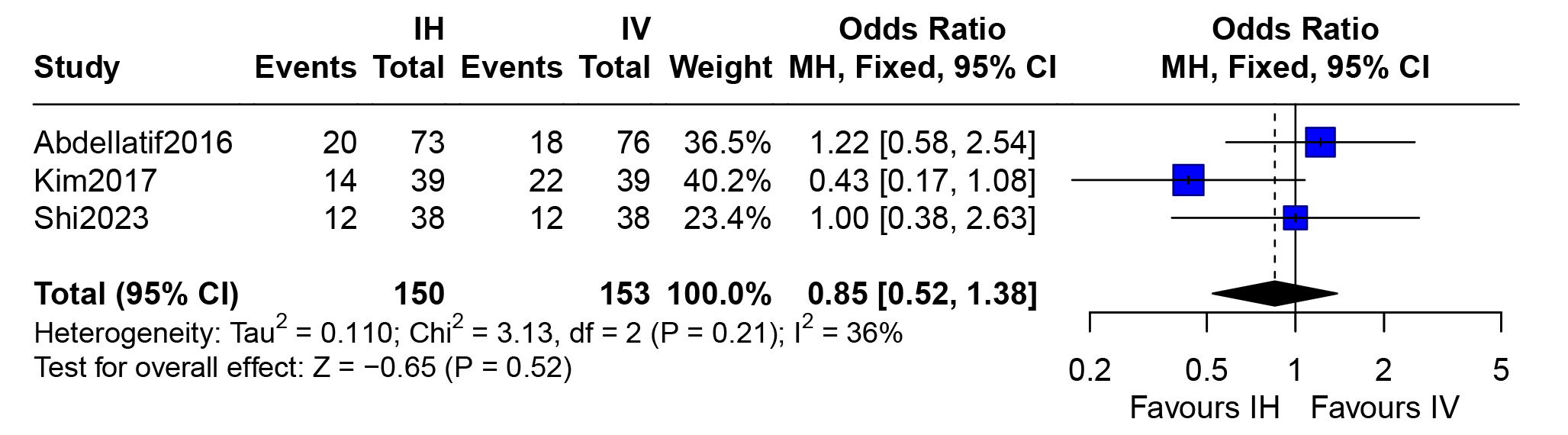


**Supplementary Fig. 19** short-term overall mortality (IH vs. IV excluded high-risk studies)

IH: inhaled polymyxins; IV: intravenous polymyxins; CI: confidence interval

In order to investigate the uncertainty of the effect estimates due to losses to follow-up, we conducted sensitivity analyses using imputations of missing outcome data in best-worst and worst-best case scenarios, as shown in Supplementary Figs. 20–21


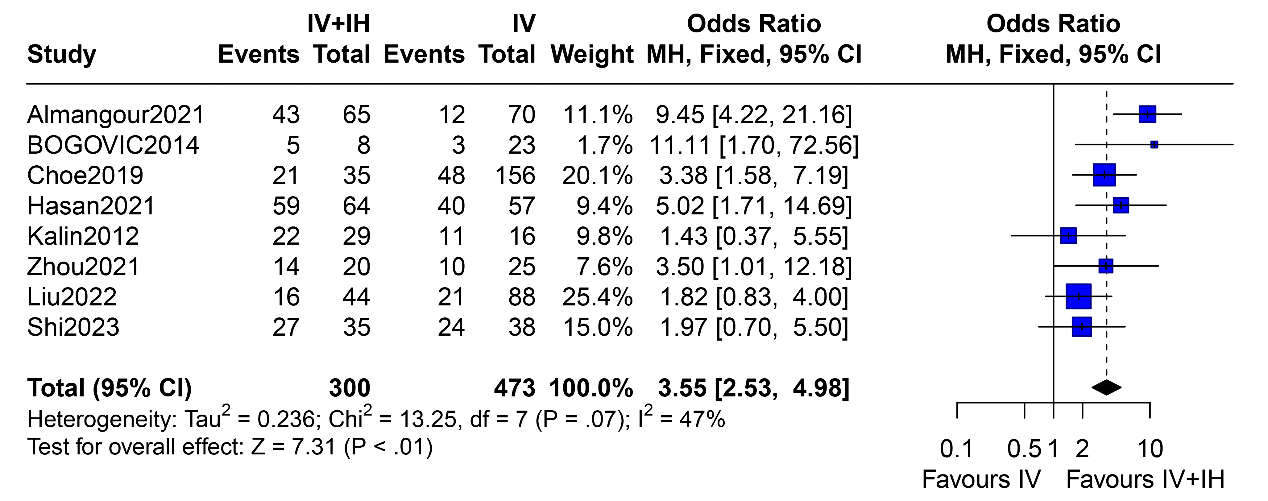


**Supplementary Fig. 20** best case analysis of microbial eradication rate (IV + IH vs. IV excluded high-risk studies)

IV + IH: intravenous plus inhaled polymyxins; IV: intravenous polymyxins; CI: confidence interval


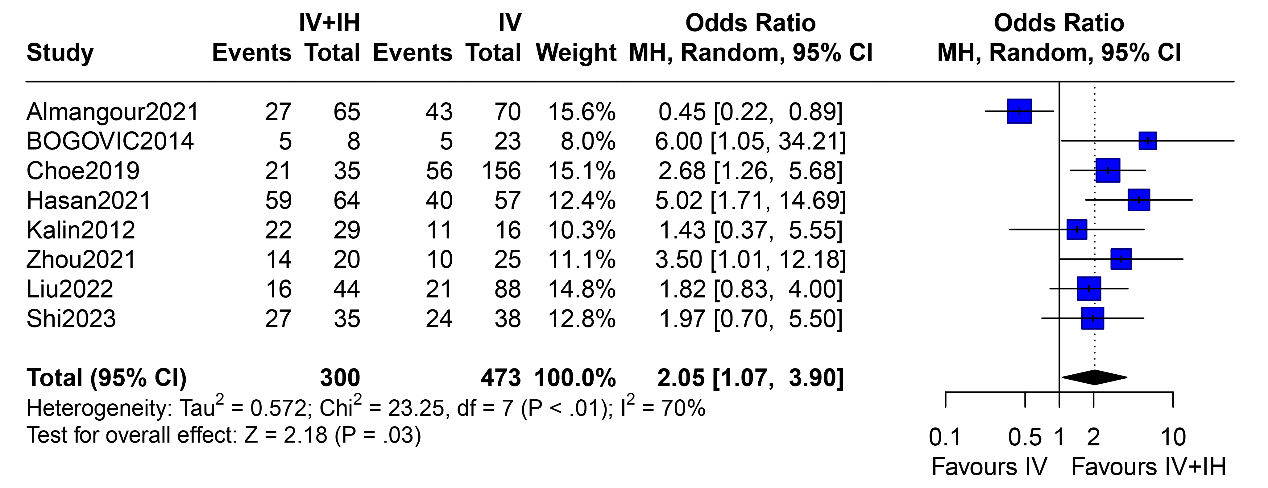


**Supplementary Fig. 21** worst case analysis of microbial eradication rate (IV + IH vs. IV excluded high-risk studies)

IV + IH: intravenous plus inhaled polymyxins; IV: intravenous polymyxins; CI: confidence interval

Given the substantial missing data observed in Matijašević et al.'s study, a sensitivity analysis was conducted with its exclusion.


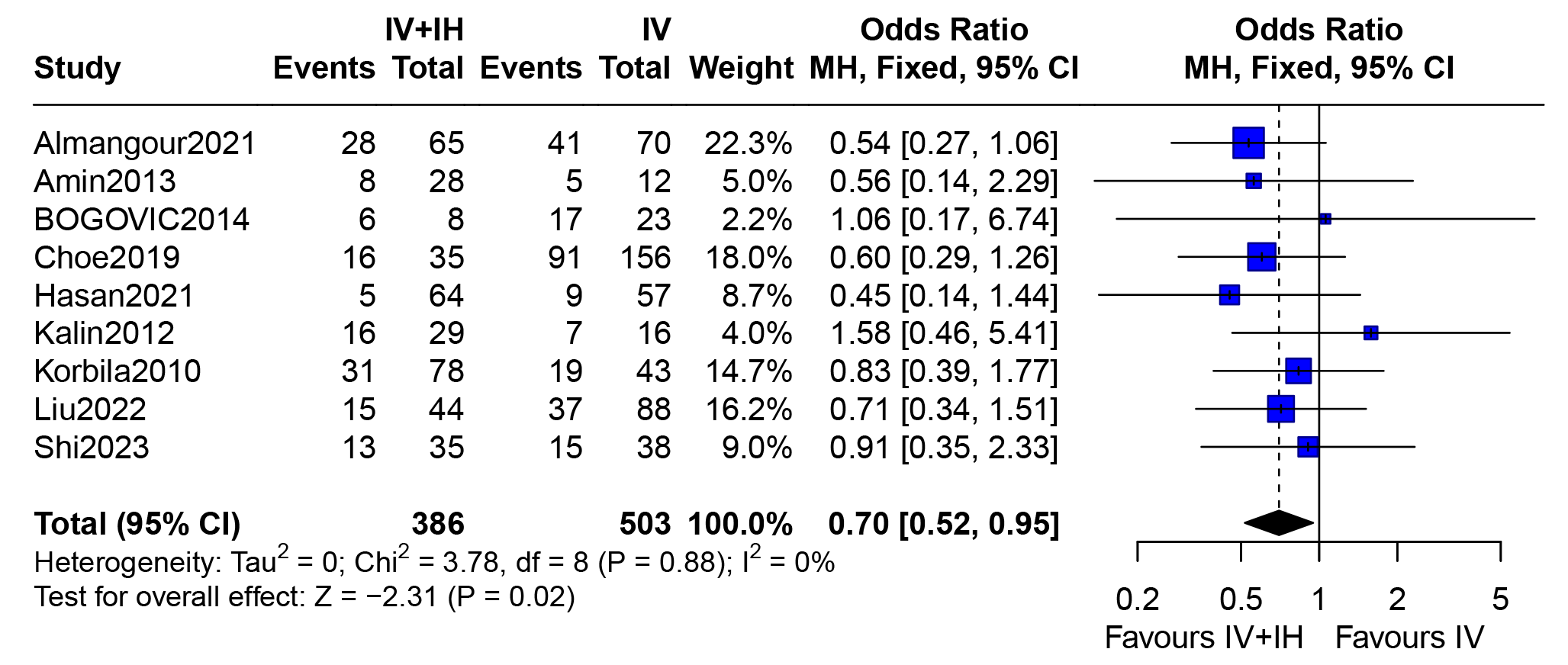


**Supplementary Fig. 22** overall mortality (IV + IH vs. IV, excluded high-risk studies and Matijašević et al.'s study )

IV + IH: intravenous plus inhaled polymyxins; IV: intravenous polymyxins; CI: confidence interval

Subgroup analysis was conducted according to the study type, the results are shown in Supplementary Fig. 23–29. The meta-analysis results of overall mortality and incidence of bronchospasm comparing intravenous plus inhaled (IV + IH) polymyxins and intravenous (IV) polymyxin-containing regimens were different in cohort studies and RCT. For overall mortality comparing IV + IH and IV polymyxin-containing regimens, the meta-analyses results of cohort studies showed that overall mortality was significantly decreased in IV + IH polymyxin-containing regimen (OR = 0.65; 95% CI, 0.48–0.88; P < 0.01; I² = 0%; Supplementary Fig. 23), while the RCT result did not show significant differences (OR = 0.45; 95% CI, 0.14–1.44; P = 0.18). For the incidence of bronchospasm, the meta-analysis results of cohort studies showed that IV + IH and inhaled (IH) polymyxin-containing regimens significantly increased the incidence of bronchospasm compared to IV polymyxin-containing regimen (OR = 10.58; 95% CI, 1.78–62.83; P < 0.01; I² = 0%; Supplementary Fig. 29, while no significant differences were observed in the RCT (OR = 8.55; 95% CI, 0.45–162.44; P = 0.15). Except for overall mortality and incidence of bronchospasm comparison, the meta-analysis results of other outcomes were not significantly different between cohort studies and RCTs.


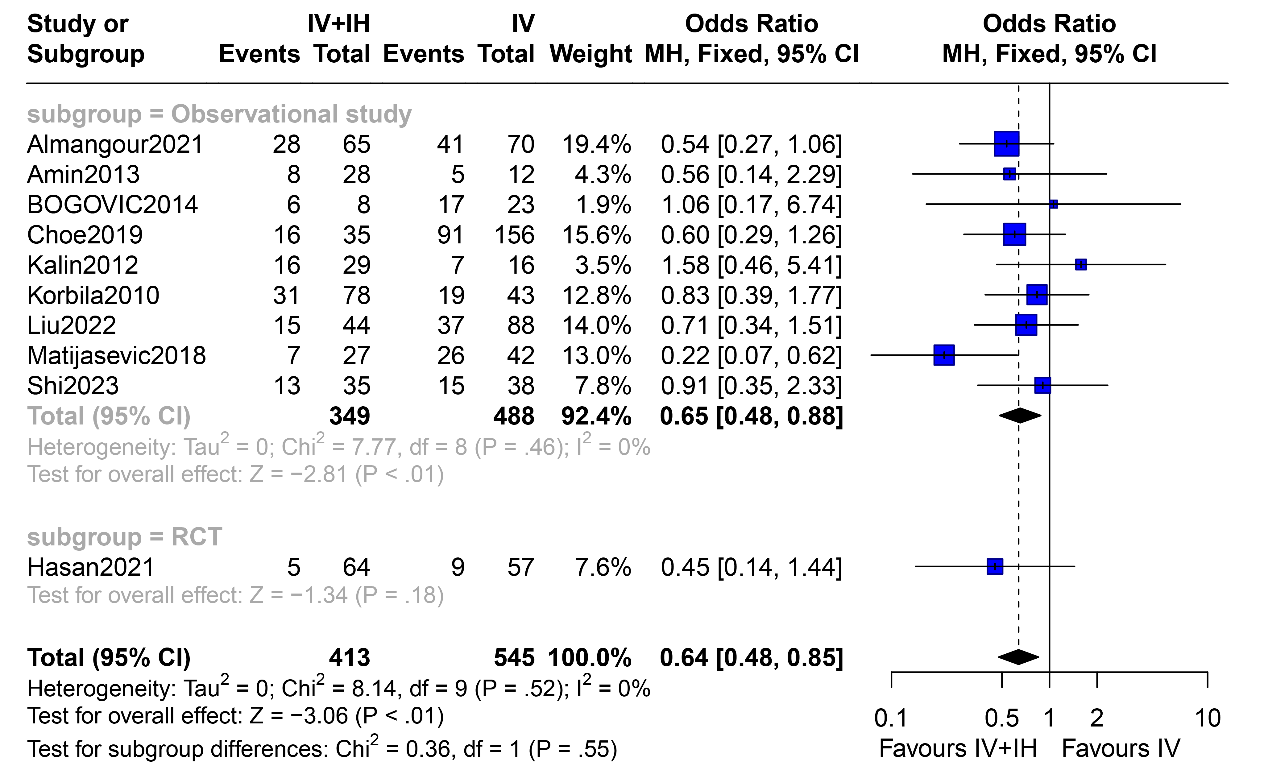


**Supplementary Fig. 23** subgroup analysis of overall mortality (IV + IH vs. IV excluded high-risk studies)

IV + IH: intravenous plus inhaled polymyxins; IV: intravenous polymyxins; CI: confidence interval


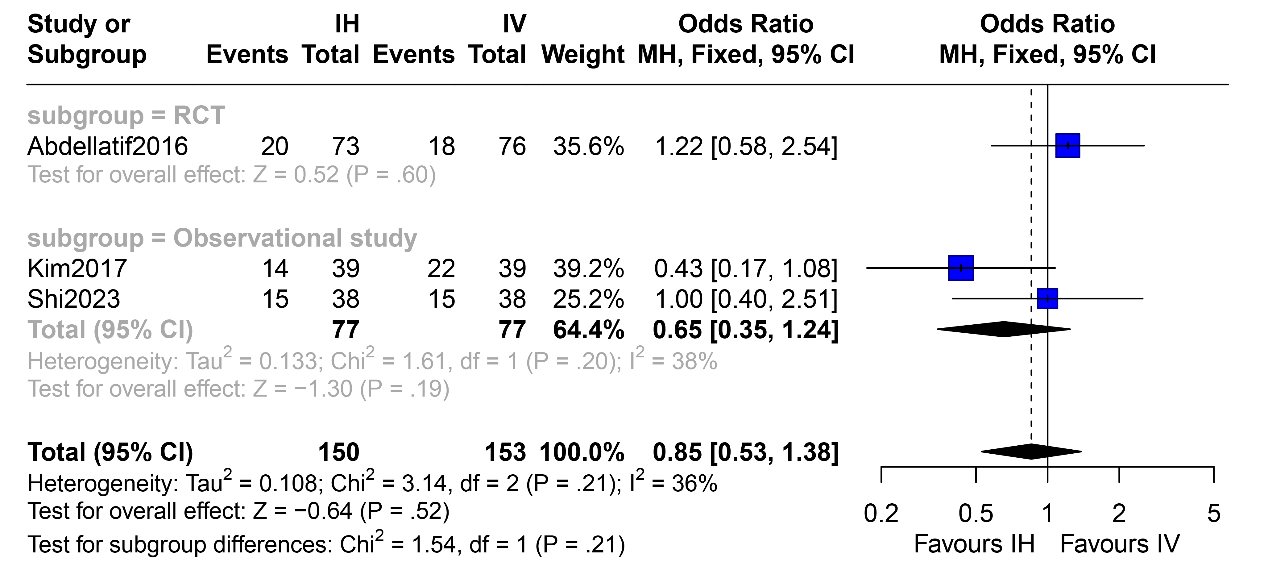


**Supplementary Fig. 24** subgroup analysis of overall mortality (IH vs. IV excluded high-risk studies)

IH: inhaled polymyxins; IV: intravenous polymyxins; CI: confidence interval


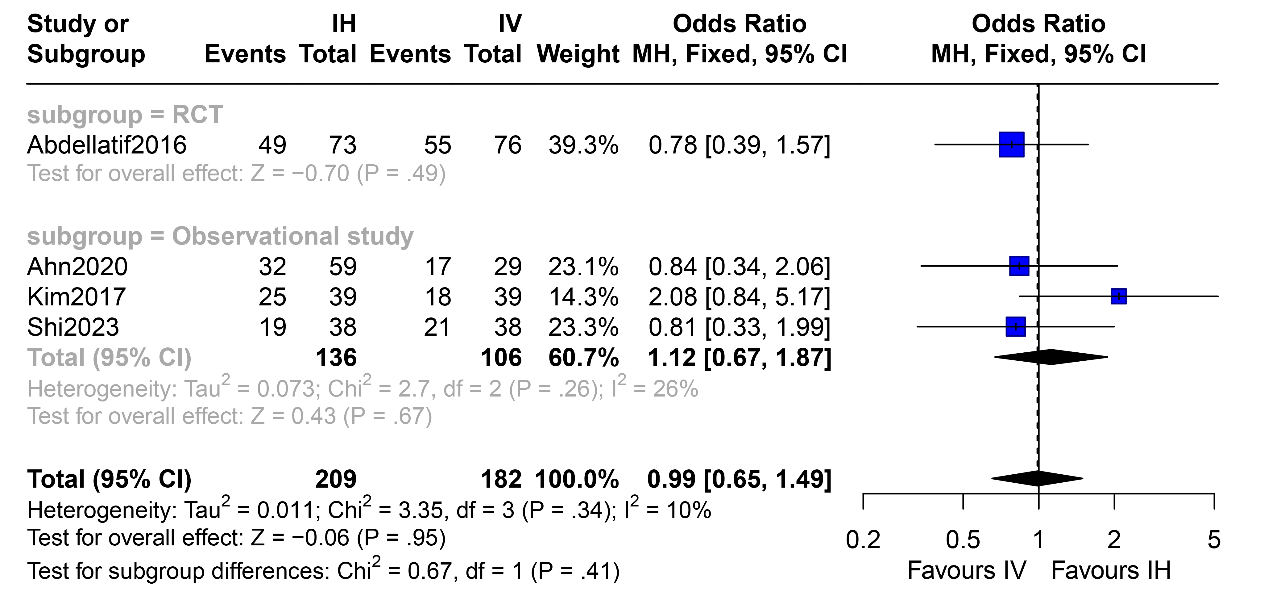


**Supplementary Fig. 25** subgroup analysis of clinical success (IH vs. IV excluded high-risk studies)

IH: inhaled polymyxins; IV: intravenous polymyxins; CI: confidence interval


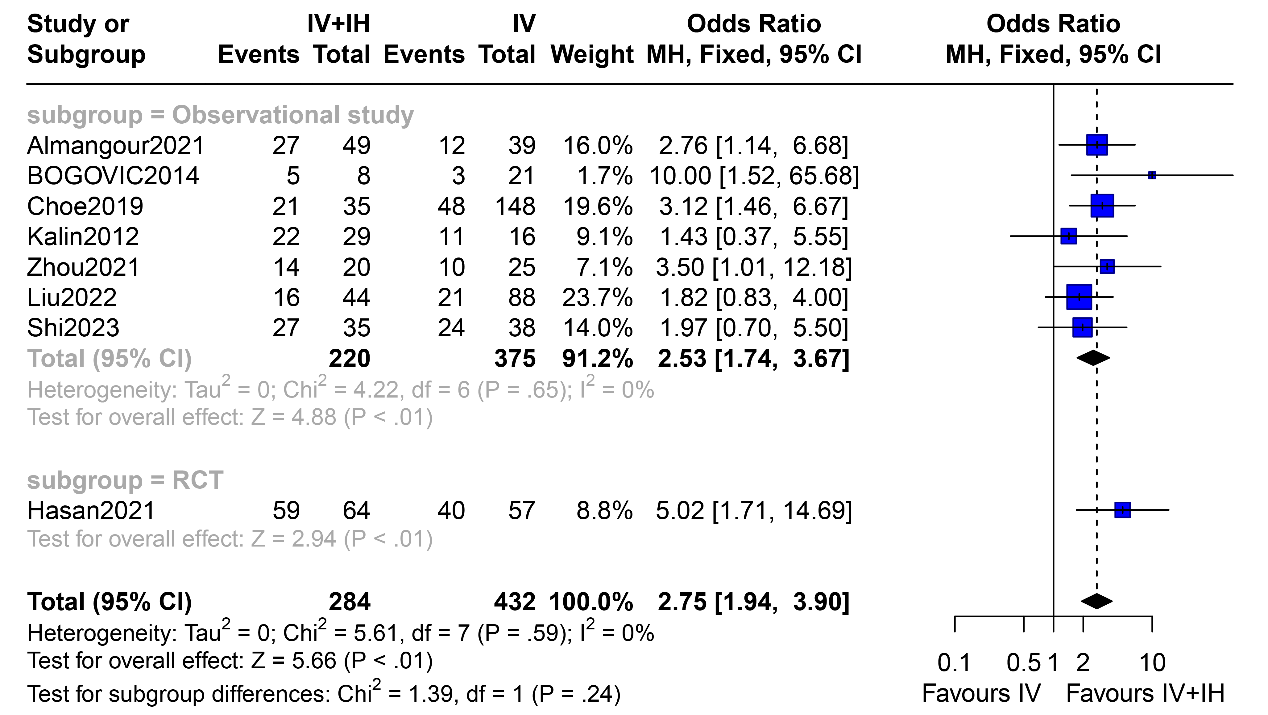


**Supplementary Fig. 26** subgroup analysis of microbial eradication rate (IV + IH vs. IV excluded high-risk studies)

IV + IH: intravenous plus inhaled polymyxins; IV: intravenous polymyxins; CI: confidence interval


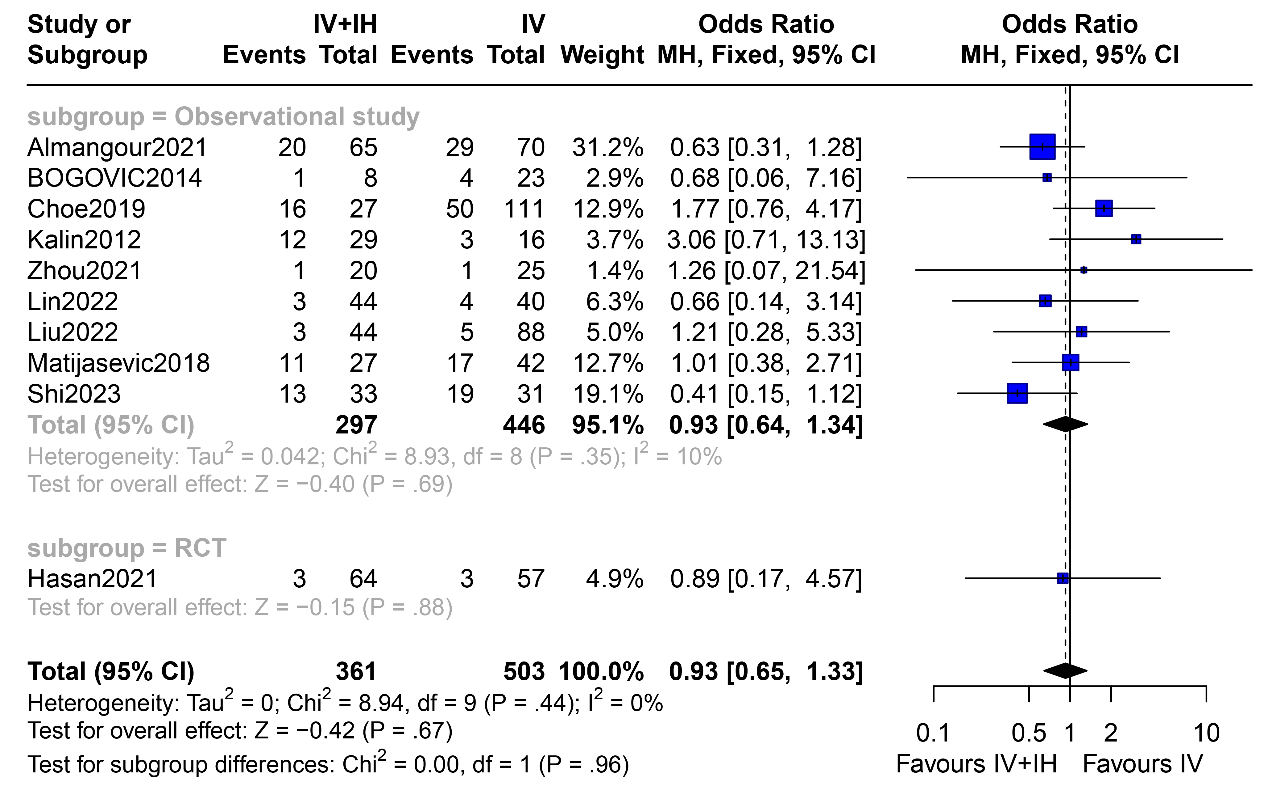


**Supplementary Fig. 27** subgroup analysis of acute kidney injury (IV + IH vs. IV excluded high-risk studies)

IV + IH: intravenous plus inhaled polymyxins; IV: intravenous polymyxins; CI: confidence interval


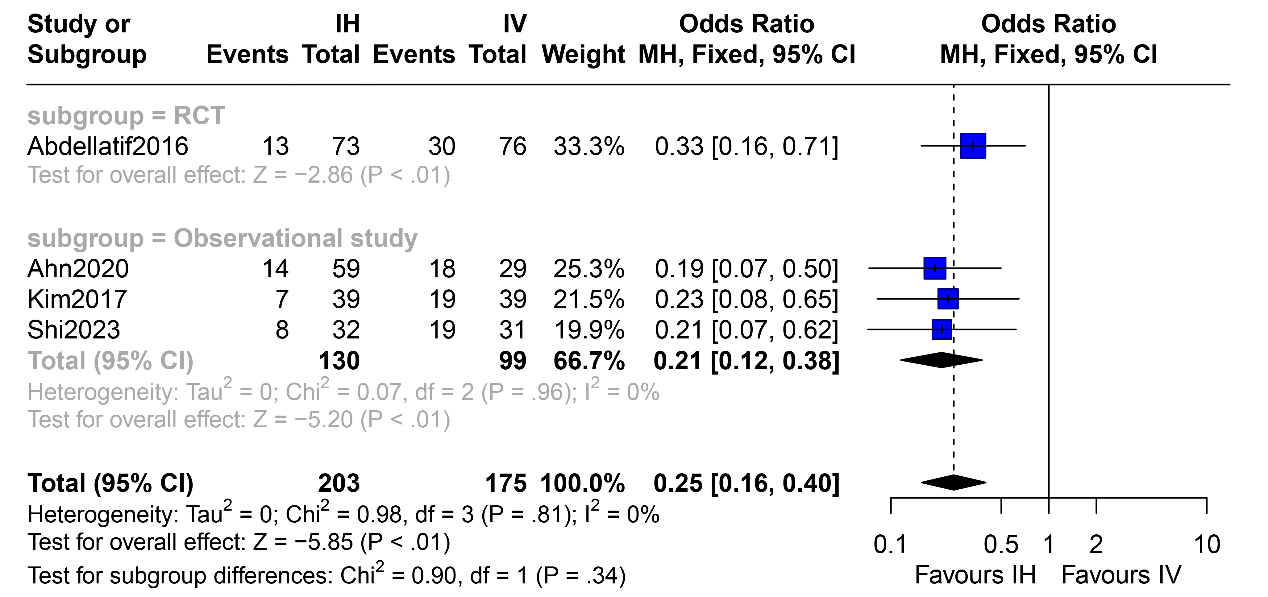


**Supplementary Fig. 28** subgroup analysis of acute kidney injury (IH vs. IV excluded high-risk studies)

IH: inhaled polymyxins; IV: intravenous polymyxins; CI: confidence interval


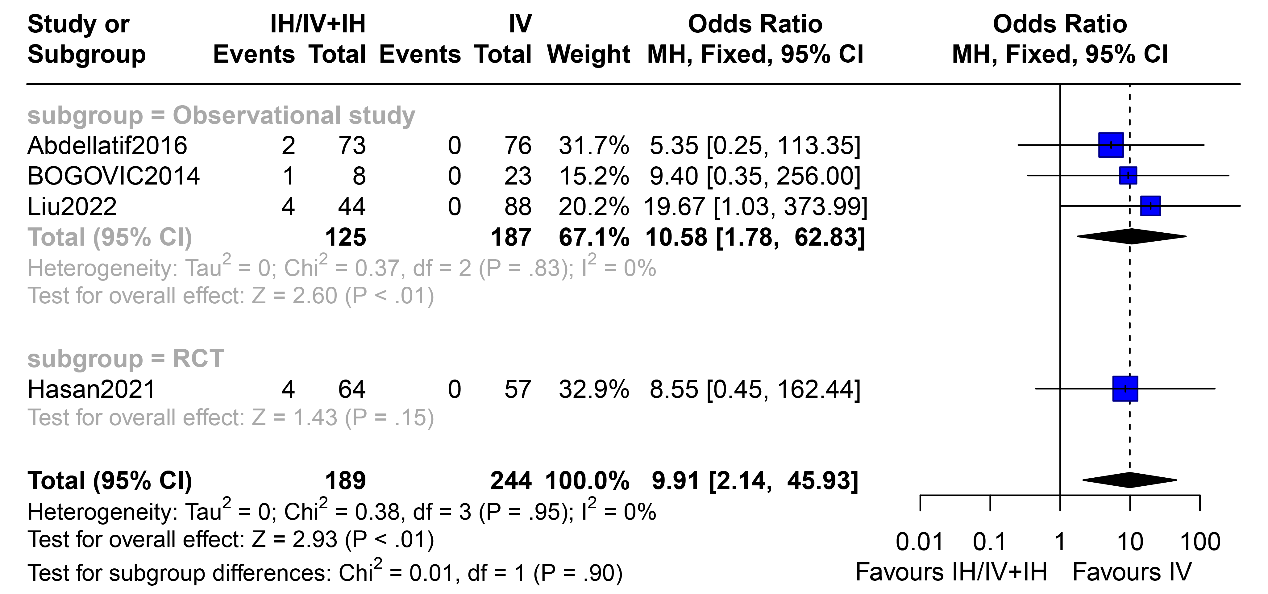


**Supplementary Fig. 29** subgroup analysis of incidence of bronchospasm (IH and IV + IH vs. IV excluded high-risk studies)

IH: inhaled polymyxins; IV + IH: intravenous plus inhaled polymyxins; IV: intravenous polymyxins; CI: confidence interval

Subgroup analyses results for different polymyxins are shown in Supplementary Fig. 30–37.


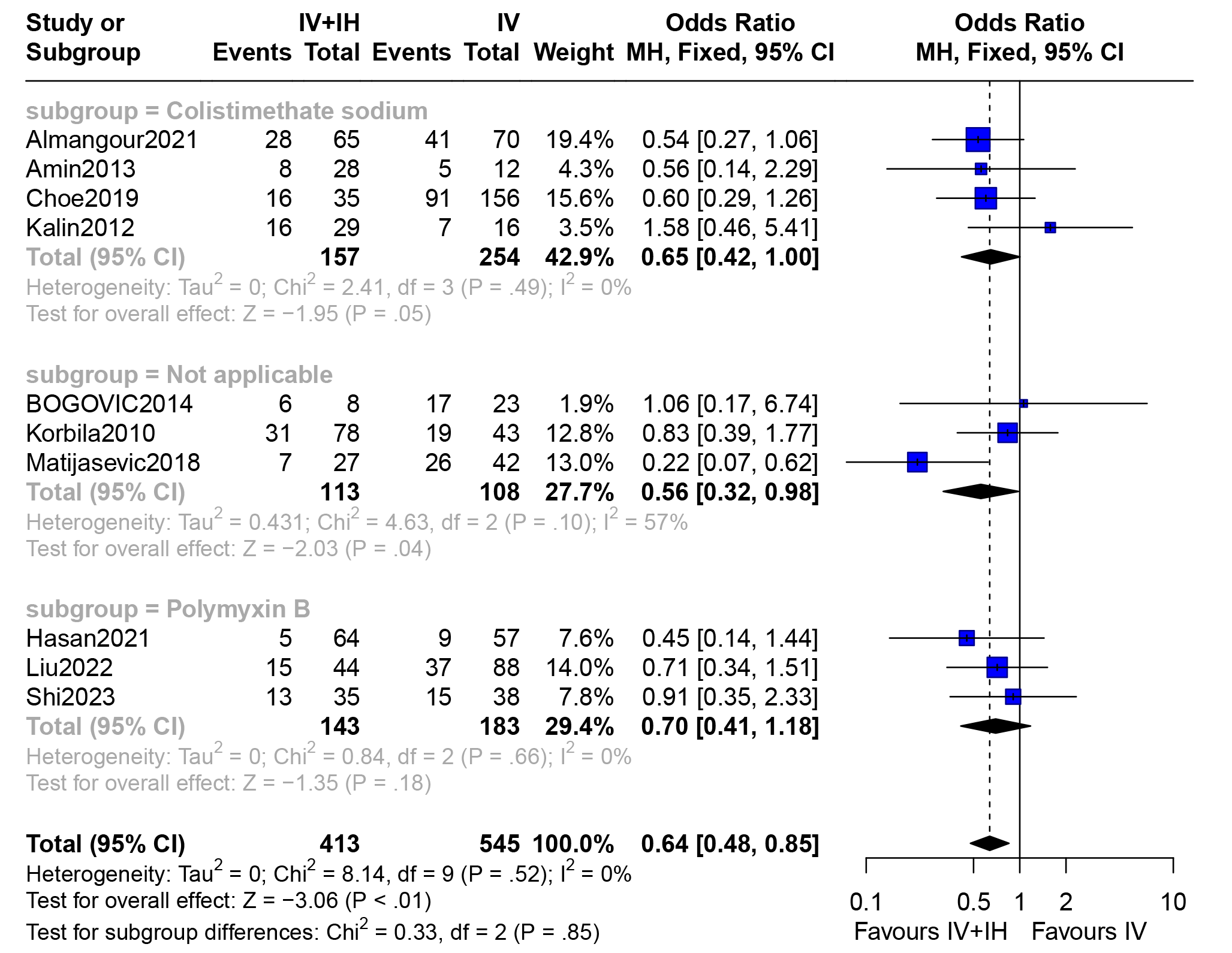


**Supplementary Fig. 30** subgroup analysis of overall mortality (IV + IH vs. IV excluded high-risk studies)

IV + IH: intravenous plus inhaled polymyxins; IV: intravenous polymyxins; CI: confidence interval


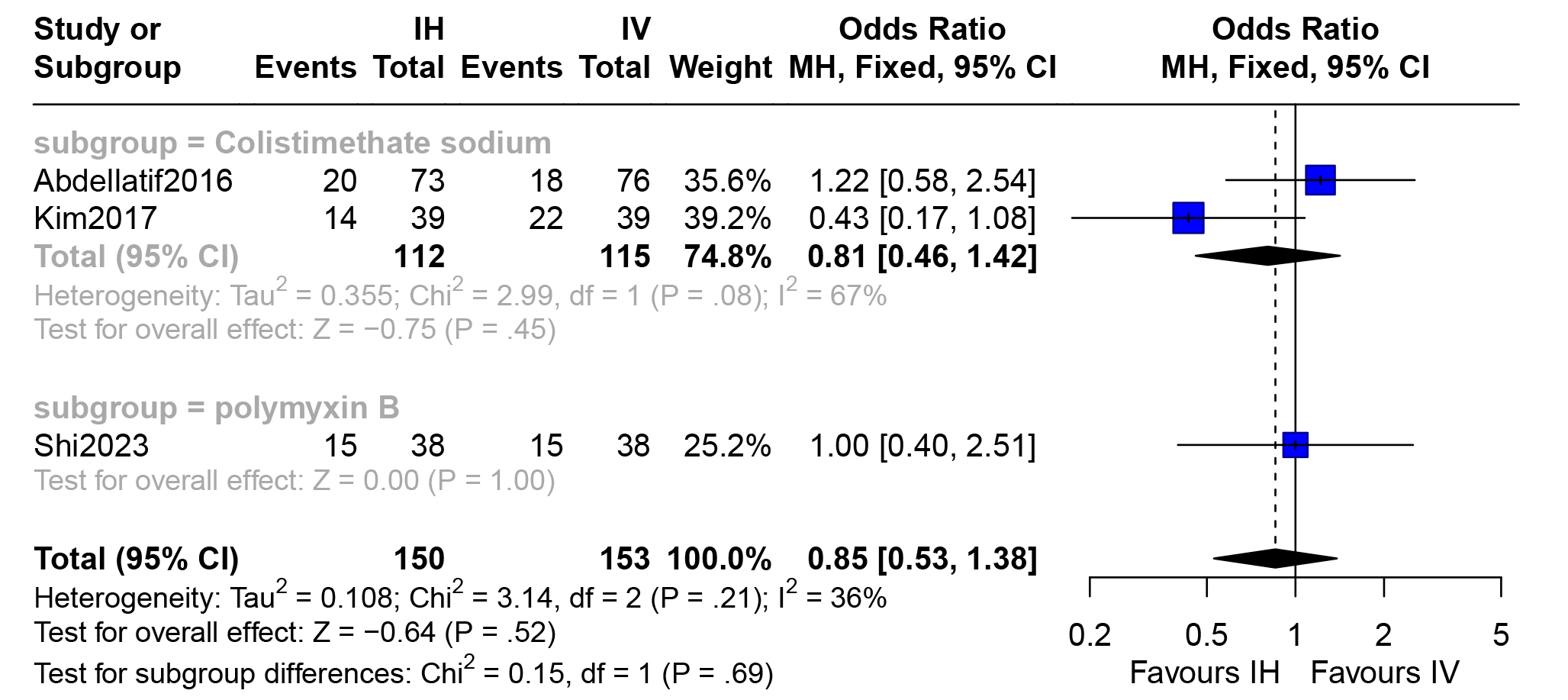


**Supplementary Fig. 31** subgroup analysis of overall mortality (IH vs. IV excluded high-risk studies)

IH: inhaled polymyxins; IV: intravenous polymyxins; CI: confidence interval


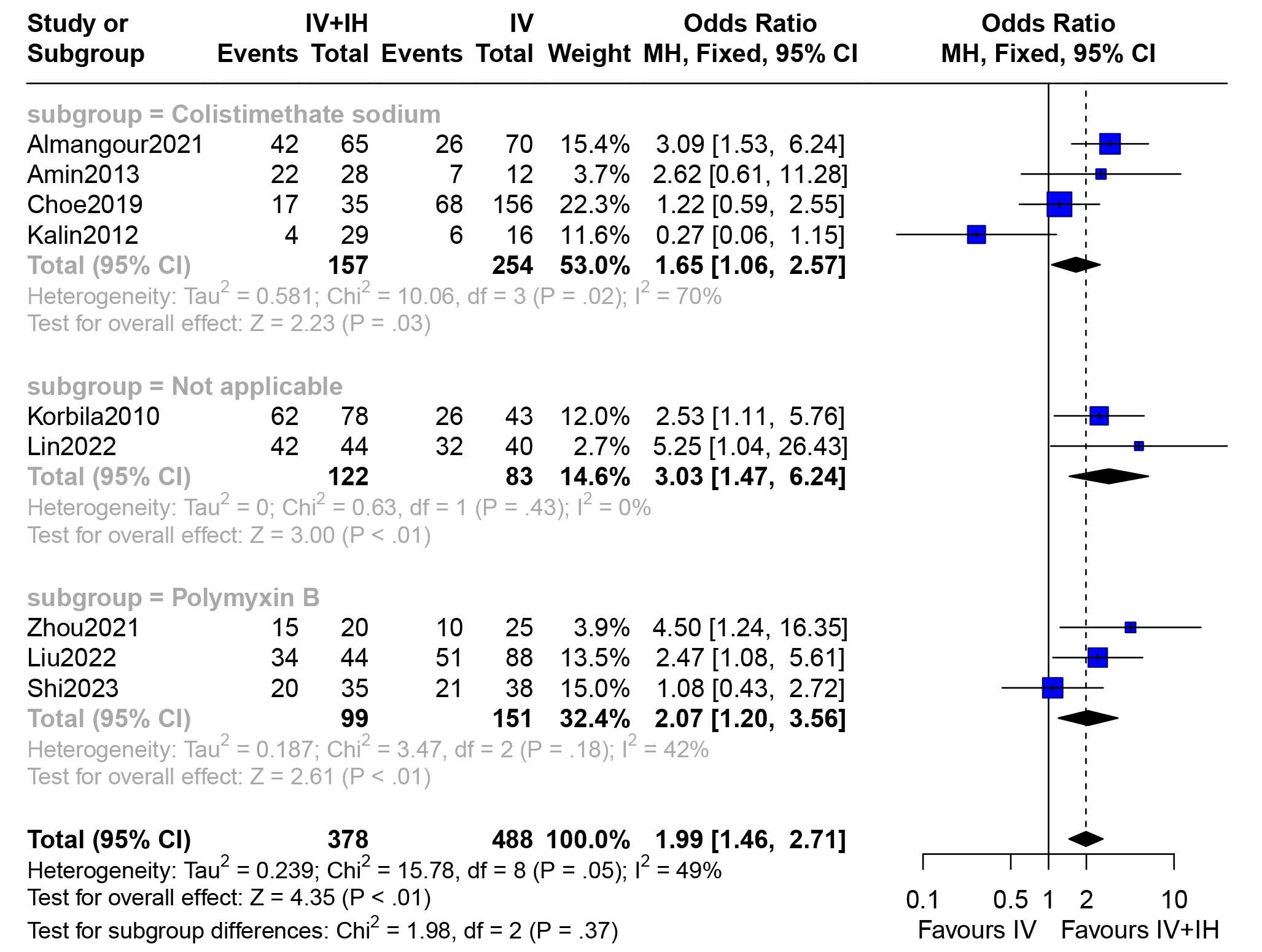


**Supplementary Fig. 32** subgroup analysis of clinical success (IV + IH vs. IV excluded high-risk studies)

IV + IH: intravenous plus inhaled polymyxins; IV: intravenous polymyxins; CI: confidence interval


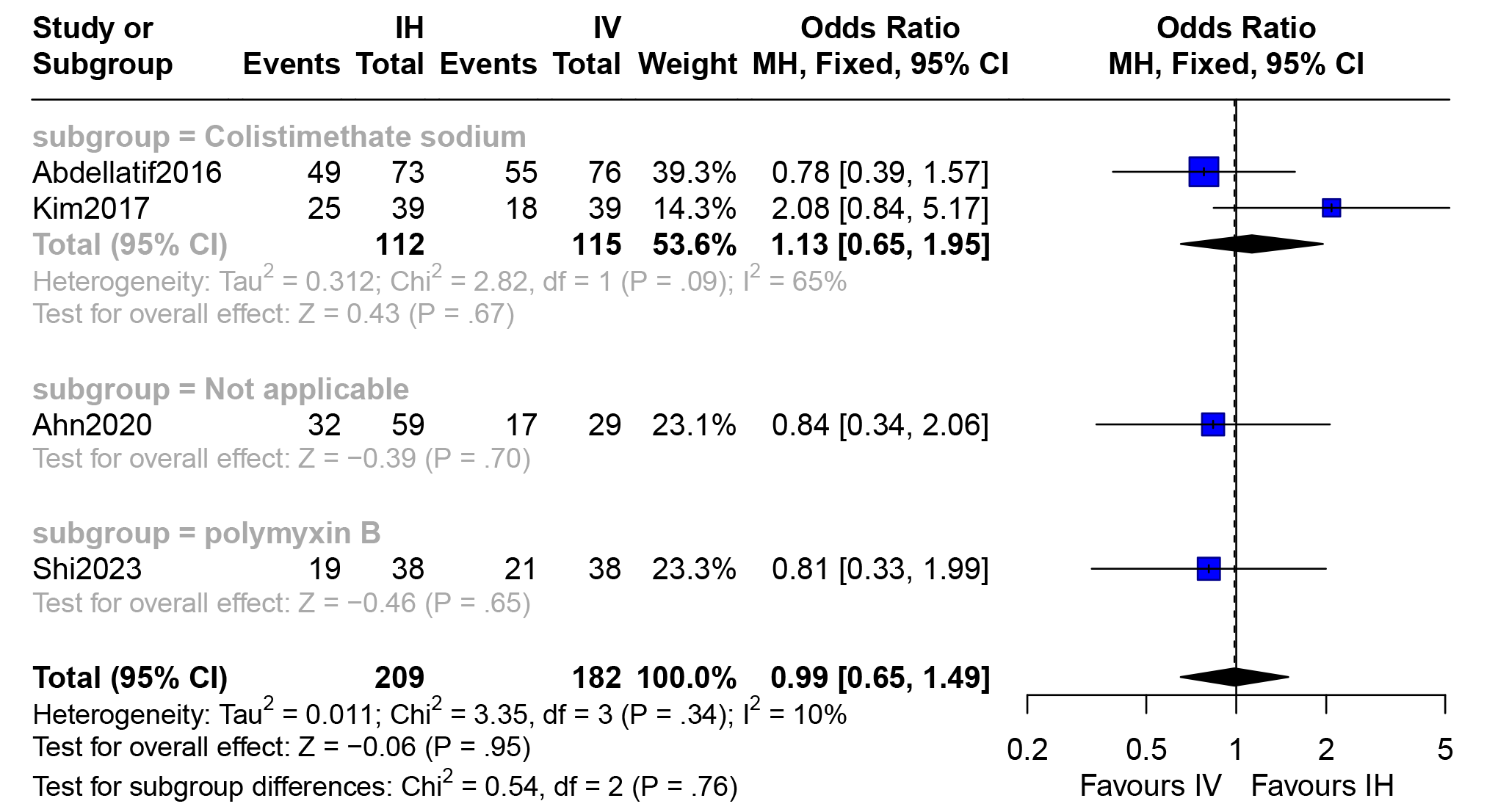


**Supplementary Fig. 33** subgroup analysis of clinical success (IH vs. IV excluded high-risk studies)

IH: inhaled polymyxins; IV: intravenous polymyxins; CI: confidence interval


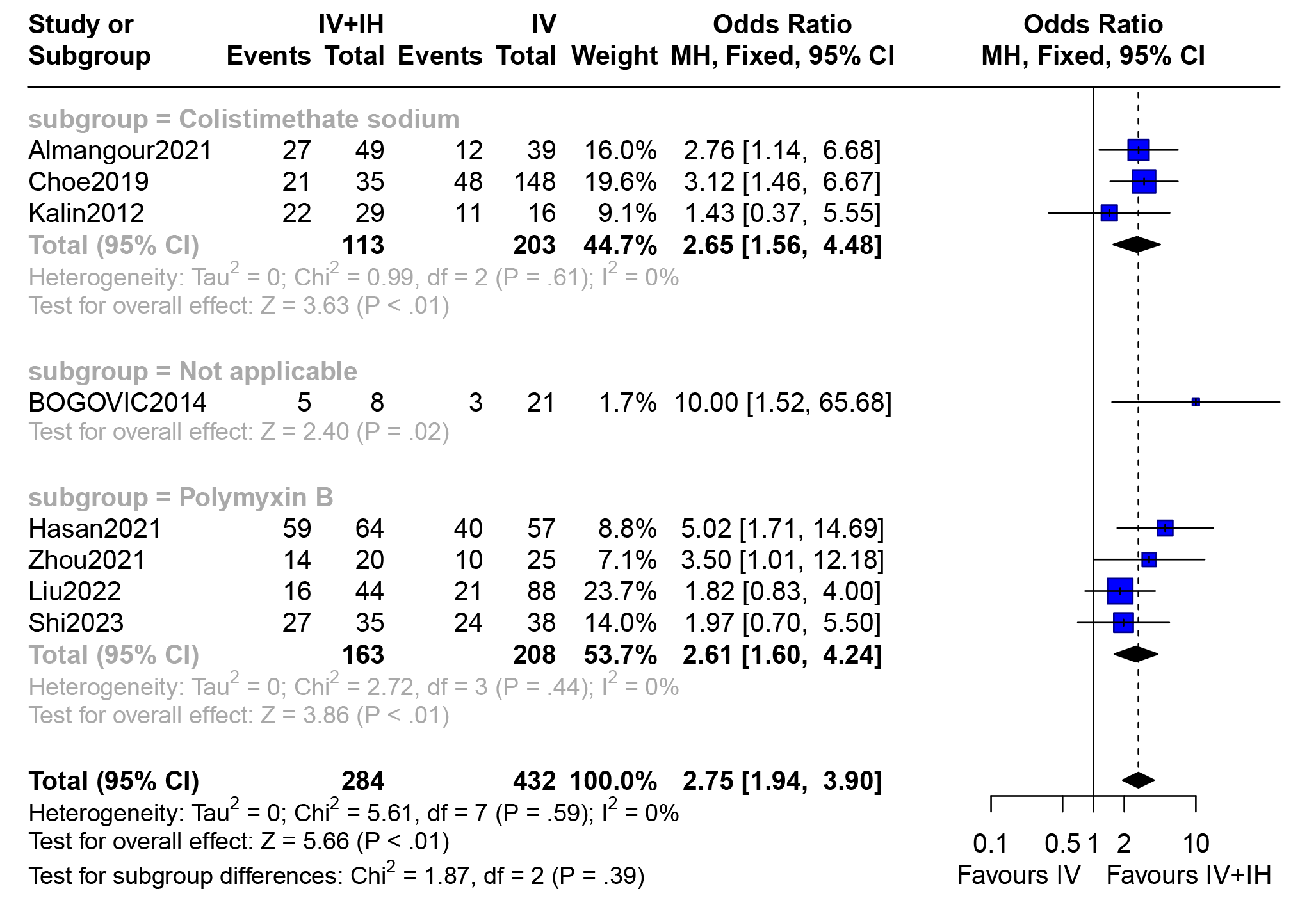


**Supplementary Fig. 34** subgroup analysis of microbial eradication rate (IV + IH vs. IV excluded high-risk studies)

IV + IH: intravenous plus inhaled polymyxins; IV: intravenous polymyxins; CI: confidence interval


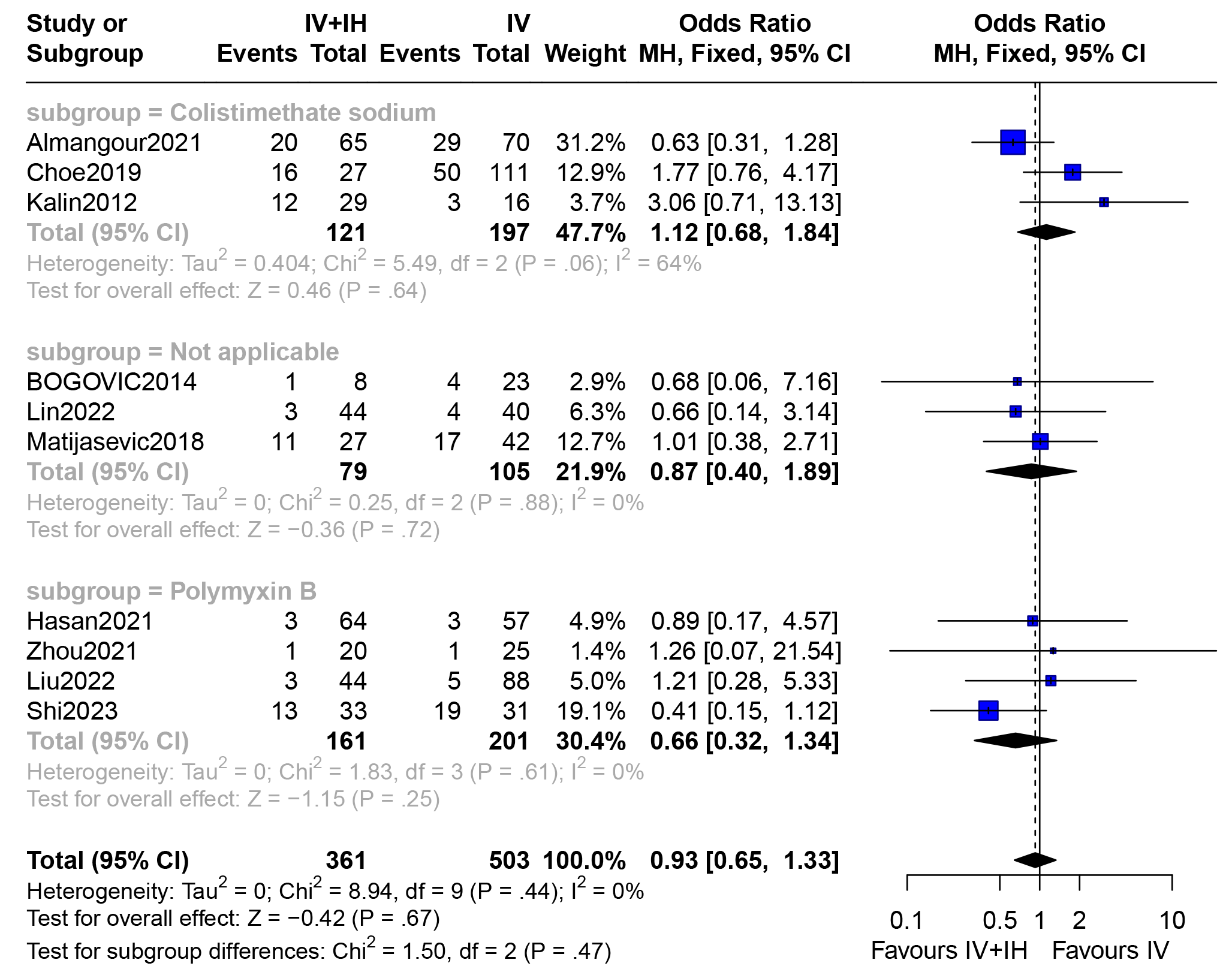


**Supplementary Fig. 35** subgroup analysis of acute kidney injury (IV + IH vs. IV excluded high-risk studies)

IV + IH: intravenous plus inhaled polymyxins; IV: intravenous polymyxins; CI: confidence interval


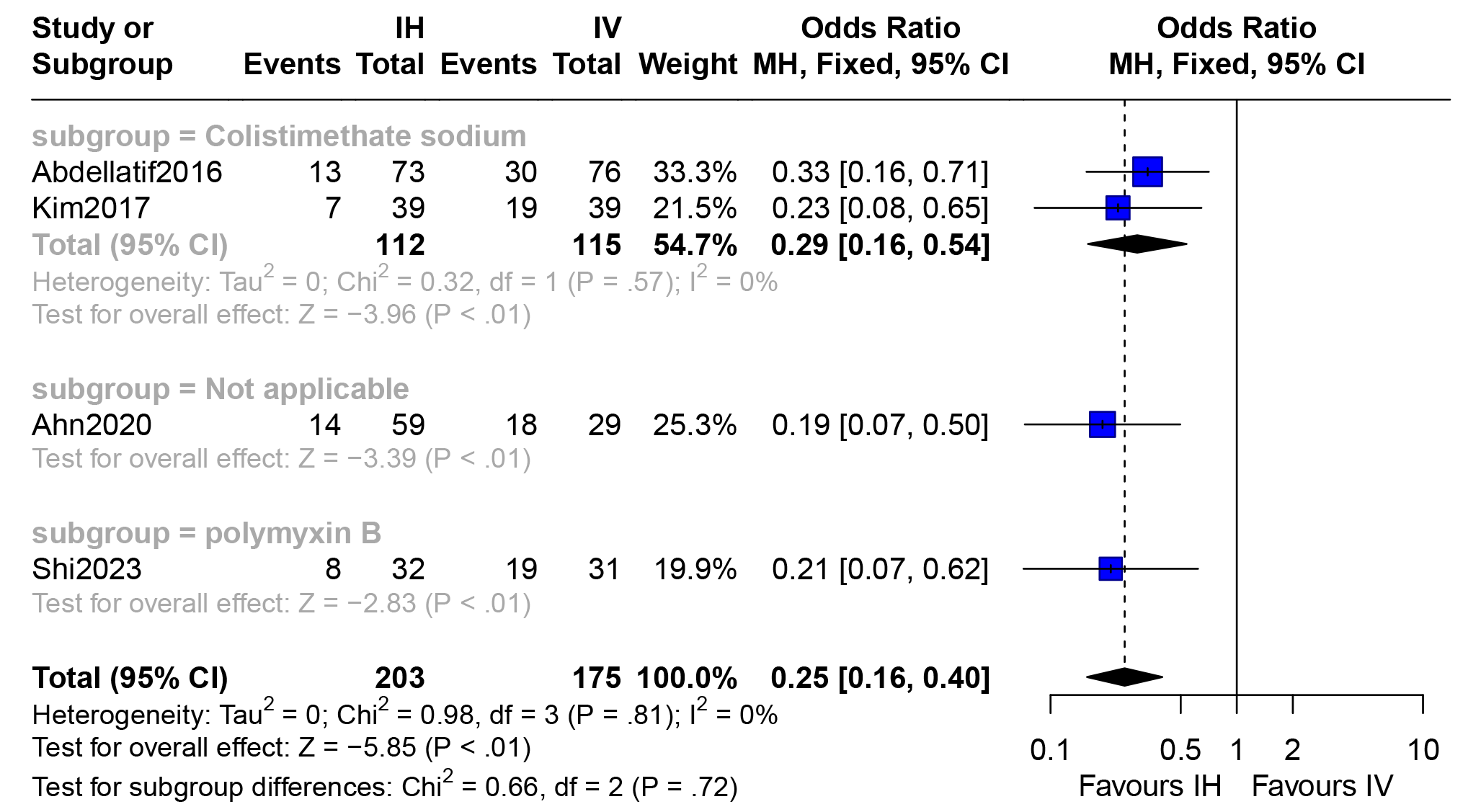


**Supplementary Fig. 36** subgroup analysis of acute kidney injury (IH vs. IV excluded high-risk studies)

IH: inhaled polymyxins; IV: intravenous polymyxins; CI: confidence interval


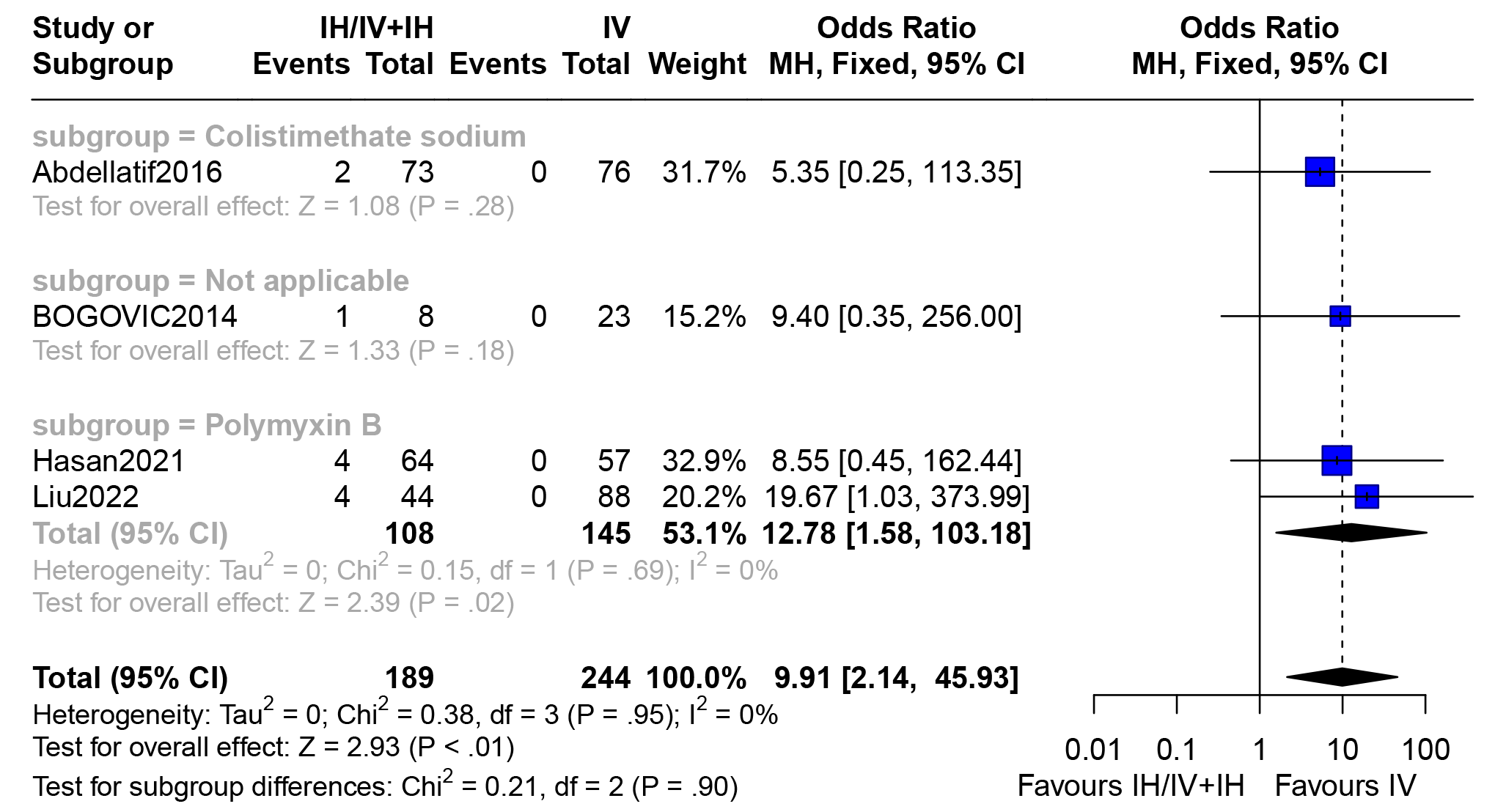


**Supplementary Fig. 37** subgroup analysis of incidence of bronchospasm (IH and IV + IH vs. IV excluded high-risk studies)

IH: inhaled polymyxins; IV + IH: intravenous plus inhaled polymyxins; IV: intravenous polymyxins; CI: confidence interval

Subgroup analyses results according to pathogen species are shown in Supplementary Fig. 38–40.


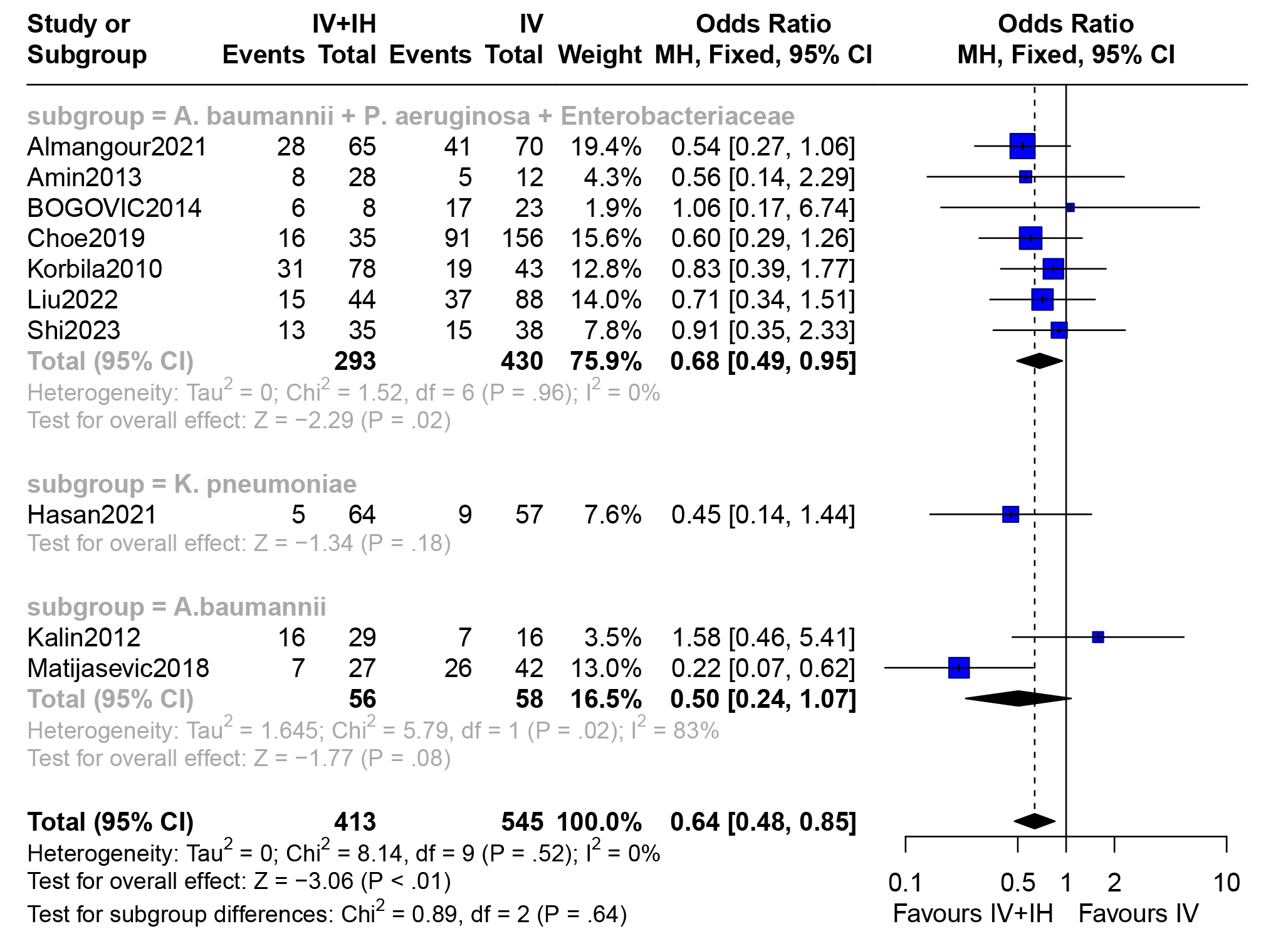


**Supplementary Fig. 38** subgroup analysis of overall mortality (IV + IH vs. IV excluded high-risk studies)

IV + IH: intravenous plus inhaled polymyxins; IV: intravenous polymyxins; CI: confidence interval


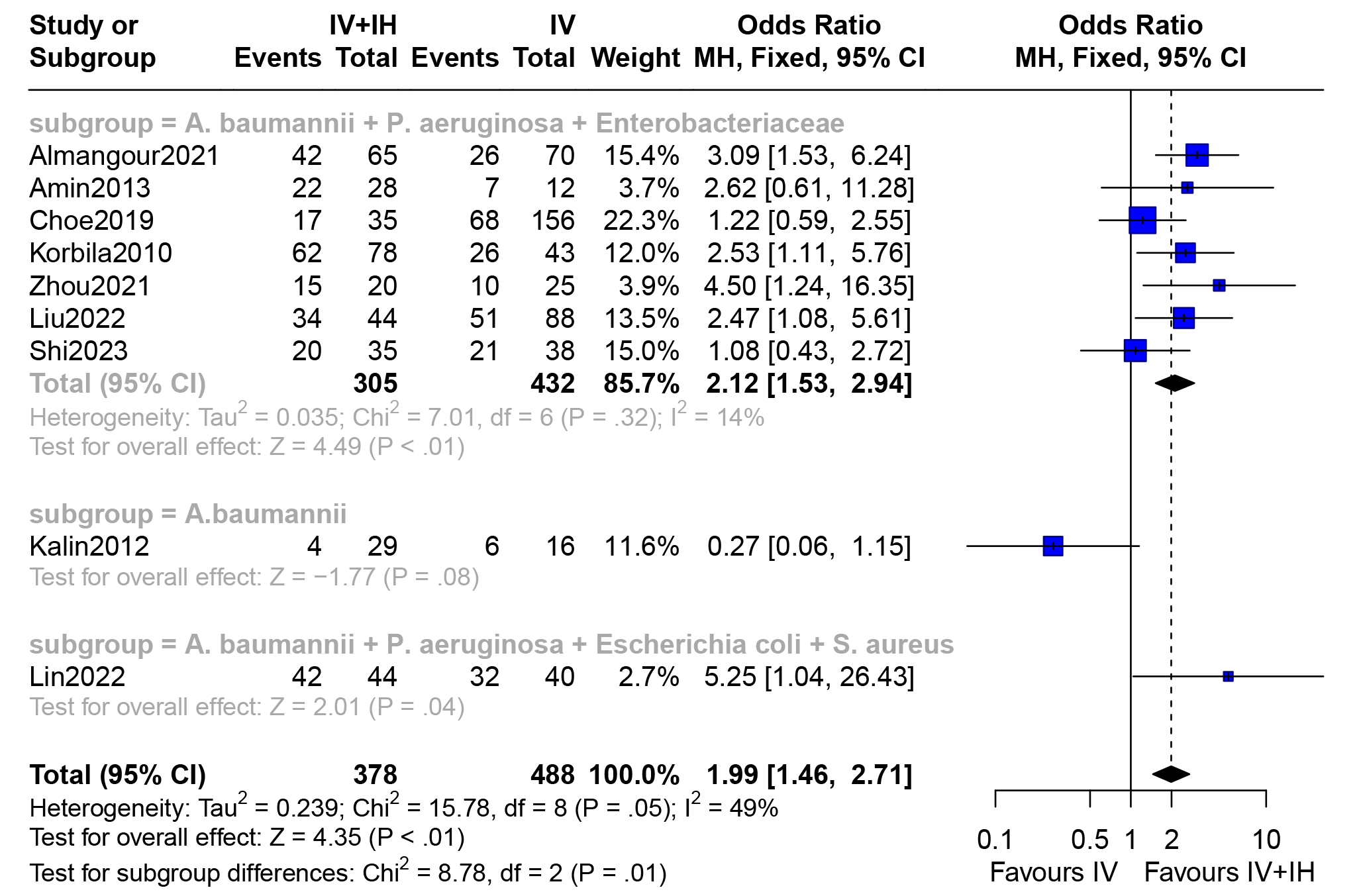


**Supplementary Fig. 39** subgroup analysis of clinical success (IV + IH vs. IV excluded high-risk studies)

IV + IH: intravenous plus inhaled polymyxins; IV: intravenous polymyxins; CI: confidence interval


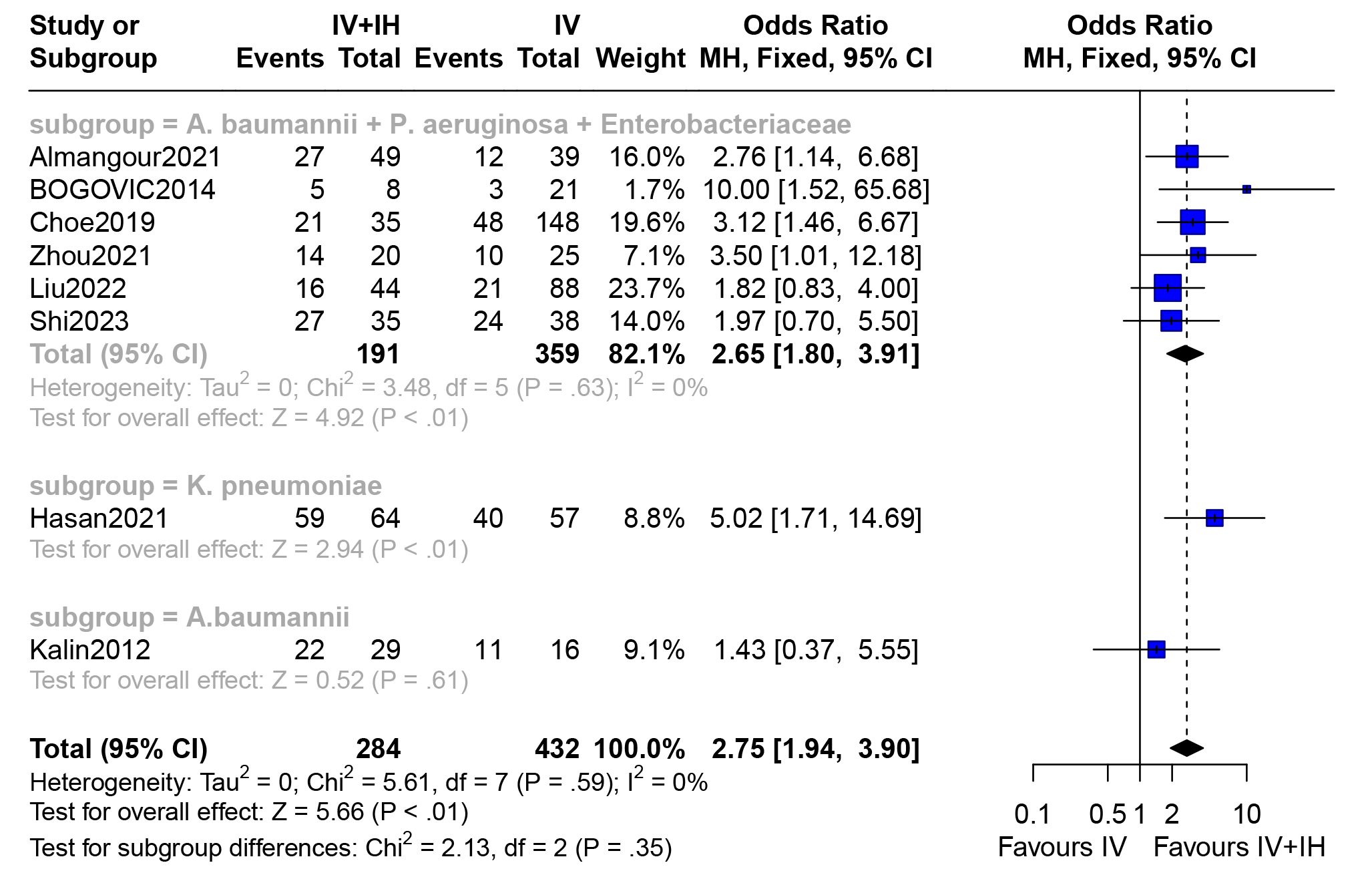


**Supplementary Fig. 40** subgroup analysis of microbial eradication rate (IV + IH vs. IV excluded high-risk studies)

IV + IH: intravenous plus inhaled polymyxins; IV: intravenous polymyxins; CI: confidence interval

Subgroup analyses results according to nebulizer type are shown in Supplementary Fig. 41–42.


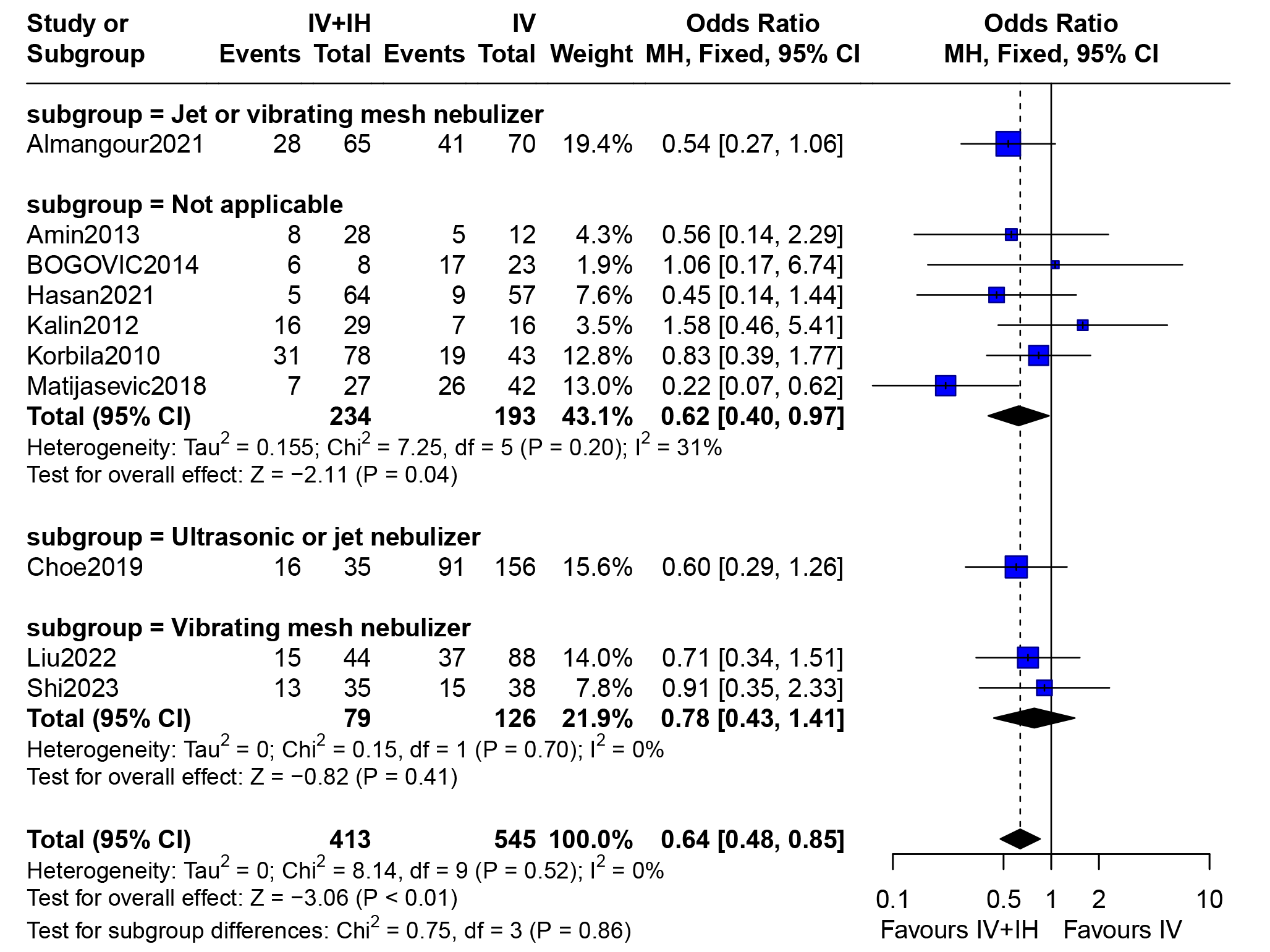


**Supplementary Fig. 41** subgroup analysis of overall mortality (IV + IH vs. IV excluded high-risk studies)

IV + IH: intravenous plus inhaled polymyxins; IV: intravenous polymyxins; CI: confidence interval


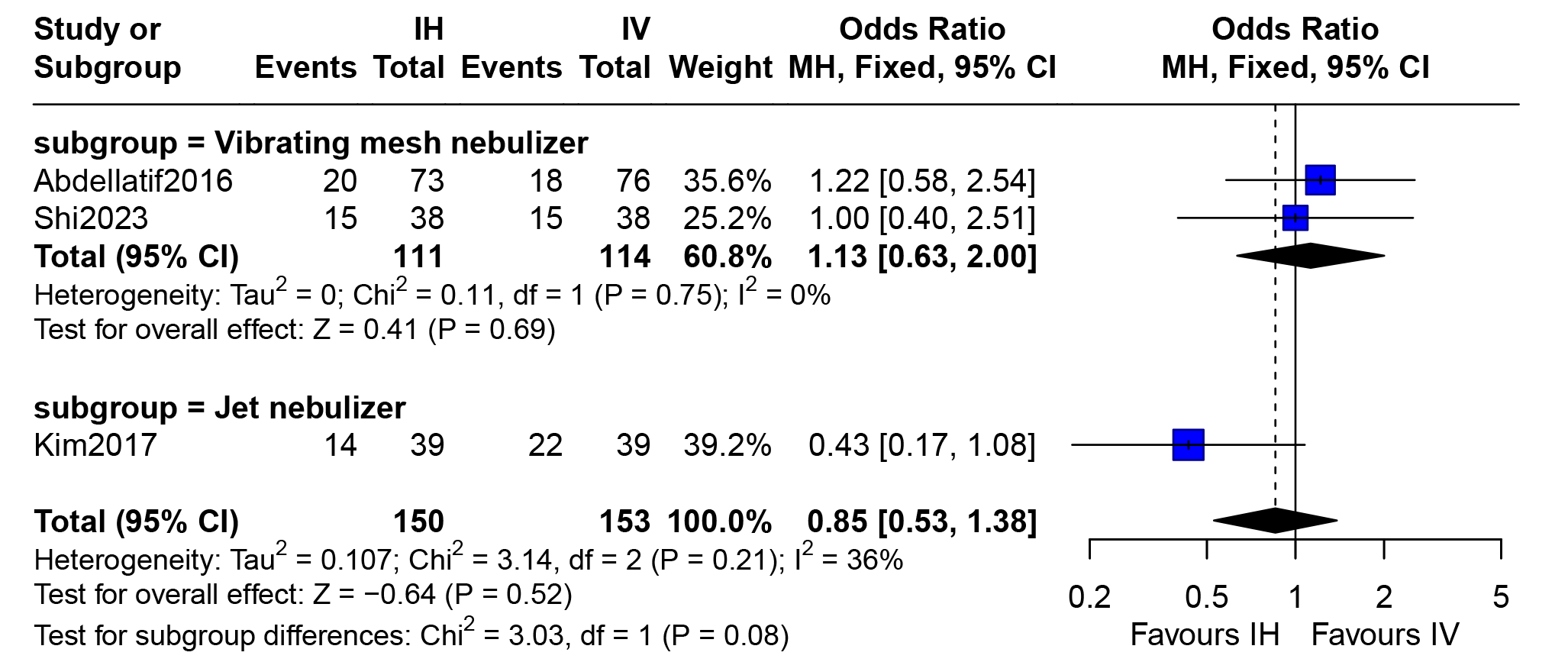


**Supplementary Fig. 42** subgroup analysis of overall mortality (IH vs. IV excluded high-risk studies)

IH: inhaled polymyxins; IV: intravenous polymyxins; CI: confidence interval

**Appendix 5:** **Convergence and density plot**

The Bayesian model shows that each Markov chain fluctuates less up and down after 100000 pre-iterations, and the iteration trajectory tends to a stable level, indicating that the model has converged to a stationary target distribution and has good convergence. Additionally, potential scale reduction factors (PSRF) converges to 1 after 100000 pre-iterations in the convergence diagnosis chart, indicating that the results between different chains tend to be equal and the model converges satisfactorily.


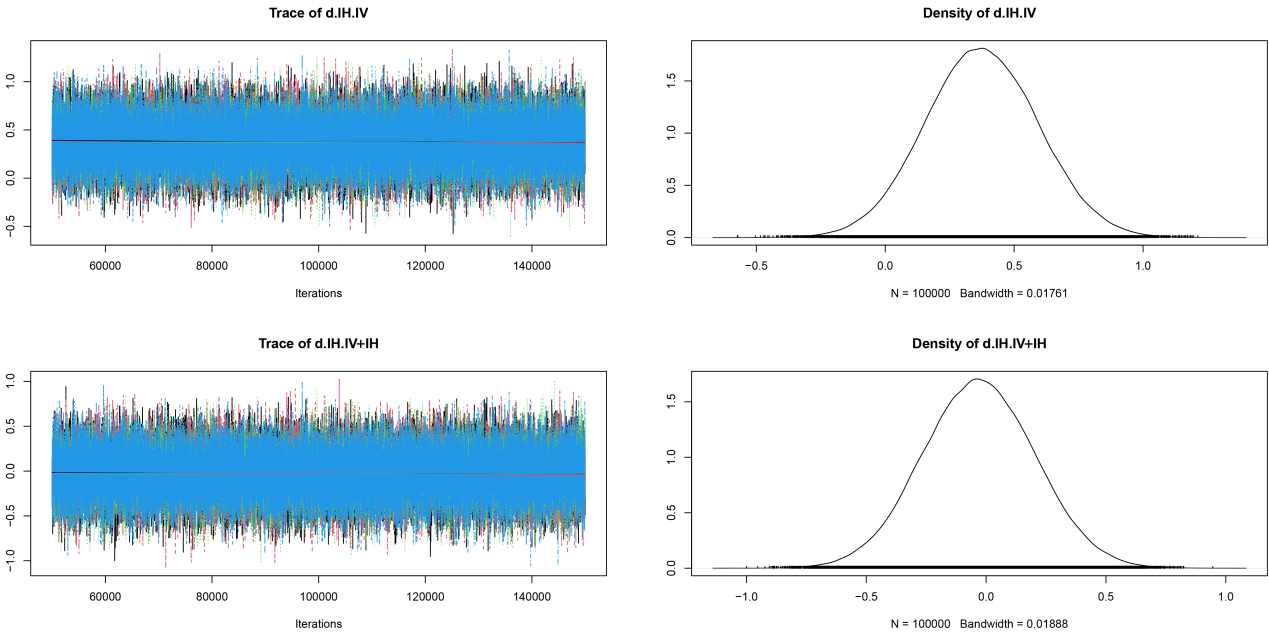


**Supplementary Fig. 43** convergence and density plot of overall mortality

IH.IV: inhaled polymyxins vs. intravenous polymyxins; IH.IV + IH: inhaled polymyxins vs. inhaled plus intravenous polymyxins


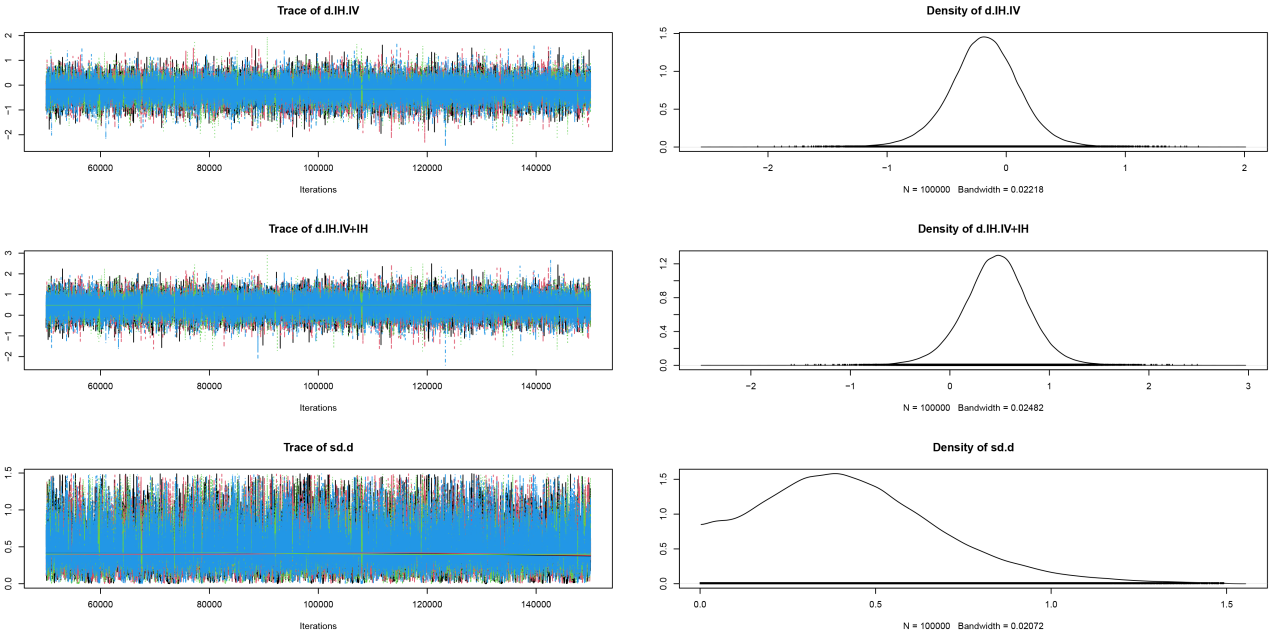


**Supplementary Fig. 44** convergence and density plot of clinical success

IH.IV: inhaled polymyxins vs. intravenous polymyxins; IH.IV + IH: inhaled polymyxins vs. inhaled plus intravenous polymyxins


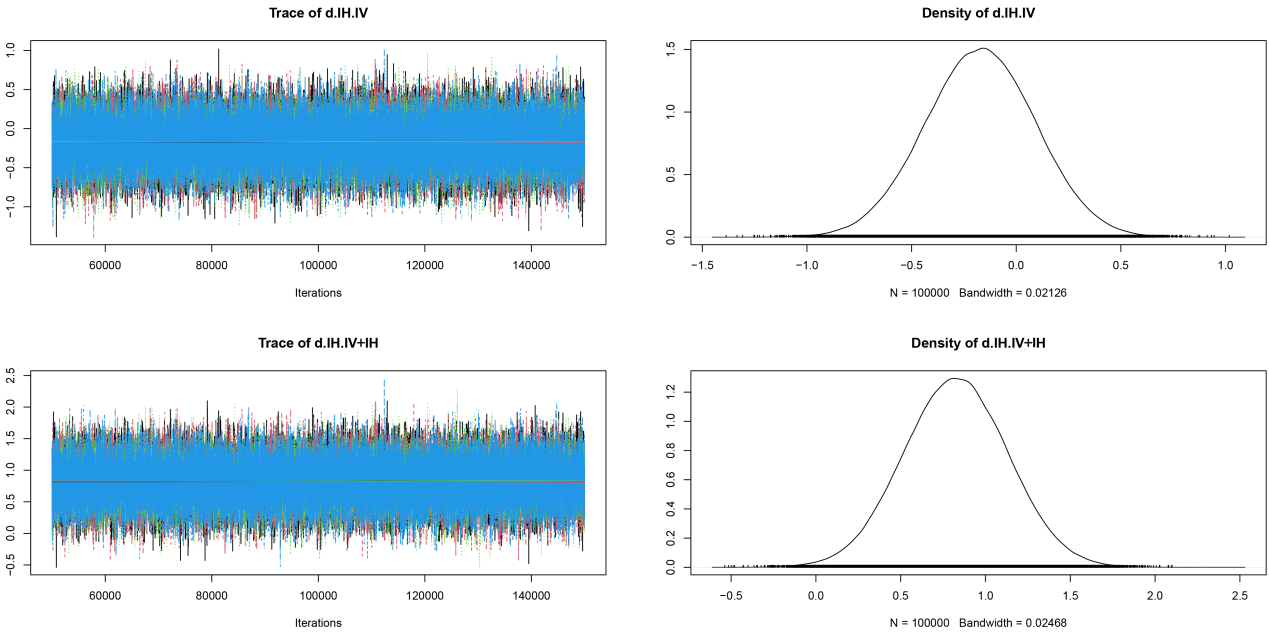


**Supplementary Fig. 45** convergence and density plot of microbial eradication rate

IH.IV: inhaled polymyxins vs. intravenous polymyxins; IH.IV + IH: inhaled polymyxins vs. inhaled plus intravenous polymyxins


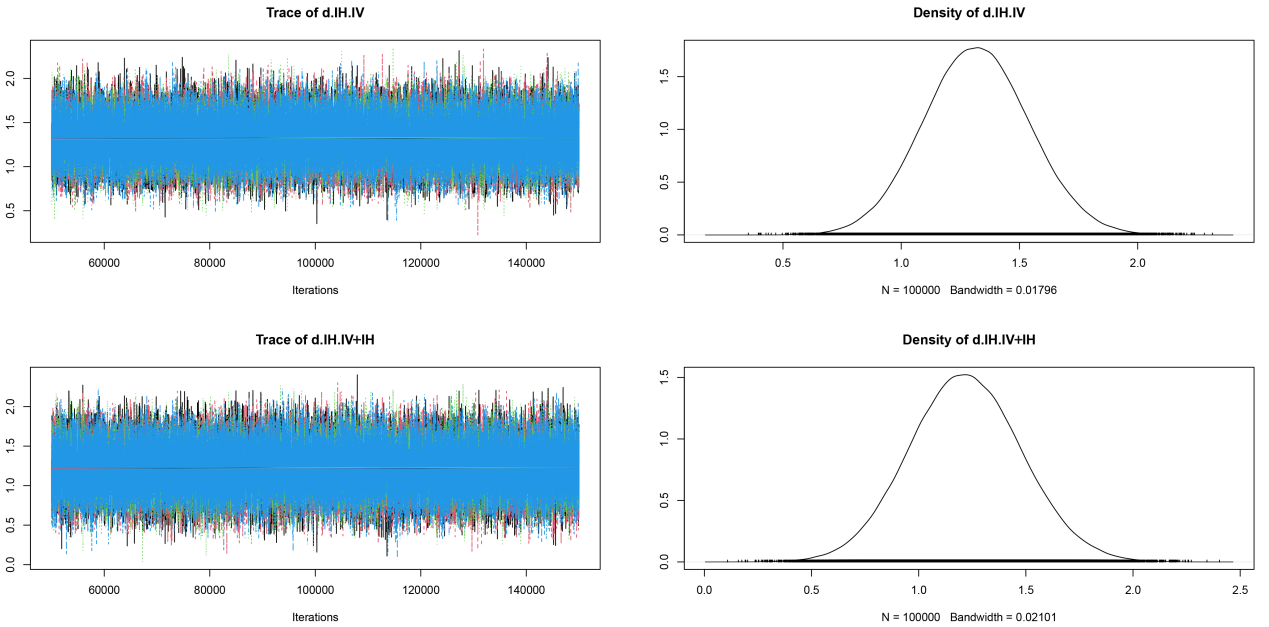


**Supplementary Fig. 46** convergence and density plot of acute kidney injury

IH.IV: inhaled polymyxins vs. intravenous polymyxins; IH.IV + IH: inhaled polymyxins vs. inhaled plus intravenous polymyxins

**Appendix 6:** **Assessment of heterogeneity, consistency and model fit**

Node-splitting analysis was used to detect the consistency of the closed loop in the evidence network. Pairwise comparisons of all split nodes showed no statistically significant difference between direct and indirect comparison evidence, indicating that there was no inconsistency in results (p > 0.05). Therefore, the network meta-analysis statistical results choose a consistency model. Except for the clinical success derived from random effects models, all other Bayesian network meta-analysis estimates are derived from fixed effects models because they are more suitable than random effects models (overall mortality, DICfixed = 44.66 versus DICrandom = 45.66; microbial eradication rate, 32.56 versus 34.23; clinical success, 51.57 versus 50.72; acute kidney injury, 42.94 versus 44.34).


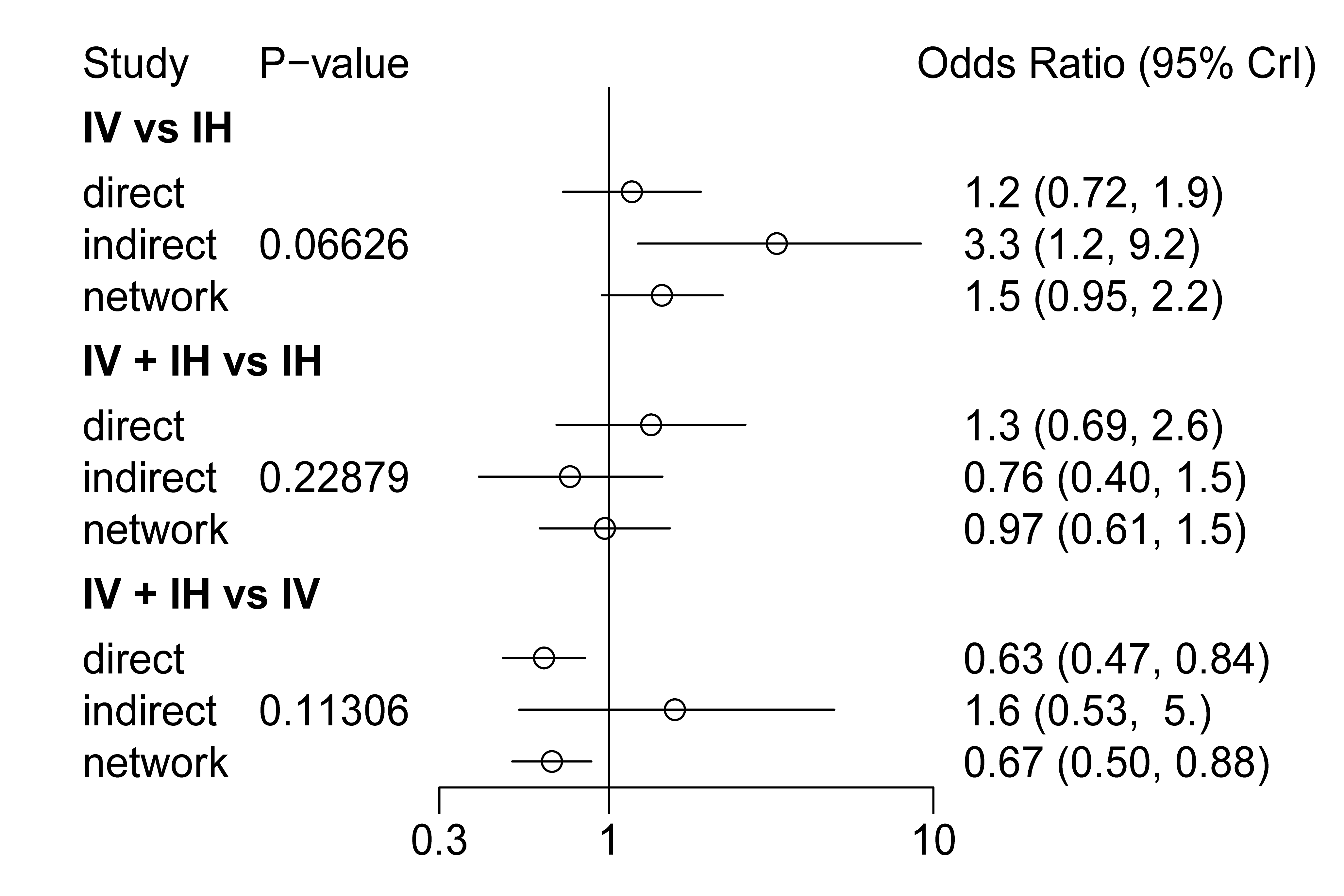


**Supplementary Fig. 47** node-splitting analysis of overall mortality

IV: intravenous polymyxins; IH: inhaled polymyxins; IV + IH: intravenous plus inhaled polymyxins; CrI: credible interval


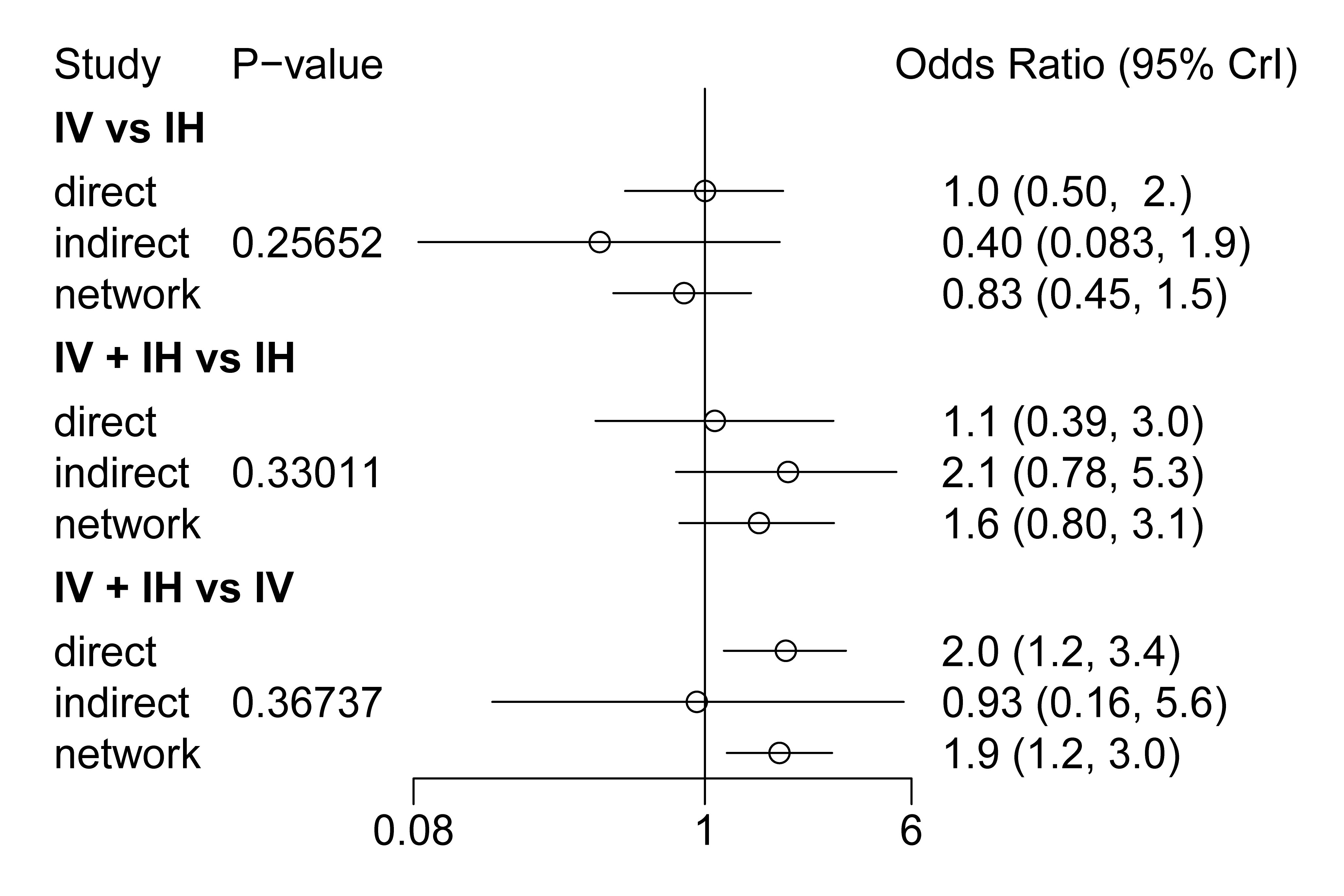


**Supplementary Fig. 48** node-splitting analysis of clinical success

IV: intravenous polymyxins; IH: inhaled polymyxins; IV + IH: intravenous plus inhaled polymyxins; CrI: credible interval


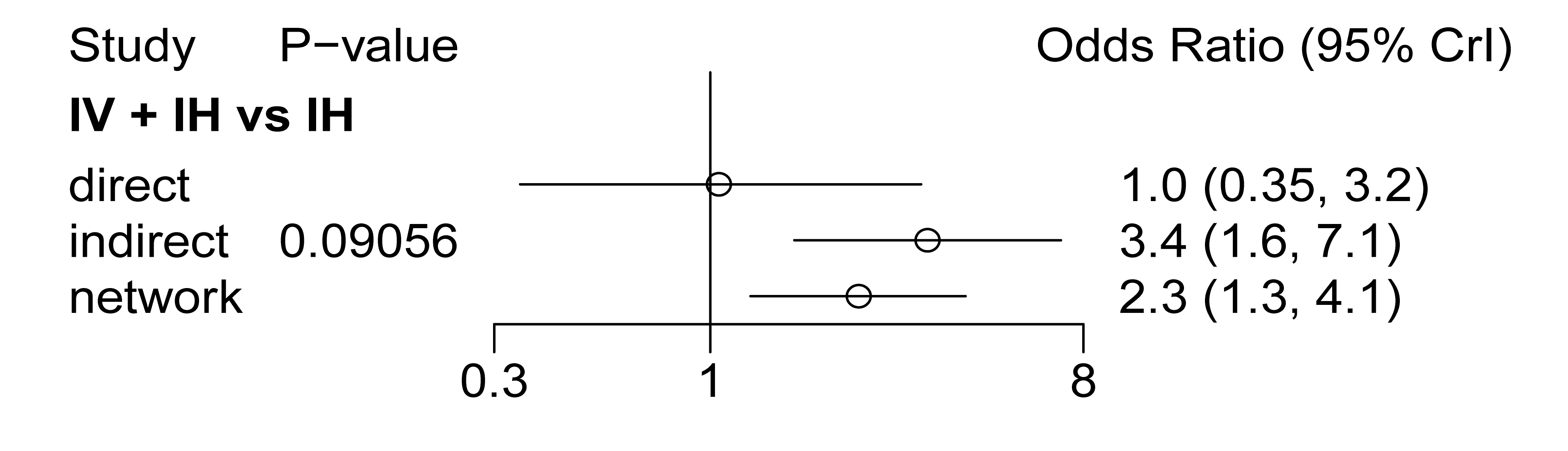


**Supplementary Fig. 49** node-splitting analysis of microbial eradication rate

IV + IH: intravenous plus inhaled polymyxins; IH: inhaled polymyxins; CrI: credible interval


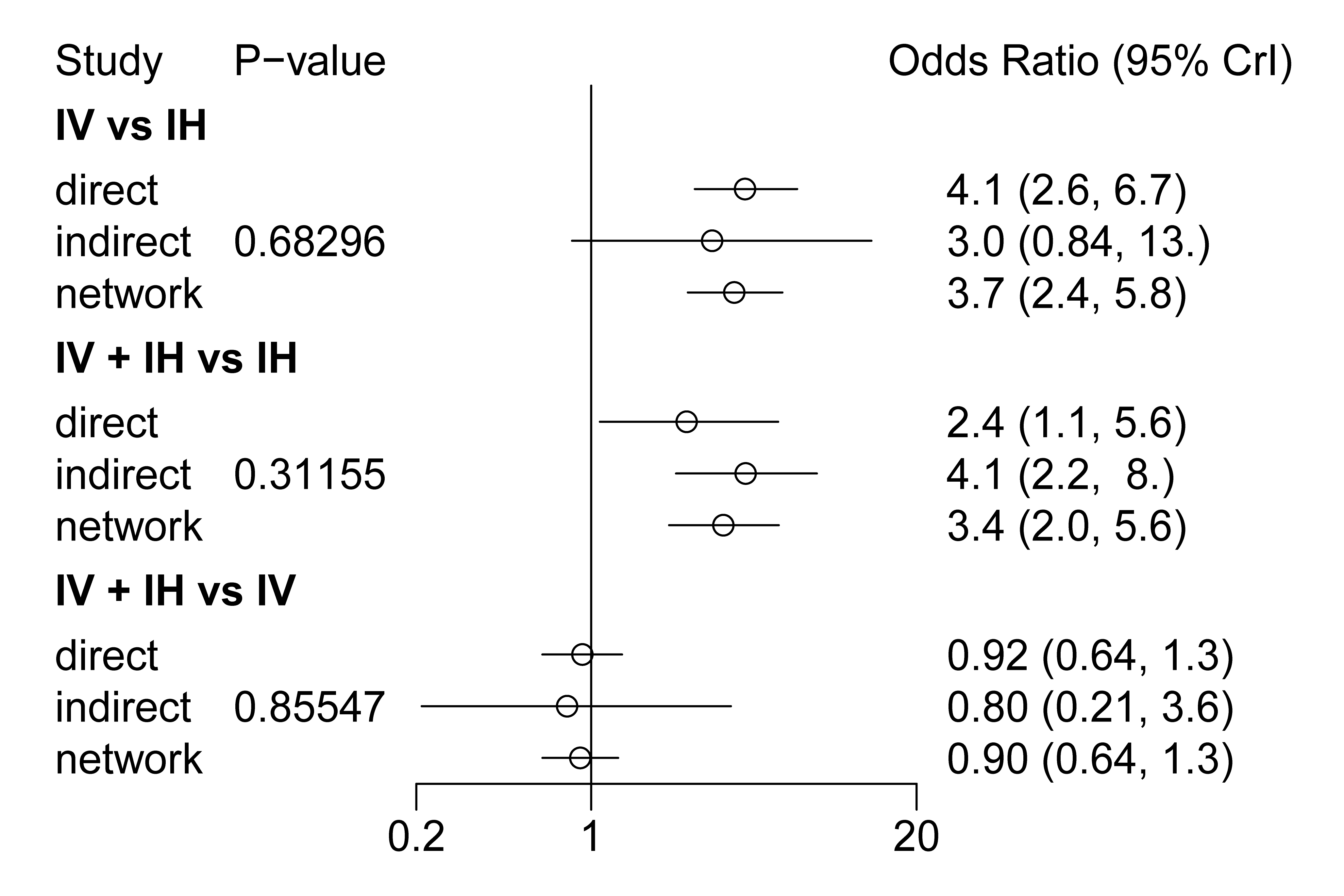


**Supplementary Fig. 50** node-splitting analysis of acute kidney injury

IV: intravenous polymyxins; IH: inhaled polymyxins; IV + IH: intravenous plus inhaled polymyxins;CrI: credible interval

**Appendix 7: Publication bias analysis: Funnel plots and Egger's test**

In the comparison between intravenous plus inhaled (IV + IH) and intravenous (IV) polymyxin-containing regimens, the statistical analysis of overall mortality and acute kidney injury included ten studies each. After assessing funnel plots and Egger’s test (overall mortality P = 0.8255; acute kidney injury P = 0.6262), no evidence of publication bias was detected. For other outcome evaluations involving < 10 studies, publication bias was not assessed to avoid misjudging accidental errors as publication bias due to the limited number of studies.


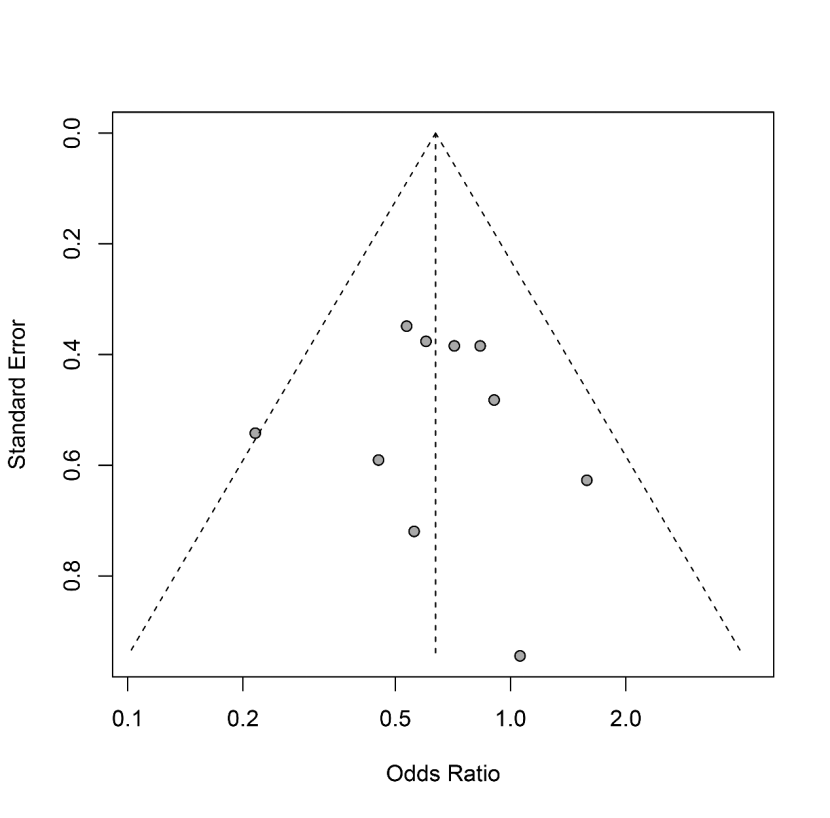


**Supplementary Fig. 51** funnel plot of overall mortality (IV + IH vs. IV excluded high-risk studies)


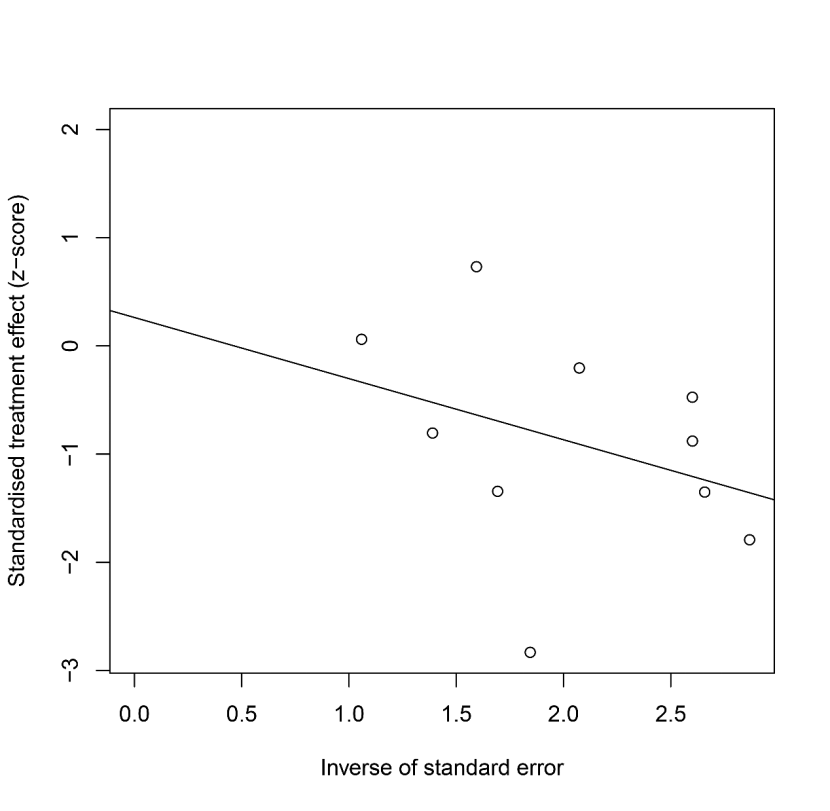


**Supplementary Fig. 52** Egger’s test of overall mortality (IV + IH vs. IV excluded high-risk studies)


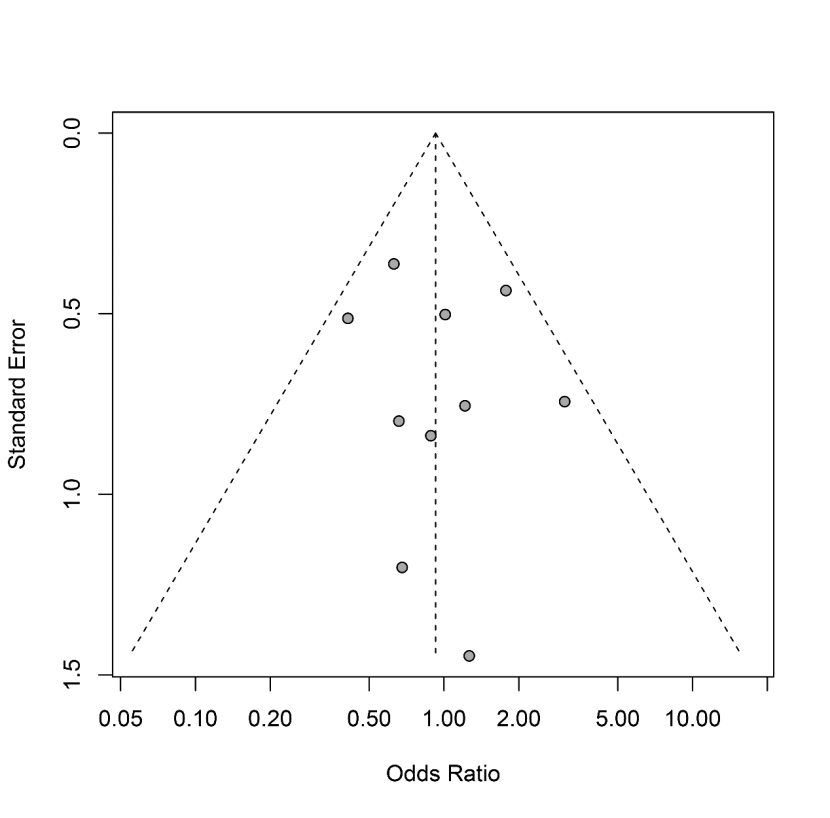


**Supplementary Fig. 53** funnel plot of acute kidney injury (IV + IH vs. IV excluded high-risk studies)


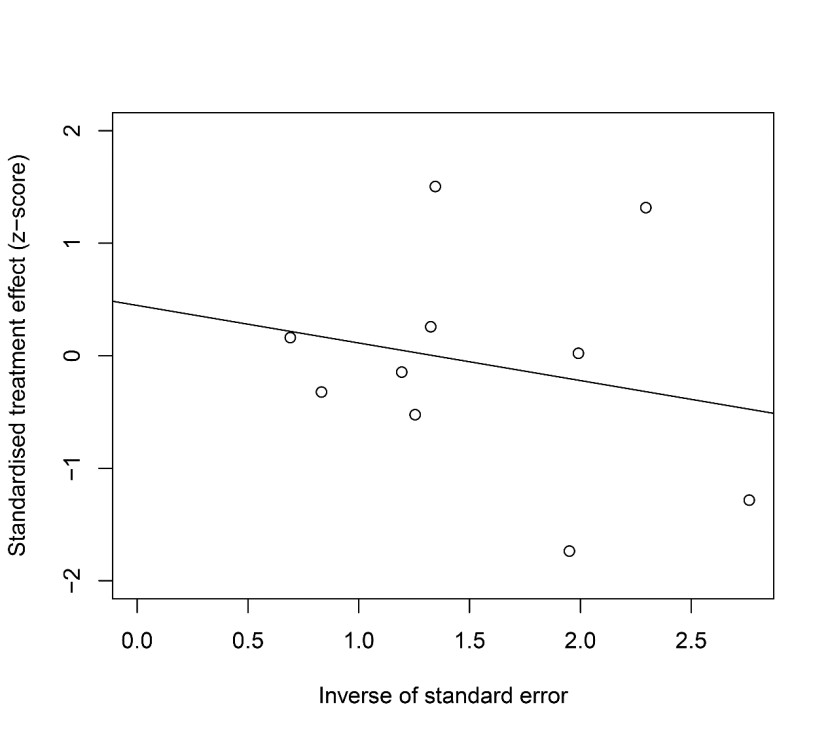


**Supplementary Fig. 54** Egger’s test of acute kidney injury (IV + IH vs. IV excluded high-risk studies)


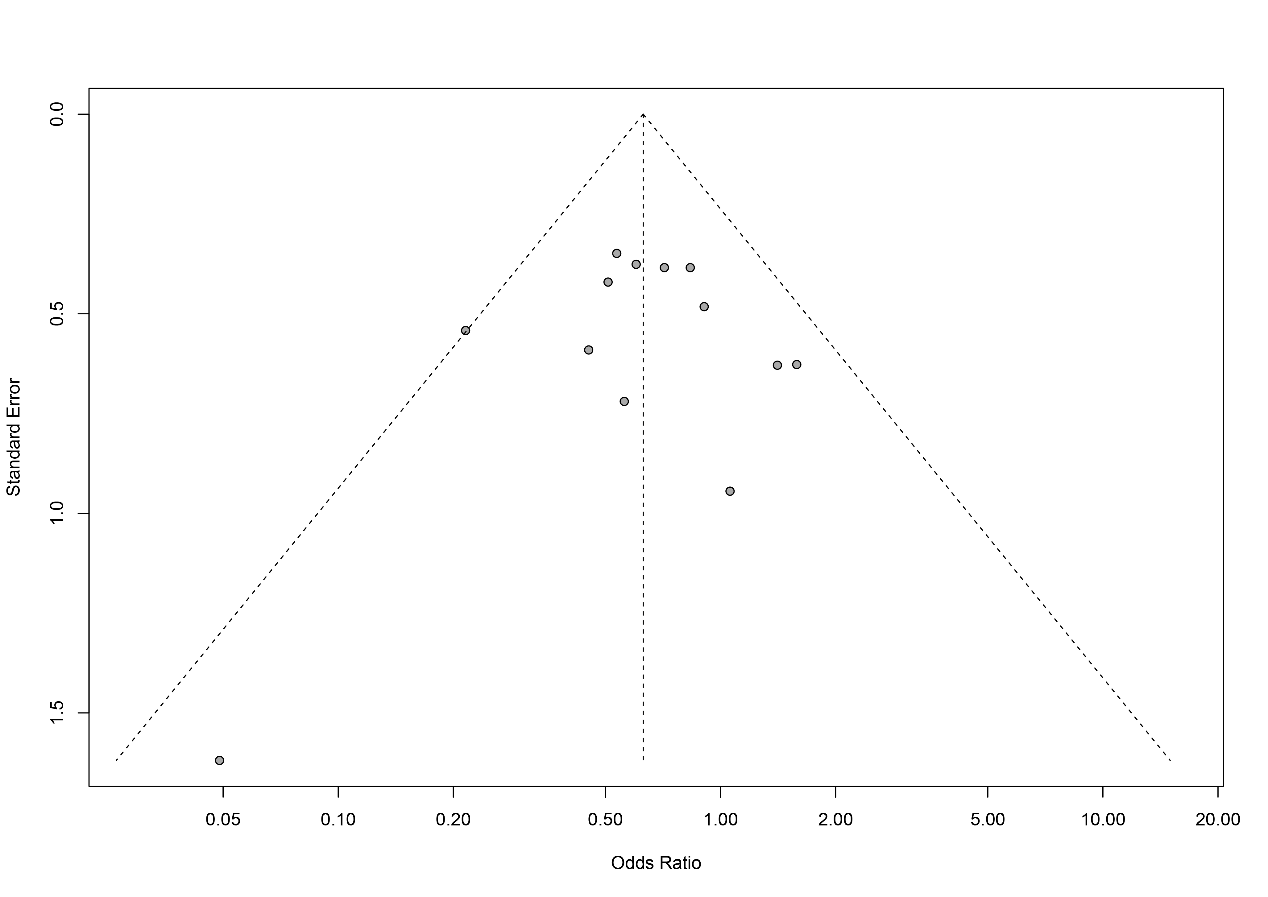


**Supplementary Fig. 55** funnel plot of overall mortality (IV + IH vs. IV)


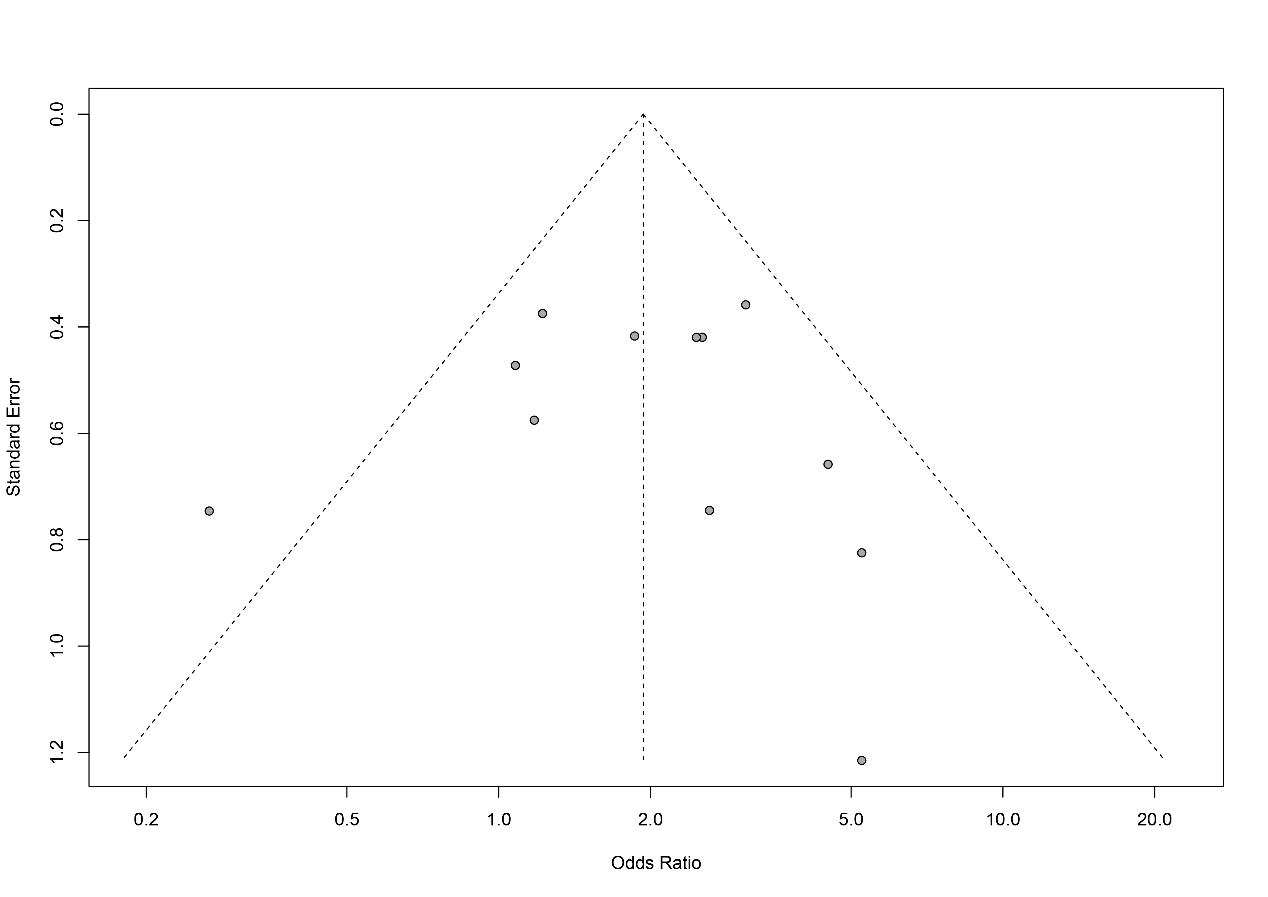


**Supplementary Fig. 56** funnel plot of clinical success (IV + IH vs. IV)


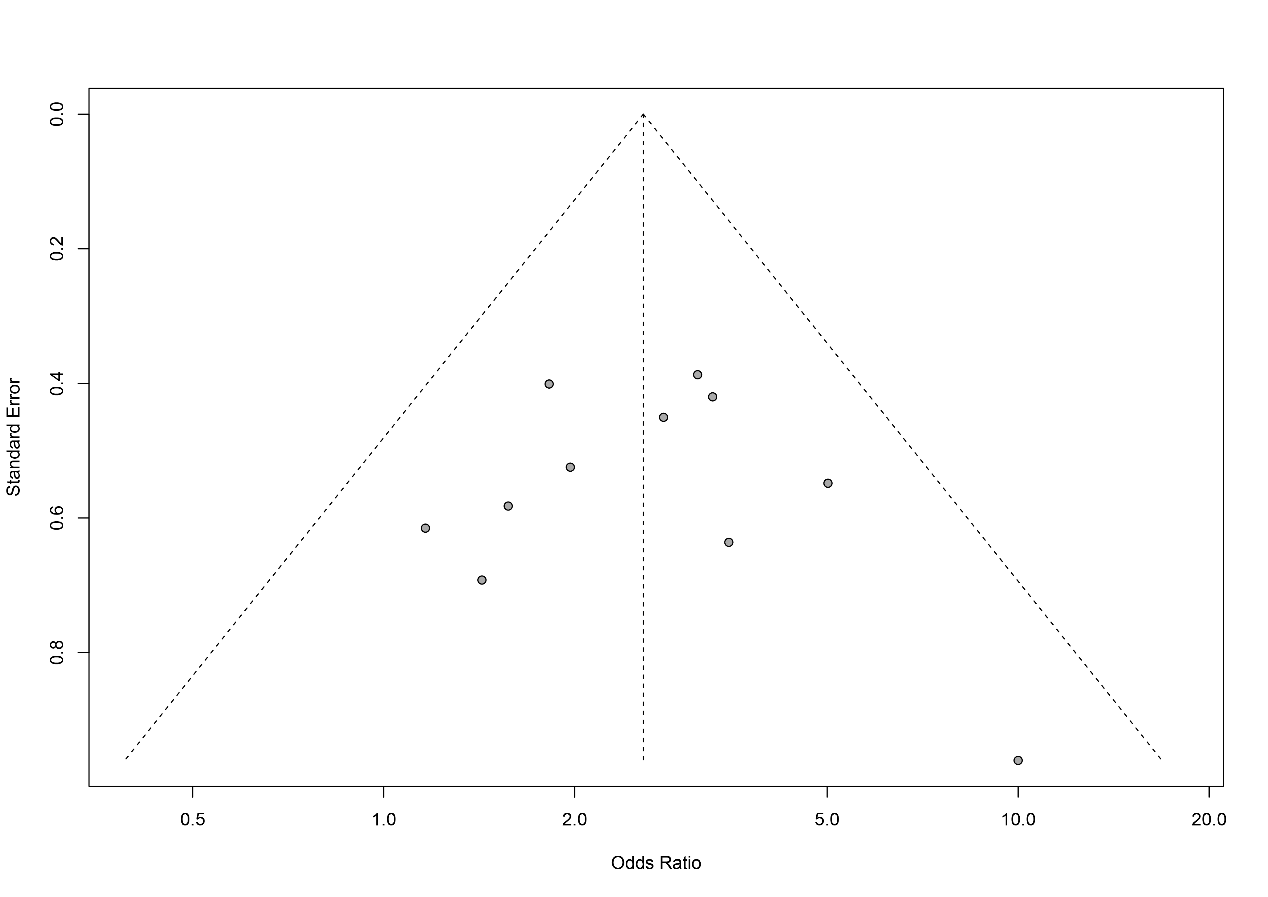


**Supplementary Fig. 57** funnel plot of microbial eradication rate (IV + IH vs. IV)


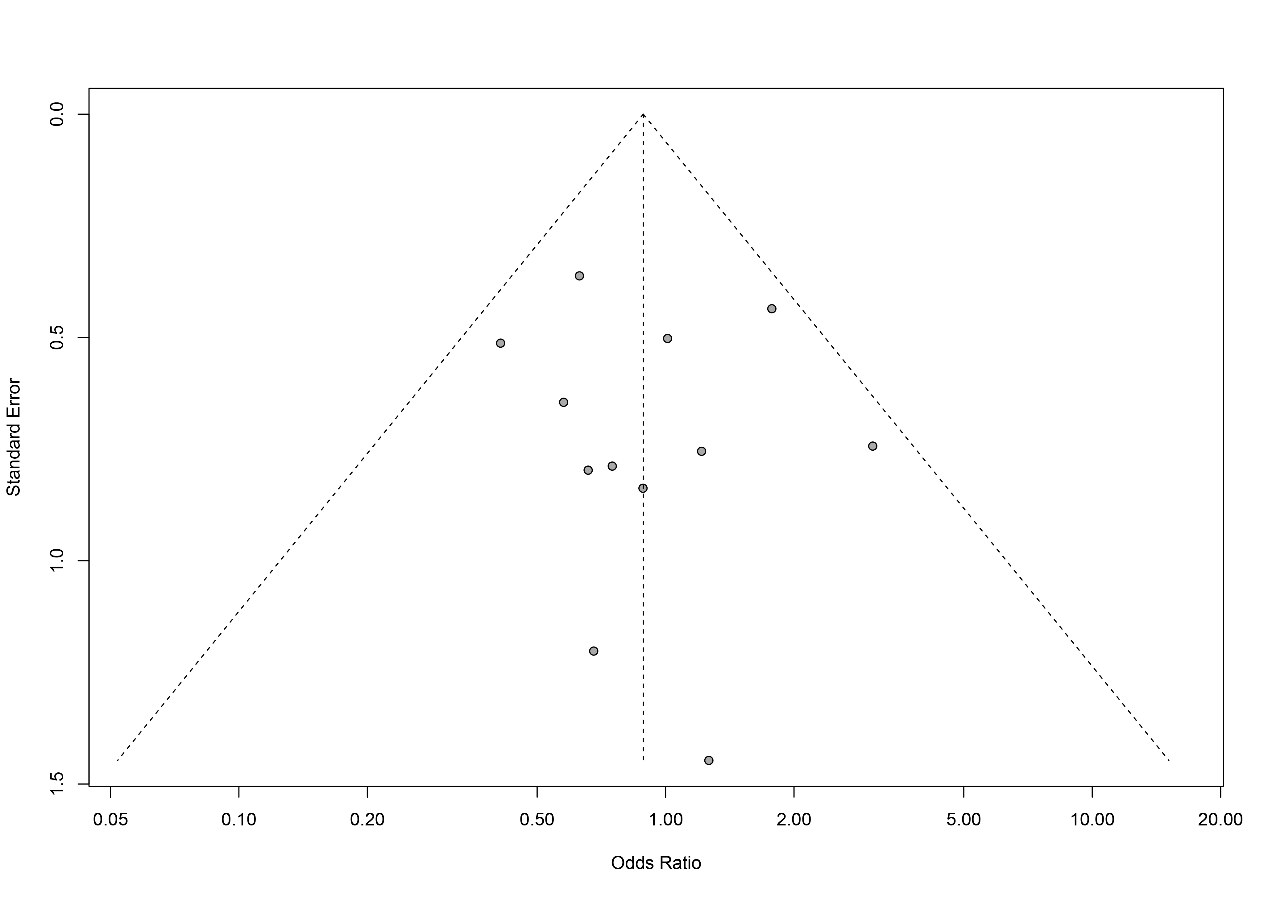


**Supplementary Fig. 58** funnel plot of acute kidney injury (IV + IH vs. IV)

**Appendix 8: Certainty of evidence**

**Supplementary Table 2** GRADE summary of findings for overall mortality

| Comparison | Number of studies | Study design | Direct estimate | | | Indirect estimate | | | Network estimate | | |
| --- | --- | --- | --- | --- | --- | --- | --- | --- | --- | --- | --- |
|  |  |  | OR | 95%CI | Certainty of evidence | OR | 95%CI | Certainty of evidence | OR | 95%CI | Certainty of evidence |
| IV + IH vs IV | 1 | RCT | 0.45 | 0.14 to 1.44 | low | No indirect | | | 0.45 | 0.14 to 1.44 | low |
| IV + IH vs IV | 9 | observational studies | 0.65 | 0.48 to 0.88 | Very low | 0.89 | 0.32 to 2.50 | Very low | 0.66 | 0.49 to 0.88 | Very low |
| IV vs IH | 1 | RCT | 0.82 | 0.39 to 1.72 | low | No indirect | | | 0.82 | 0.39 to 1.72 | low |
| IV vs IH | 2 | observational studies | 1.53 | 0.81 to 2.90 | Very low | 2.30 | 1.00 to 5.30 | Very low | 1.9 | 1.1 to 3.3 | Very low |
| IV + IH vs IH | 0 | RCT | No direct | | | 0.36 | 0.083 to 1.40 | low | 0.36 | 0.083 to 1.40 | low |
| IV + IH vs IH | 2 | observational studies | 1.34 | 0.69 to 2.59 | Very low | 1.10 | 0.50 to 2.30 | Very low | 1.3 | 0.74 to 2.2 | Very low |

IV: intravenous polymyxins; IH: inhaled polymyxins; IV + IH: intravenous plus inhaled polymyxins; CI: confidence interval; OR: odds ratio

**Supplementary Table 3** GRADE summary of findings for clinical success

| Comparison | Number of studies | Study design | Direct estimate | | | Indirect estimate | | | Network estimate | | |
| --- | --- | --- | --- | --- | --- | --- | --- | --- | --- | --- | --- |
|  |  |  | OR | 95%CI | Certainty of evidence | OR | 95%CI | Certainty of evidence | OR | 95%CI | Certainty of evidence |
| IV + IH vs IV | 0 | RCT | No direct | | | No indirect | | |  | | |
| IV + IH vs IV | 9 | observational studies | 1.99 | 1.46 to 2.71 | Very low | 1.00 | 0.19 to 5.90 | Very low | 2 | 1.2 to 3.1 | Very low |
| IV vs IH | 1 | RCT | 1.28 | 0.64 to 2.59 | low | No indirect | | | 1.28 | 0.64 to 2.59 | low |
| IV vs IH | 3 | observational studies | 0.89 | 0.53 to 1.49 | Very low | 0.43 | 0.11 to 1.70 | Very low | 0.72 | 0.35 to 1.5 | Very low |
| IV + IH vs IH | 0 | RCT | No direct | | | No indirect | | |  | | |
| IV + IH vs IH | 2 | observational studies | 1.10 | 0.56 to 2.16 | Very low | 1.90 | 0.65 to 5.50 | Very low | 1.4 | 0.66 to 3.00 | Very low |

IV: intravenous polymyxins; IH: inhaled polymyxins; IV + IH: intravenous plus inhaled polymyxins; CI: confidence interval; OR: odds ratio

**Supplementary Table 4** GRADE summary of findings for microbial eradication rate

| Comparison | Number of studies | Study design | Direct estimate | | | Indirect estimate | | | Network estimate | | |
| --- | --- | --- | --- | --- | --- | --- | --- | --- | --- | --- | --- |
|  |  |  | OR | 95%CI | Certainty of evidence | OR | 95%CI | Certainty of evidence | OR | 95%CI | Certainty of evidence |
| IV + IH vs IV | 1 | RCT | 5.02 | 1.71 to 14.69 | Moderate | No indirect | | | 5.02 | 1.71 to 14.69 | Moderate |
| IV + IH vs IV | 7 | observational studies | 2.53 | 1.74 to 3.67 | Very low | No indirect | | | 2.53 | 1.74 to 3.67 | Very low |
| IV vs IH | 0 | RCT | No direct | | | No indirect | | |  | | |
| IV vs IH | 3 | observational studies | 0.92 | 0.54 to 1.56 | Very low | No indirect | | | 0.92 | 0.54 to 1.56 | Very low |
| IV + IH vs IH | 0 | RCT | No direct | | | No indirect | | |  | | |
| IV + IH vs IH | 1 | observational studies | 1.05 | 0.35 to 3.11 | Very low | 2.50 | 1.30 to 4.80 | Very low | 2.1 | 1.2 to 3.9 | Very low |

IV: intravenous polymyxins; IH: inhaled polymyxins; IV + IH: intravenous plus inhaled polymyxins; CI: confidence interval; OR: odds ratio

**Supplementary Table 5** GRADE summary of findings for acute kidney injury

| Comparison | Number of studies | Study design | Direct estimate | | | Indirect estimate | | | Network estimate | | |
| --- | --- | --- | --- | --- | --- | --- | --- | --- | --- | --- | --- |
|  |  |  | OR | 95%CI | Certainty of evidence | OR | 95%CI | Certainty of evidence | OR | 95%CI | Certainty of evidence |
| IV + IH vs IV | 1 | RCT | 0.89 | 0.17 to 4.57 | Very low | No indirect | | | 0.89 | 0.17 to 4.57 | Very low |
| IV + IH vs IV | 9 | observational studies | 0.93 | 0.64 to 1.34 | Very low | 0.49 | 0.16 to 1.5 | Very low | 0.89 | 0.62 to 1.3 | Very low |
| IV vs IH | 1 | RCT | 3.01 | 1.41 to 6.41 | High | No indirect | | | 3.01 | 1.41 to 6.41 | High |
| IV vs IH | 3 | observational studies | 4.78 | 2.65 to 8.62 | Moderate | 2.50 | 0.91 to 7.00 | Very low | 4.1 | 2.5 to 7.1 | Moderate |
| IV + IH vs IH | 0 | RCT | No direct | | | 2.70 | 0.39 to 19.00 | Very low | 2.70 | 0.39 to 19.00 | Very low |
| IV + IH vs IH | 2 | observational studies | 2.35 | 1.05 to 5.28 | Very low | 4.70 | 2.20 to 9.90 | Very low | 3.7 | 2.1 to 6.6 | Very low |

IV: intravenous polymyxins; IH: inhaled polymyxins; IV + IH: intravenous plus inhaled polymyxins; CI: confidence interval; OR: odds ratio

The certainty of evidence was evaluated using outcomes of the primary analysis, which was based on trials after the exclusion of studies adjudicated as having an overall high risk of bias.

**Appendix 9: Definition of clinical success**

| Study | Definition of clinical success |
| --- | --- |
| Abdellatif,2016 [7] | Therapeutic efficacy was assessed by the cure of VAP at the end of colistin therapy (day 14). The cure of VAP was defined as the resolution of clinical and biological signs of infection, i.e. a CPIS less than 6 and bacteriological eradication. |
| Ahn,2020 [8] | Treatment success was defined as improvement in clinical symptoms and negative bacterial cultures after polymyxins treatment. Or when there is no microbiologic result or bacterial conversion but clinical improvement. Clinical improvement was defined as discharge after drug withdrawal due to improvement of infection, and negative bacterial culture was defined as two consecutive negative bacterial culture results after the start of polymyxins administration. |
| Almangour,2021 [9] | The definition of clinical cure was based on the resolution of fever; reduction in suctioning requirements; decrease or lack of progression of radiographic abnormalities on chest X-ray; improvement or normalization of arterial blood gases; and improvement or return to baseline of WBCs, CRP and PCT. |
| Amin,2013 [10] | Cure is defined as the resolution of presenting symptoms and signs of the infection by the end of colistin treatment. |
| Choe,2019 [11] | Clinical cure was based on the improvement of all signs and symptoms associated with pneumonia. |
| Doshi,2013 [2] | Clinical cure was defined as resolution of initial signs and symptoms of infection including normalization of white blood cell count and temperature by the end of colistin therapy. |
| Jang,2017 [3] | Clinical effective was defined as clinical cure (resolution of the infectious signs and symptoms after colistin treatment) or improvement (partial resolution of the infectious signs and symptoms during colistin treatment). |
| Kalin,2012 [12] | At the end of the COL therapy, if the symptoms and signs of VAP had resolved without antimicrobial maintenance therapy, this was defined as clinical success. |
| Kim,2017 [13] | Clinical cure was calculated by clinical failure (persistence or worsening of signs or symptoms of pneumonia and lack of improvement of radiologic pulmonary infiltrates). |
| Korbila,2010 [14] | The resolution of the infection was defined by the normalization of body temperature and tracheal secretions, together with a return to baseline of the white blood cell count and the C-reactive protein level, and the improvement in chest X-ray appearances, by the end of colistin treatment. |
| Zhou,2021 [15] | Clinical effective was defined as clinical cure (the clinical symptoms, signs, etiology and laboratory test results of the patient showed normal after treatment) or improvement (the clinical symptoms, signs, etiology and laboratory test results of patients were improved after treatment). |
| Lin,2022 [16] | Total effective was defined as markedly effective (the condition has improved significantly, and one of the pathogenic bacteria cultures, laboratory indicators and clinical signs has not returned to normal) or effective (the condition has improved, but the pathogenic bacteria culture, laboratory indicators and clinical signs are not significantly improved). |
| Liu,2022 [17] | Favorable clinical outcomes included clinical cure (i.e., the disappearance of infection-related symptoms and signs by the end of polymyxin B treatment) or clinical improvement (i.e., improved infection-related symptoms and signs by the end of polymyxin B treatment compared with before polymyxin B treatment). |
| Naesens,2011 [5] | A favourable clinical response was defined as a resolution of presenting symptoms and signs at the end of the treatment. |
| Zheng,2019 [6] | Clinical resolution of pneumonia or clinical success at the end of treatment was defined as (1) decreased pulmonary infiltrate, and (2) survival with stationary findings on chest radiographs and defervescence. |
| Wu,2023 [18] | Cure of VAP was defined as resolution of clinical and biological signs of infection, CPIS less than 6, and negative culture of lower respiratory tract specimens (if available). |
| Shi,2023 [19] | Clinical effective was defined as cure (relief of signs or symptoms of pneumonia, improvement in chest imaging, and discontinuation of antibiotic therapy) or improvement (significant improvement in signs and symptoms of pneumonia, improvement in chest imaging, but still requiring antibiotic treatment). |

**Appendix 10: Definition of acute kidney injury**

| Study | Definition of acute kidney injury |
| --- | --- |
| Abdellatif,2016[7] | The acute renal failure (ARF) was defined as an increase of plasma creatinine more than 1.5 times its base value. |
| Ahn,2020[8] | Acute kidney injury (AKI) referred to the criteria for RIFLE (Risk, Injury, Failure, Loss, end-stage renal Failure). |
| Almangour,2021[9] | Risk, injury, failure, loss of kidney function, and end-stage kidney disease (RIFLE) criteria were used to assess renal function. Patients were considered to have nephrotoxicity if any of these categories were met during the course of treatment. |
| BOGOVIC,2014[20] | Nephrotoxicity was defined as a decline in renal function prompting renal replacement therapy, as a reduction in the calculated creatinine clearance of 50% from the baseline, as failure according to Risk, Injury, Failure, Loss, and End-stage kidney disease (RIFLE) criteria. |
| Cho,2016[1] | Acute kidney injury (AKI) was defined based on the RIFLE criteria, and patients were categorized into the R, I or F categories. |
| Choe,2019[11] | Nephrotoxicity was defined as a risk, injury, failure, loss of kidney function, and end-stage kidney disease (RIFLE) classification of injury or more, with injury defined as a greater than two-fold increase in serum creatinine, a greater than 50% reduction in glomerular filtration rate compared with the value at the start of treatment, or oliguria (⩽0.5 ml/kg/h) for ⩾12 h. |
| Hasan,2021[21] | The elevation of serum creatinine level above the normal range developed within forty-eight hours. |
| Jang,2017[3] | The primary outcome (nephrotoxicity) was evaluated based on the difference in creatinine levels from before and after colistin use. The Risk, Injury, Failure, Loss and End-stage kidney disease (RIFLE) criteria were also used to evaluate nephrotoxicity after colistin treatment. |
| Kalin,2012[12] | RIFLE (Risk, Injury, Failure, Loss, and End-stage kidney disease) criteria were used to evaluate the nephrotoxicity of COL. |
| Kim,2017[13] | In patients with normal renal function, AKI was defined as a serum creatinine level >2 mg/dL, or a ≥50% decrease in the glomerular filtration rate compared with the initial value at the start of treatment, or a deterioration of renal function that needed renal replacement therapy. In patients with preexisting renal dysfunction, AKI was defined as >50% of the baseline serum creatinine or a reduction in the calculated creatinine clearance of 50% compared with the initial value. |
| Zhou,2021[15] | NA |
| Lin,2022[16] | NA |
| Liu,2022[17] | Nephrotoxicity is the main side effect caused by the systematic administration of polymyxin B. Diagnosis of nephrotoxicity was based on risk, injury, failure, loss, end-stage kidney disease (RIFLE) criteria. At least over 1.5-fold increase in serum creatinine levels or a 25% decrease in calculated creatinine clearance from baseline and urine output less than 0.5 ml/kg/h over 6 h caused by intravenous polymyxin B was defined as nephrotoxicity.Proteinuria, tubular urine or azotemia caused by intravenous polymyxin B was also considered as nephrotoxicity. |
| Matijasevic,2018[22] | Acute renal failure defined by KDIGO -The Kidney Disease: Improving Global Outcomes definition. |
| Moghaddam,2019[4] | NA |
| Zheng,2019[6] | Increase in serum creatinine of S 0.3 mg/dL in 2 days or S 50% in 7 days after colistin treatment without other defined causes, such as septic shock, was defined as colistin associated nephrotoxicity. |
| Wu,2023[18] | According to the acute-kidney-injury (AKI) guideline from “2012 Kidney Disease: Improving Global Outcomes (KDIGO)”, AKI is defined using the following criteria: an increase in serum creatinine (SCr) of ≥0.3 mg/dL (≥26.5 μmol/L) within 48 h; or an increase in serum creatinine to ≥1.5 times baseline, which is known or presumed to have occurred within the previous 7 days; or a urine volume of < 0.5 mL/kg/h for 6 h. |
| Shi,2023[19] | Acute kidney injury (AKI) is defined as an increase in serum creatinine by 0.3 mg/dL within 48 h or by a 50% increase in serum creatinine within 7 days. Chronic kidney disease (CKD) was GFR <60 mL/min per 1.73 m2 for >3 months (Levey et al, 2020). Renal function is assessed with serum creatinine variations according to the classification of risk, injury, failure, loss, and end-stage of kidney disease (RIFLE) criteria. |

**Reference**

1. Cho AY, Yoon HJ, Lee JC, Kwak JY, Lee KY, Sun IO. Comparison of clinical characteristics of patients with acute kidney injury after intravenous versus inhaled colistin therapy. Kidney Res Clin Pract. 2016;35(4):229-232. <https://doi.org/10.1016/j.krcp.2016.07.005>

2. Doshi NM, Cook CH, Mount KL, Stawicki SP, Frazee EN, Personett HA, Schramm GE, Arnold HM, Murphy CV. Adjunctive aerosolized colistin for multi-drug resistant gram-negative pneumonia in the critically ill: A retrospective study. BMC Anesthesiol. 2013;13(1):45. <https://doi.org/10.1186/1471-2253-13-45>

3. Jang JY, Kwon HY, Choi EH, Lee WY, Shim H, Bae KS. Efficacy and toxicity of high-dose nebulized colistin for critically ill surgical patients with ventilator-associated pneumonia caused by multidrug-resistant Acinetobacter baumannii. J Crit Care. 2017;40:251-256. <https://doi.org/10.1016/j.jcrc.2017.04.004>

4. Moghaddam OM, Lahiji MN, Talebi-Taher M, Mahmoodiyeh B. Effect of inhaled colistin on the treatment of ventilator-associated pneumonia due to multi-drug resistant acinetobacter. Tanaffos. 2019;18(1):66-73.

5. Naesens R, Vlieghe E, Verbrugghe W, Jorens P, Ieven M. A retrospective observational study on the efficacy of colistin by inhalation as compared to parenteral administration for the treatment of nosocomial pneumonia associated with multidrug-resistant Pseudomonas aeruginosa. BMC Infect Dis. 2011;11:317. <https://doi.org/10.1186/1471-2334-11-317>

6. Zheng JY, Huang SS, Huang SH, Ye JJ. Colistin for pneumonia involving multidrug-resistant Acinetobacter calcoaceticus-Acinetobacter baumannii complex. J Microbiol Immunol Infect. 2020;53(6):854-865. <https://doi.org/10.1016/j.jmii.2019.08.007>

7. Abdellatif S, Trifi A, Daly F, Mahjoub K, Nasri R, Ben Lakhal S. Efficacy and toxicity of aerosolised colistin in ventilator-associated pneumonia: a prospective, randomised trial. Ann intensive care. 2016;6(1):26. <https://doi.org/10.1186/s13613-016-0127-7>

8. Ahn SH, Lee SJ, Ahn H-L, Hwangbo SY. Comparative Evaluation of Intravenous vs. Nebulized Colistin Treatment of Pneumonia Due to Multidrug-Resistant Acinetobacter baumannii & Pseudomonas aeruginosa. J Kor Soc Health-syst Pharm. 2020;37(1):11-19. <https://doi.org/10.32429/jkshp.2020.37.1.001>

9. Almangour TA, Alruwaili A, Almutairi R, Alrasheed A, Alhifany AA, Eljaaly K, Alkofide H, Alhammad AM, Ghonem L, Alsharidi A. Aerosolized plus intravenous colistin vs intravenous colistin alone for the treatment of nosocomial pneumonia due to multidrug-resistant Gram-negative bacteria: A retrospective cohort study. Int J Infect Dis. 2021;108:406-412. <https://doi.org/10.1016/j.ijid.2021.06.007>

10. Amin M, Rashad A, Fouad A, Abdel Azeem A. Re-emerging of colistin for treatment of nosocomial pneumonia due to gram negative multi-drug resistant pathogens in critically ill patients. Egypt J Chest Dis Tuberc. 2013;62(3):447‐451. <https://doi.org/10.1016/j.ejcdt.2013.05.012>

11. Choe J, Sohn YM, Jeong SH, Park HJ, Na SJ, Huh K, Suh GY, Jeon K. Inhalation with intravenous loading dose of colistin in critically ill patients with pneumonia caused by carbapenem-resistant gram-negative bacteria. Ther Adv Respir Dis. 2019;13:1753466619885529. <https://doi.org/10.1177/1753466619885529>

12. Kalin G, Alp E, Coskun R, Demiraslan H, Gundogan K, Doganay M. Use of high-dose IV and aerosolized colistin for the treatment of multidrug-resistant Acinetobacter baumannii ventilator-associated pneumonia: do we really need this treatment? J Infect Chemother. 2012;18(6):872-877. <https://doi.org/10.1007/s10156-012-0430-7>

13. Kim YK, Lee JH, Lee HK, Chung BC, Yu SJ, Lee HY, Park JH, Kim S, Kim HK, Kiem S *et al*. Efficacy of nebulized colistin-based therapy without concurrent intravenous colistin for ventilator-associated pneumonia caused by carbapenem-resistant Acinetobacter baumannii. J Thorac Dis. 2017;9(3):555-567. <https://doi.org/10.21037/jtd.2017.02.61>

14. Korbila IP, Michalopoulos A, Rafailidis PI, Nikita D, Samonis G, Falagas ME. Inhaled colistin as adjunctive therapy to intravenous colistin for the treatment of microbiologically documented ventilator-associated pneumonia: A comparative cohort study. Clin Microbiol Infect. 2010;16(8):1230-1236. <https://doi.org/10.1111/j.1469-0691.2009.03040.x>

15. Zhou L, Li C, Weng Q, Wu J, Luo H, Xue Z, Xiao Y, Chen C. Clinical study on intravenous combined with aerosol inhalation of polymyxin B for the treatment of pneumonia caused by multidrug-resistant Gram-negative bacteria. Chin Crit Care Med. 2021;33(4):416-420. <https://doi.org/10.3760/cma.j.cn121430-20201215-00753>

16. Lin H, Liu X, Sun P. Effects of Aerosol Inhalation Combined with Intravenous Drip of Polymyxin B on Bacterial Clearance, Symptoms Improvement, and Serum Infection Indexes in Patients with Pneumonia Induced by Multidrug-Resistant Gram-Negative Bacteria. Emerg Med Int. 2022;2022:5244538. <https://doi.org/10.1155/2022/5244538>

17. Liu J, Shao M, Xu Q, Liu F, Pan X, Wu J, Xiong L, Wu Y, Tian M, Yao J *et al*. Low-dose intravenous plus inhaled versus intravenous polymyxin B for the treatment of extensive drug-resistant Gram-negative ventilator-associated pneumonia in the critical illnesses: a multi-center matched case-control study. Ann Intensive Care. 2022;12(1):72. <https://doi.org/10.1186/s13613-022-01033-5>

18. Wu Z, Zhang S, Cao Y, Wang Q, Sun K, Zheng X. Comparison of the clinical efficacy and toxicity of nebulized polymyxin monotherapy and combined intravenous and nebulized polymyxin for the treatment of ventilator-associated pneumonia caused by carbapenem-resistant gram-negative bacteria: a retrospective cohort study. Front Pharmacol. 2023;14:1209063. <https://doi.org/10.3389/fphar.2023.1209063>

19. Shi R, Fu Y, Gan Y, Wu D, Zhou S, Huang M. Use of polymyxin B with different administration methods in the critically ill patients with ventilation associated pneumonia: a single-center experience. Front Pharmacol. 2023;14:1222044. <https://doi.org/10.3389/fphar.2023.1222044>

20. Bogović TZ, Budimir A, Bošnjak Z, Hrabač P, Baronica R, Tomašević B, Mirić M, Drvar Z, Pavlek M, Bratić V *et al*. Inhalation plus intravenous colistin versus intravenous colistin alone for treatment of ventilator associated pneumonia. Signa Vitae. 2014;9(SUPPL. 1):29-33.

21. Hasan MJ, Rabbani R, Anam AM, Santini A, Huq SMR. The Susceptibility of MDR-K. Pneumoniae To Polymyxin B Plus Its Nebulised Form Versus Polymyxin B Alone in Critically Ill South Asian Patients. J Crit Care Med (Targu Mures). 2021;7(1):28-36. <https://doi.org/10.2478/jccm-2020-0044>

22. Matijašević J, Gavrilović S, Andrijević I, Andrijević A, Milić S, Vukoja M. Inhalatory and intravenous colistin in treating ventilator-associated pneumonia due to Acinetobacter species: should we combine them? Vojnosanit Pregl. 2018;77(8):832-838. <https://doi.org/10.2298/vsp180910161m>
